# Fragment-based discovery of a new class of inhibitors targeting mycobacterial tRNA modification

**DOI:** 10.1093/nar/gkaa539

**Published:** 2020-06-30

**Authors:** Sherine E Thomas, Andrew J Whitehouse, Karen Brown, Sophie Burbaud, Juan M Belardinelli, Jasper Sangen, Ramanuj Lahiri, Mark Daben J Libardo, Pooja Gupta, Sony Malhotra, Helena I M Boshoff, Mary Jackson, Chris Abell, Anthony G Coyne, Tom L Blundell, Rodrigo Andres Floto, Vítor Mendes

**Affiliations:** Department of Biochemistry, University of Cambridge, 80 Tennis Court Road, Cambridge CB2 1GA, UK; Department of Chemistry, University of Cambridge, Lensfield Road, Cambridge CB2 1EW, UK; University of Cambridge Molecular Immunity Unit, MRC Laboratory of Molecular Biology, Francis Crick Avenue, Cambridge CB2 0QH, UK; Cambridge Centre for Lung Infection, Royal Papworth Hospital, Cambridge CB2 0AY, UK; University of Cambridge Molecular Immunity Unit, MRC Laboratory of Molecular Biology, Francis Crick Avenue, Cambridge CB2 0QH, UK; Mycobacteria Research Laboratories, Department of Microbiology, Immunology and Pathology, Colorado State University, Fort Collins, CO, USA; University of Cambridge Molecular Immunity Unit, MRC Laboratory of Molecular Biology, Francis Crick Avenue, Cambridge CB2 0QH, UK; National Hansen's Disease Program, Healthcare Systems Bureau, Health Resources and Services Administration, Department of Health and Human Services, Baton Rouge, LA, USA; Tuberculosis Research Section, Laboratory of Clinical Immunology and Microbiology, National Institute of Allergy and Infectious Disease, National Institutes of Health, 9000 Rockville Pike, Bethesda, MD 20892, USA; Department of Biochemistry, University of Cambridge, 80 Tennis Court Road, Cambridge CB2 1GA, UK; Birkbeck College, University of London, Malet Street WC1E7HX, UK; Tuberculosis Research Section, Laboratory of Clinical Immunology and Microbiology, National Institute of Allergy and Infectious Disease, National Institutes of Health, 9000 Rockville Pike, Bethesda, MD 20892, USA; Mycobacteria Research Laboratories, Department of Microbiology, Immunology and Pathology, Colorado State University, Fort Collins, CO, USA; Department of Chemistry, University of Cambridge, Lensfield Road, Cambridge CB2 1EW, UK; Department of Chemistry, University of Cambridge, Lensfield Road, Cambridge CB2 1EW, UK; Department of Biochemistry, University of Cambridge, 80 Tennis Court Road, Cambridge CB2 1GA, UK; University of Cambridge Molecular Immunity Unit, MRC Laboratory of Molecular Biology, Francis Crick Avenue, Cambridge CB2 0QH, UK; Cambridge Centre for Lung Infection, Royal Papworth Hospital, Cambridge CB2 0AY, UK; Department of Biochemistry, University of Cambridge, 80 Tennis Court Road, Cambridge CB2 1GA, UK

## Abstract

Translational frameshift errors are often deleterious to the synthesis of functional proteins and could therefore be promoted therapeutically to kill bacteria. TrmD (tRNA-(N(1)G37) methyltransferase) is an essential tRNA modification enzyme in bacteria that prevents +1 errors in the reading frame during protein translation and represents an attractive potential target for the development of new antibiotics. Here, we describe the application of a structure-guided fragment-based drug discovery approach to the design of a new class of inhibitors against TrmD in *Mycobacterium abscessus*. Fragment library screening, followed by structure-guided chemical elaboration of hits, led to the rapid development of drug-like molecules with potent *in vitro* TrmD inhibitory activity. Several of these compounds exhibit activity against planktonic *M. abscessus and M. tuberculosis* as well as against intracellular *M. abscessus and M. leprae*, indicating their potential as the basis for a novel class of broad-spectrum mycobacterial drugs.

## INTRODUCTION

Mycobacteria are a group of diverse organisms that include many important human pathogens such as *Mycobacterium tuberculosis*, responsible for over 1.5 million deaths per year from tuberculosis and *Mycobacterium leprae*, the causative agent of leprosy, both associated with increasing rates of acquired drug resistance (1,2). Furthermore, *Mycobacterium abscessus*, a rapidly growing species of nontuberculous mycobacteria (NTM), has recently emerged as a major threat to individuals with Cystic Fibrosis (CF) and other inflammatory lung conditions (3–5). *Mycobacterium abscessus* is intrinsically resistant to most existing antibiotics and as a consequence infections are associated with extremely high rates of treatment failure and mortality (5). There is, therefore, an urgent unmet need to develop new antibiotics against these mycobacterial infections. Hence, we examined the possibility of promoting translational frameshift errors as a novel approach to killing pathogenic mycobacteria.

**Figure 1.**
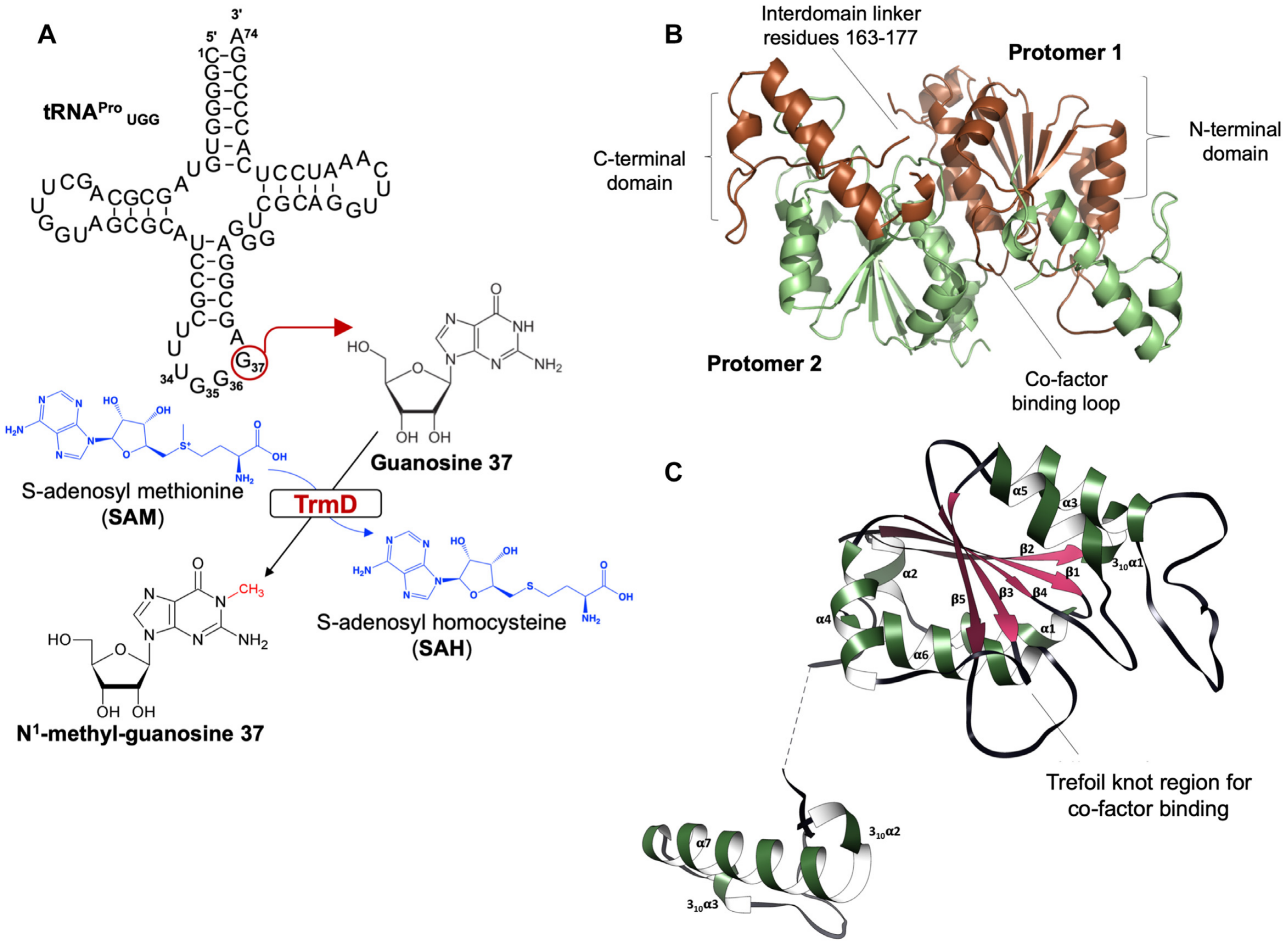
(**A**) TrmD reaction scheme illustrated with a cloverleaf model of *M. abscessus* UGG isoacceptor tRNA^Pro^. The modified Guanosine 37 base is indicated in red circle and the chemical reaction, mediated by TrmD, leading to the synthesis of N^1^-methyl guanosine 37 is illustrated with corresponding 2-D chemical structures in black. The chemical structures of the methyl donor *S*-adenosyl-l-methionine, which in turn gets converted into *S*-adenosyl-l-homocysteine, are shown in blue. (**B**) TrmD homodimer (PDB code 6NVR) with domain architecture illustrated. Protomers 1 and 2 are represented in brown and green ribbon diagrams respectively. (**C**) TrmD protomer is coloured and illustrated based on secondary structure elements. The disordered inter-domain linker is shown as black dotted lines.

Several structurally diverse, modified nucleosides found at different locations of tRNAs help in the maintenance of the reading frame and avoidance of translational frame-shift errors. Many such nucleoside modifications are found in regions near the anticodon, particularly at position 34 (the wobble position) and 37 (3′ and adjacent to the anticodon) of tRNA (6,7). TrmD, tRNA-(N(1)G37) methyltransferase, catalyzes the methylation of G_37_ (Guanosine at position 37) in prokaryotic tRNAs (Figure 1A). This modified nucleotide N^1^-methylguanosine at position 37 (m^1^G_37_) is present in tRNAs containing a G_36_G_37_ sequence in the anti-codon region from all three domains of life, where G_37_ is the base adjacent to the anticodon at the 3′ end (6,8,9). Mutations in *trmD* result in growth defects associated with increased translational frameshifting leading to defective protein production (7,9).

TrmD belongs to a distinct class of *S*-adenosyl-l-methionine (SAM)-dependent methyltransferases known as the SpoU-TrmD (SPOUT) RNA methyltransferase superfamily or Class IV methyltransferases. Proteins belonging to this family are structurally unique due to the absence of a consensus methyltransferase fold. TrmD and other proteins of the SPOUT family consist of a deep trefoil knot architecture at the catalytic region, which provides an L-shaped pocket for binding of SAM. In contrast, G_37_ methylation in eukaryotes is carried out by the enzyme Trm5 belonging to the Class I methyltransferase family (10–12).

Previous research (11,13) has shown that TrmD and Trm5 have distinct substrate requirements with RNA. While Trm5 recognizes the overall L-shaped tertiary structure of tRNA possessing a G_37_ base, TrmD recognition involves mainly the D stem and anticodon stem loop of tRNA with G_36_G_37_ bases. Trm5 functions as a monomer and binds to SAM at the Rossmann fold region of the active site, in contrast to dimeric TrmD with a trefoil knot methyl donor binding region. Further, SAM adopts a unique bent conformation in TrmD as compared to the extended conformation in Trm5 and many other canonical methyltransferases. These distinct structural features, substrate requirements and ligand binding conformations between TrmD and its human ortholog provide the potential for designing novel and selective inhibitors of bacterial TrmD (14). Further, a recent study (15) in gram-negative bacteria, demonstrates the crucial role played by m^1^G37 methylation of tRNA by TrmD in determining the synthesis of membrane proteins such as drug efflux pumps. This is achieved through regulation of protein translation at proline codons near the start of open-reading frames. Thus, the modification of tRNA by TrmD appears to be a global determinant of membrane protein synthesis at least in gram-negative bacteria and therefore inhibition of TrmD greatly impairs the development of drug resistance by sensitizing these organisms to antibiotics (15).

Previous drug discovery efforts targeting TrmD in *Haemophilus influenzae* (16) and *Pseudomonas aeruginosa* (17) led to the development of selective inhibitors with potent biochemical activity against TrmD isozymes *in vitro*. However, these compounds in general only showed weak antibacterial activity when profiled against a range of bacteria. We nevertheless wondered whether targeting TrmD, using a structure-guided fragment-based strategy, with systematic testing of biochemical and antimicrobial activities at each stage of iteration might prove successful in mycobacteria.

Fragment-based drug discovery (FBDD) is a promising approach for the development of new drugs, whereby the complexity of sampling chemical space is reduced by screening promiscuous small molecules (‘fragments’) for low affinity *in vitro* interactions. Initial fragment hits usually exhibit lower potency than the more complex drug-like molecules found in typical high-throughput screening compound libraries. However, such fragments bind by making well-defined and directional interactions, giving rise to highly ligand efficient (LE) molecules. These fragments can then be chemically optimized into lead candidates, thereby more effectively exploring the chemical space available for binding to the target protein (18–21). In this work we validate TrmD as a mycobacterial target and describe the application of an FBDD approach to generate a new family of small-molecule inhibitors of *M. abscessus* TrmD, having antimicrobial activities against a range of pathogenic mycobacteria.

## MATERIALS AND METHODS

### Allelic replacement

*Escherichia coli* DH5α, used for cloning, was grown in LB Lennox (BD, Difco) medium at 37°C. *Mycobacterium abscessus* ssp. *massiliense* CIP108297 was grown in Middlebrook 7H9-ADC broth (BD, Difco) supplemented with 0.05% Tween 80 or 7H11-ADC agar (BD, Difco) at 37°C. Kanamycin (Kan), streptomycin (Str) and hygromycin (Hyg) were added to final concentrations of 200, 200 and 2000 μg/ml, respectively. Homologous recombination at the *trmD* locus of *M. abscessus* ssp. *massiliense* CIP108297 was performed using a mycobacterial recombinase-based system in which the recombineering genes from mycobacteriophage Che9c (22) are expressed from the replicative plasmid pNitET-*xylE*-kan (a derivative of the pNitET-*sacB*-kan plasmid (23) generated in-house in which the *sacB* gene was replaced by the *xylE* colored marker) under control of an isovaleronitrile-inducible promoter. Isovaleronitrile-induced *M. abscessus* ssp. *massiliense* CIP108297 cells harboring pNitET-*xylE*-kan were electro-transformed with ∼300 ng of linear allelic exchange substrate consisting of the streptomycin-resistance cassette from pHP45Ω flanked by 1000 bp of DNA sequence immediately flanking the start and stop codons of *trmD*, and double-crossover mutants were isolated on Str-containing agar. Allelic replacement leading to the complete deletion of the *trmD* locus was checked by PCR using a pair of primers annealing outside the linear allelic exchange substrate.

Plasmid pMV306H was constructed by replacing the kanamycin-resistance cassette of pMV306hsp (an integrative mycobacterial expression vector allowing for the expression of genes under control of the hsp60 promoter; Addgene plasmid # 26155) (24) by a hygromycin-resistance cassette. pMV306H::trmD was generated by cloning the PCR-amplified *trmD* gene from *M. abscessus* ssp. *massiliense* CIP108297 in the HindIII site of pMV306H. All primer sequences are shown in Supplementary Table S1.

### Expression and purification of full-length *M. abscessus* TrmD

*Escherichia coli* BL21 (DE3) strain containing AVA0421 plasmid with an N-His-3C Protease site-TrmD full-length insert, kindly provided by the Seattle Structural Genomics Consortium, (25) was grown overnight at 37°C in LB-media containing Ampicillin (100 μg/ml). This seed stage culture was used to inoculate six shake flasks containing 1 l each of 2XYT media with Ampicillin (100 μg/ml) until optical density (*A*_600 nm_) reached 0.6. The expression of recombinant construct was induced by the addition of isopropyl β-d-1-thiogalactopyranoside (IPTG) to a final concentration of 0.5 mM and further allowed to grow at 18°C for 16 h.

#### Isolation of cells and lysis

Cells were harvested by centrifugation at 4°C for 20 min at 5000 g and the pellet was re-suspended in buffer A (25 mM HEPES pH 7.5, 500 mM NaCl, 5% glycerol, 10 mM MgCl_2_, 1 mM TCEP, 20 mM imidazole). 0.1% Triton (Sigma), 10 μg/ml DNaseI, 5 mM MgCl_2_ and three protease inhibitor cocktail tablets (New England Biolabs) were added to the cell suspension. The cells were lysed in an Emulsiflex (Glen Creston) and clarified the lysate by centrifugation at 4°C for 40 min at 25 568 g.

#### Immobilized metal affinity chromatography

The clarified lysate was filtered using a 0.45 μm syringe filter and passed through a pre-equilibrated (with buffer A), 10 ml pre-packed nickel- sepharose column (HiTrap IMAC FF, GE Healthcare). The column was washed with 5 column volumes of buffer A and the bound protein was eluted as 4 × 10 ml elutes using buffer B (25 mM HEPES pH 7.5, 500 mM NaCl, 5% glycerol, 1 mM TCEP, 500 mM imidazole). The protein was analyzed on a 15% SDS-PAGE gel. *Dialysis*: Elutes from Hi-Trap IMAC column were pooled, added 3C Protease in the ratio of 1:50 mg (protease: protein) and subjected to dialysis against 2 l of buffer C (25 mM HEPES pH 7.5, 500 mM NaCl, 5% glycerol, 1 mM TCEP) overnight at 4°C.

Protein, after overnight dialysis and cleavage of N-His tag, was passed through a pre-equilibrated (buffer A) 5 ml HiTrap IMAC FF Nickel column (GE Healthcare).

#### Size exclusion chromatography

The flow through from the above column was concentrated to 3 ml using a 10 kDa centrifugal concentrator (Sartorius Stedim) and loaded onto a pre-equilibrated (with buffer D: 25 mM HEPES pH 7.5, 500 mM NaCl, 5% glycerol) 120 ml Superdex200 16/600 column (GE Healthcare). 2 ml fractions were collected and analyzed on a 15% SDS-PAGE gel. Fractions corresponding to pure TrmD protein were pooled and concentrated to 25 mg/ml, flash frozen in liquid nitrogen and stored at –80°C. Identity of the purified protein was further confirmed by MALDI mass fingerprinting.

### Crystallization of apo form of full-length *M. abscessus* TrmD

*M. abscessus* TrmD apo crystals were grown in 48-well sitting drop plates (Swiss CDI) in the following condition: 0.08 mM Sodium cacodylate pH 5.8 to 6.8, 1–2 M ammonium sulphate. 24 mg/ml of the protein in storage buffer (25 mM HEPES pH 7.5, 500 mM NaCl, 5% glycerol) at drop ratio 1 μl:1 μl (protein:reservoir respectively) were set up and equilibrated against 70 μl reservoir.

### Soaking of TrmD native crystals with fragments and ligands

Crystals for this experiment were grown at 19°C in 48-well sitting drop plates (Swiss CDI) in the following condition: 0.08 mM Sodium cacodylate pH 6.5 to 7.0, 1–2 M ammonium sulphate, 20 mg/ml of the protein in storage buffer (25 mM HEPES pH 7.5, 500 mM NaCl, 5% glycerol) at drop ratio 1 μl:1 μl were set up and equilibrated against 250 μl reservoir. Further, the crystals were picked and allowed to soak in a 4 μl drop containing reservoir solution and 10 mM fragments/compound (in DMSO) which was then equilibrated against 700 μl of the corresponding reservoir solution overnight at 19°C in 24-well hanging drop vapor diffusion set up.

### Co-crystallization of TrmD protein with SAM/ SAH/ AW6/ AW7

2–5 mM final concentration of compound in DMSO/water was added to 20 mg/ml of TrmD protein, mixed and incubated for 2 h on ice. Crystals were grown in the following condition: 0.08 mM Sodium cacodylate pH 6.5 to 7.0, 1–2 M Ammonium sulphate or in sparse matrix screens: Wizard 1&2 (Molecular Dimensions), Wizard 3&4 (Molecular Dimensions), JCSG +Suite (Molecular Dimensions). The crystallization drops were set up at a protein to reservoir drop ratio of 0.3 μl:0.3 μl, in 96-well (MRC2) sitting drop plate, using mosquito crystallization robot (TTP labtech) and the drops were equilibrated against 70 μl of reservoir at 19°C.

### X-ray data collection and processing

The TrmD apo/ligand-bound crystals were cryo-cooled in mother liquor containing 27.5% ethylene glycol. X-ray data sets were collected on I04, I02, I03, I04-1 or I24 beamlines at the Diamond Light Source in the UK, using the rotation method at wavelength of 0.979 Å, Omega start: 0°, Omega Oscillation: 0.1–0.2°, Total oscillation: 210–240°, total images: 2100–2400, Exposure time: 0.05–0.08 s. The diffraction images were processed using AutoPROC (26), utilizing XDS (27) for indexing, integration, followed by POINTLESS (28), AIMLESS (29) and TRUNCATE (30) programs from CCP4 Suite (31) for data reduction, scaling and calculation of structure factor amplitudes and intensity statistics. All TrmD crystals belonged to space group *P*2_1_2_1_2_1_ and consisted of two protomers in the asymmetric unit.

### Structure solution and refinement

The *M. abscessus* TrmD Apo structure was solved by molecular replacement using PHASER (32) with the atomic coordinates of *M. abscessus* TrmD at 1.7 Å (PDB entry: 3QUV Seattle Structural Genomics Consortium for Infectious Diseases) as search model and TrmD ligand bound structures were solved by molecular replacement with the atomic coordinates of the solved *M. abscessus* TrmD Apo structure (PDB entry: 6NVR) as search model. Structure refinement was carried out using REFMAC (33) and PHENIX (34).

The models obtained were manually re-built using COOT interactive graphics program (35) and electron density maps were calculated with 2|*F*_o_| – |*F*_c_| and |*F*_o_| – |*F*_c_| coefficients. Positions of ligands and water molecules were located in difference electron density maps and OMIT difference maps |m*F*_o_ − D*F*_c_| (36) were calculated and analysed to further verify positions of fragments and ligands.

### Differential scanning fluorimetry (DSF)

DSF were carried out in a 96-well format with each well containing 25 μl of reaction mixture of 10 μM TrmD protein in buffer (50 mM HEPES pH 7.5, 500 mM NaCl, 5% glycerol), 5 mM compound, 5% DMSO and 5× Sypro orange dye. Appropriate positive (Protein, DMSO and SAM) and negative (Protein, DMSO only) controls were also included. The measurements were performed in a Biorad-CFX connect thermal cycler using the following program: 25°C for 10 min followed by a linear increment of 0.5°C every 30 s to reach a final temperature of 95°C. The results were analyzed using Microsoft excel.

### Isothermal titration calorimetry (ITC)

ITC experiments to quantify binding of ligands to TrmD were done as described in (37) using Malvern MicroCal iTC200 or Auto-iTC200 systems at 25°C. Titrations consisted of an initial injection (0.2 μl), discarded during data processing, followed by either 19 (2 μl) or 39 (1 μl) injections separated by intervals of 60–150 s duration. Protein was dialysed overnight at 4°C in storage buffer (*M. abscessus* TrmD: 50 mM HEPES pH 7.5, 500 mM NaCl, 5% glycerol; *M. tuberculosis* TrmD: 25 mM HEPES pH 7.5, 500 mM NaCl). Sample cell and syringe solutions were prepared using the same storage buffer, with a final DMSO concentration of 2–10% according to ligand solubility in the buffer. TrmD concentrations of either 33 or 100 μM were used, with ligand to protein concentration ratios ranging from 10 to 20:1. Control titrations without protein were also performed and subtracted from ligand to protein titrations. Titrations were fitted with Origin software (OriginLab, Northampton, MA, USA), using a one-site binding model with *N* fixed to 1 only for weakly binding ligands. Titrations were typically performed once (*n* = 1), with multiple isotherms obtained (*n* > 1) for key compounds of interest. *K*_d_ values are reported to two significant figures. Error provided by Origin software due to model fit is reported when *n* = 1, whereas standard deviation is reported when *n* > 1.

### Chemical synthesis of compounds

The compounds **AW1-7** were synthesised according to the procedures described in the supporting information. A more in-depth discussion around the medicinal chemistry strategy for fragment merging and lead compound development is described in a corresponding publication by Whitehouse *et al.* (37). The compounds **AW1-7** are listed in this publication as follows: **AW1** (Compound **23**), **AW2** (Compound **24f**), **AW3** (Compound **26f**), **AW4** (Compound **28**), **AW5** (Compound **29a**), **AW6** (Compound **31a**) and **AW7** (Compound **29d**).

### Biochemical activity assays

Assays for quantifying TrmD methylation reactions were carried out in 20 μl reactions consisting of 6.25 μM SAM, 0.1 μM TrmD and 6.25 μM tRNA^Pro^_UGG_ in the presence of 0–500 μM compounds in serial dilutions using assay buffer containing 50 mM Tris–HCl pH 7.5, 10 mM MgCl_2_, 24 mM NH_4_Cl, 5% DMSO and 1 mM DTT in nuclease free water. tRNA sequences were identified from the *M. abscessus* genome sequence using tRNAscan-SE algorithm, (38,39). The substrate *M. abscessus* tRNA^Pro^_UGG_ for the assay, having the sequence 5′-CGGGGUGUAGCGCAGCUUGGUAGCGCAUCCGCUUUGGGAGCGGAGGGUCGCAGGUUCAAAUCCUGUCACCCCGA-3′, was purchased commercially from Integrated DNA technologies (USA). The reactions were carried out for 1 h at room temperature followed by addition of 20 mM EDTA to stop the reactions. Each of the 20 μl samples were diluted ten-fold with the UPLC mobile phase solvent A (0.1% formic acid in water), centrifuged for 10 min at 13 000 g, to remove any precipitates, and the supernatant was aliquoted into 96-well plates. 40 μl samples were then injected into Acquity UPLC (Waters) T3 1.8 μM column and eluted using a gradient elution consisting of Mobile Phase A: 0.1% formic acid in water and mobile phase B: 0.1% formic acid in 100% methanol for 4 min. The absorbance was monitored using a photodiode array (PDA) detector (Waters) at wavelength range of ƛ: 220–500 nm. All reactions were carried out in triplicate. The blank corrected data were analyzed using Microsoft excel and non-linear regression analysis for IC_50_ determination were done using GraphPad prism version 7.00, GraphPad Software, La Jolla, CA, USA.

### Mycobacterial strains used and MIC measurements

*Mycobacterium abscessus* ssp. *abscessus* (ATCC 19977) transformed with pmv310 plasmid expressing Lux ABDCE operon, grown in Middlebrook 7H9 broth supplemented with ADC (Sigma, UK). All the other NTM strains are clinical isolates. Minimum inhibitory concentrations (MIC) were determined for mycobacteria according to the Clinical and Laboratory Standards Institute (CLSI) method M07-A9. Briefly, mycobacteria were grown to optical density (*A*_600 nm_) of 0.2–0.3 in liquid culture and 1 × 10^5^ bacteria were added to each well of 96-well plates containing serial dilutions of compound (400, 200, 100, 50, 25, 12.5, 6.3, 3.1, 1.6, 0.8, 0.4, 0 μM), in triplicate wells per condition, and incubated at 37°C until growth was seen in the control wells. MIC measurements using *M. tuberculosis* H37Rv were performed as reported in (37). *M. tuberculosis* H37Rv was grown in Middlebrook 7H9 base containing 14 mg/l dipalmitoyl phosphatidylcholine (DPPC), 0.81 g/l NaCl, 0.3 g/l casitone, and 0.05% Tyloxapol. H37Rv was grown and diluted to a similar inoculum size as mentioned above prior to exposure to serial dilutions of compounds (starting at 100 μM), and the plates were incubated at 37°C for 2 weeks. The MIC value was determined as the last well which showed no bacterial growth.

### CRISPR–dCas9 knockdown in *M. abscessus*

The dCas9 encoding plasmid (pTetInt-dcas9-Km) and the second vector containing the sgRNA cassette (pGRNAz) were derived from the tetracycline inducible CRISPr Interference system of Choudhary *et al.* (40) and optimized for *M. abscessus* ATCC19977. The 20 nucleotides guides targeting yidC(MAB_4953c) and trmD(MAB_3226c) were annealed and cloned in between sphI and aclI of the pGRNAz. As a control the pGRNAz was left empty. The CRISPr-I containing strains were cultivated in Middlebrook 7H9 broth supplemented with 1× ADC, 0.05% tween80 and 0.5% glycerol, hygromycin 1 mg/ml and zeocin 300 μg/ml. The cultures were inoculated at 10^6CFU/ml from an exponentially grown pre-cultures. The AW7 compound and doxycycline were added or not at a concentration of 25μM and 1.5625, 6.25, 25 or 100 ng/ml respectively. The OD_600_ was measured and the CFU counted at 72 h.

### Macrophage infection study

Blood samples were donated by healthy volunteers who had undertaken informed consent in accordance with local Research Ethics Committee approval. Peripheral blood mononuclear cells were isolated from citrated peripheral blood samples by density gradient separation using Lympholyte (Cedarlane Labs), and subsequent CD14^+^ positive selection using the MACS Miltenyi Biotec Human CD14 microbead protocol (Miltenyi Biotec). CD14^+^ cells were differentiated into macrophages using recombinant human granulocyte-macrophage colony-stimulating factor (200 ng/ml GM-CSF) and recombinant human interferon gamma (50 ng/ml IFN_γ_) (Peprotech) in standard tissue culture DMEM media containing fetal calf serum, penicillin and streptomycin. Following removal of antibiotics, macrophages were infected at a multiplicity of infection of 10:1 with *M. abscessus* 19977 for 2 h, washed in sterile phosphate buffered saline, and then incubated in DMEM media with FCS and 25 μM of compound for 24 and 48 h. At the given time points, supernatant was saved for cell cytotoxicity studies, and *M. abscessus* survival within the macrophages calculated by macrophage lysis in sterile water, and colony forming unit calculation on Columbia Blood Agar plates (VWR BDH).

### Cytotoxicity

Lactate dehydrogenase (LDH) was measured as a biomarker for cellular cytotoxicity using the Pierce LDH Cytotoxicity Assay Kit. Cell supernatant was measured at 2, 24 and 48 h post-infection according to the kit protocol.

### Nude mouse derived *M. leprae*

*Mycobacterium leprae* (isolate Thai-53) was maintained in serial passage in the foot pads of athymic nude mice (Envigo, USA). Mice were inoculated in the plantar surface of both hind feet with 5 × 10^7^ fresh, viable nude mice derived *M. leprae*. When the mouse foot pads became moderately enlarged (at ∼5–6 months), they were harvested for intracellular *M. leprae* as described previously (41), washed by centrifugation, re-suspended in medium, enumerated by direct count of acid fast bacilli according to Shepard's method (42), held at 4°C pending quality control tests for contamination and viability (41). Freshly harvested bacilli were always employed in experiments within 24 h of harvest.

### *M. leprae* axenic culture

Freshly harvested nude mouse foot pad derived *M. leprae* were suspended in modified 7H12 medium, **AW7** was added at different concentrations (100–6.25 μM) and were incubated for 7 days at 33°C. Media only and rifampin (Sigma, USA) at 2.4 μM were used as negative and positive controls. Following incubation aliquots of **AW7** treated and control *M. leprae* were processed for radiorespirometry (RR) as described previously (43).

### *M. leprae* macrophage culture

Bone marrow cells were obtained aseptically from both femurs of female BALB/c mice and cultured on plastic cover slips in Dulbecco modified Eagle's medium (DMEM, Life Technologies, USA) supplemented with 10% (v/v) fetal calf serum (Life Technologies), 25 mM/l HEPES (Sigma, USA), 2 mM/l glutamine (Sigma, USA), 50 μg/ml ampicillin (Sigma, USA) and 10 ng/ml of recombinant murine macrophages colony stimulating factor (R&D Systems, USA) for 6–7 days at 37°C and 5% CO_2_. The cells were infected with freshly harvested nude mice foot pad derived live *M. leprae* at a multiplicity of infection (MOI) of 20:1 overnight at 33°C and then washed to remove extracellular bacteria. **AW7** was added at different concentrations (100–6.25 μM) and the cells were incubated for 7 days at 33°C. Media only and rifampicin at 2.4 μM were used as negative and positive controls. **AW7** treated and control cells were lysed with sodium dodecyl sulfate (SDS, 0.1% w/v, Sigma, USA) and the intracellular *M. leprae* processed for radiorespirometry (44).

### Radiorespirometry

Metabolism of a suspension of *M. leprae* was measured by evaluating the oxidation of ^14^C-palmitic acid to ^14^CO_2_ by radiorespirometry as described previously (45). Levels of captured ^14^CO_2_ is proportional to the rate of ^14^C-palmitic acid oxidation and used as an indicator of *M. leprae* viability. In the present study the seventh day cumulative counts per minute (CPM) were recorded and percentage inhibition of metabolism determined as compared to no drug control. Statistical significance between treatment groups and no drug control were determined by Student's *t*-test and *P* < 0.05 is considered as significant.

## RESULTS

### TrmD is essential for *viability* of *M. abscessus*

Although previous transposon mutagenesis studies have suggested that *trmD* is essential in *M. tuberculosis* (46–48), confirmation of essentiality in *M. abscessus* was previously lacking. Three initial attempts to disrupt the *trmD* gene of *M. abscessus* by homologous recombination using a recombineering approach resulted in no colony growth. To confirm that *trmD* gene is essential for *M. abscessus* viability, allelic replacement experiments were repeated in merodiploid *M. abscessus* expressing a second copy of the *trmD* gene from the integrative plasmid pMV306H::*trmD*, or in empty vector controls. Analysis of over 100 candidate mutants in each background, from two independent experiments, showed that endogenous chromosomal *trmD* could only be knocked-out in the presence of an extra-copy of the gene (Supplementary Figure S1), confirming *trmD* essentiality in *M. abscessus*.

### *M. abscessus* TrmD: overall structure and ligand binding

We determined the crystal structures of *M. abscessus* TrmD in apo form at 1.60 Å resolution (PDB code: **6NVR**), as well as in complex with SAM and *S*-adenosyl-l-homocysteine (SAH) at 1.67 and 1.48 Å resolution respectively (PDB codes: **6NW6** and **6NW7**). Data collection and crystallographic statistics are given in Supplementary Table S2. The crystals belong to space group *P*2_1_2_1_2_1_ and consist of a homodimer in the asymmetric unit. Each non-crystallographic 2-fold symmetry-related protomer of TrmD interacts in an antiparallel manner and consists of two domains: a larger *N*-terminal domain spanning residues 1–161 and a smaller *C*-terminal helical domain (177–242) connected by a flexible inter-domain linker. The two domains of the individual protomers do not contact each other and the inter-domain region is largely disordered, with residues 162–177 not clearly visible in the apo structure (Figure 1B and C).

**Figure 2.**
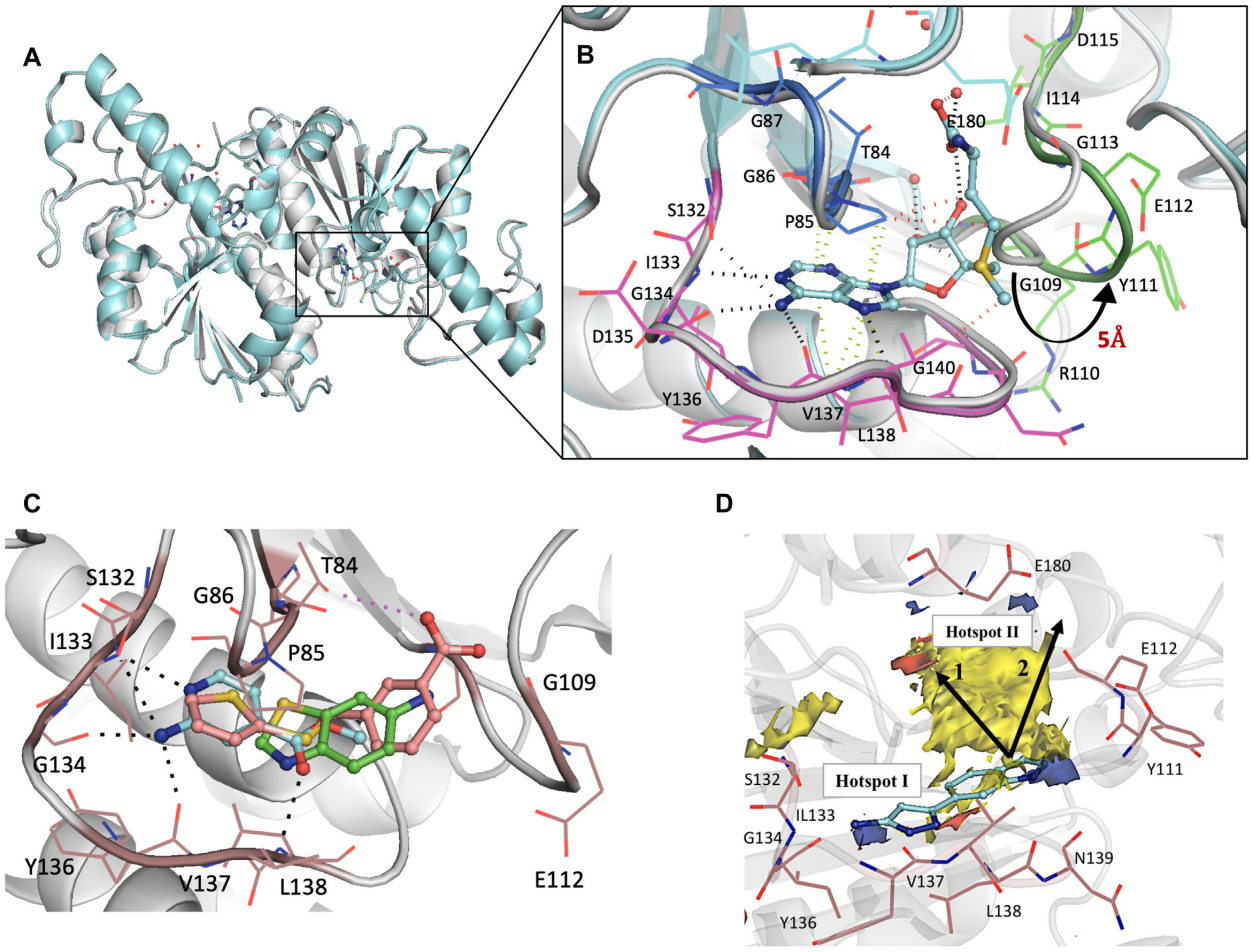
(**A**) Structural superposition of TrmD apo form (white) and TrmD SAM bound form (light blue), PDB codes 6NVR and 6NW6 respectively (**B**) the trefoil-knot active site of TrmD involving: cover loop (residues 84–87) shown in dark blue and bottom loop (residues 132–140) in magenta and wall loop (residues 109–115) in green and the conformational flip of the wall loop (residues ^109^GRYEGID^115^) upon SAM (light blue stick) binding are illustrated. The residues corresponding to each loop region are also shown as line representation. (**C**) Three representative fragment hits from each cluster, fragment **14** (PDB Code 6QOK) coloured in blue, fragment **20** (PDB Code 6QOQ) in green and fragment **8** (PDB Code 6QOE) in salmon respectively, occupying the TrmD SAM binding site. Major Hydrogen bonds and electrostatic interactions are depicted in black and purple dotted lines respectively. (**D**) Hot spot map contoured at 14 of TrmD active site superposed with crystal structure of TrmD in complex with merged compound **AW1** (light blue stick). Donor, acceptor and hydrophobic regions of the map are depicted as blue, red and yellow regions respectively. Amino acid residues contributing towards interactions in each hotspot map region are shown as brown stick representation. The arrows indicate two potential ways of fragment elaboration.

The SAM binding region of TrmD is located at the base of the *N*-terminal domain and consists of a deep trefoil knot architecture, made of three distinct untwisted loop regions. The trefoil knot of *M. abscessus* TrmD is made up of a cover loop spanning residues ^84^TPAG^87^ between strand β3 and helix α4 leading to the wall loop at the edge of the methionine pocket containing residues ^109^GRYEGID^115^ between β4 and helix α5. This loop then crosses over to form the bottom loop with residues 132–140 that encompasses the SAM adenine ring between strand β5 and helix α6 (Figure 2A and B). SAM and SAH occupy the deep trefoil-knot active site at the base of the *N*-terminal region and adopt an L-shaped bent conformation as previously observed with other TrmD orthologs (49,50). Both SAM and SAH form an extensive hydrogen-bonding network in this region along with hydrophobic and π-interactions as shown in Figure 2B & Supplementary Figure S2. The adenine ring of SAM and SAH is sandwiched between the cover loop and bottom loop of the knot with the adenine N1 and N7 forming hydrogen-bond contacts with the backbone amide-nitrogen atoms of Ile133 and Leu138 respectively, while the amino nitrogen forms additional hydrogen bonding contacts with the backbone carbonyl oxygen atoms of Gly134 and Tyr136 of the bottom loop (Figure 2B and Supplementary Figure S2).

The ribose and methionine moieties of SAM and SAH interact with the wall and bottom loops of the knot. The hydroxyl oxygen atom (O2′) of the ribose ring forms a hydrogen bond with the backbone amide of Gly109. The methionine and homocysteine moieties further extend into the active site groove formed between the cover and wall loops, making further hydrogen-bonding interactions with water molecules in this region (Figure 2B and Supplementary Figure S2).

A structural superposition of the apo and SAM bound forms of TrmD reveals the wall loop undergoing a switch in conformation leading to a movement of about 5 Å, when measured at the Cα of Tyr111, to the outer edge of subunit A. This conformational flip of the wall loop from apo form to SAM-bound form and the subsequent change in positions of residues 110–113 help to accommodate the methionine moiety of the methyl donor (Figure 2A and B).

### Fragment screening, hit validation and clustering of fragments

Having examined the conformational changes and binding interactions at the *M. abscessus* TrmD catalytic site, we initiated a structure-guided FBDD effort targeting *M. abscessus* TrmD by screening an in-house library of 960 small molecule fragments. The preliminary screening was performed using differential scanning fluorimetry (DSF), resulting in 53 hits within a thermal shift cut-off value of 3 standard deviations from the negative control (the TrmD protein and DMSO in the absence of ligand). These hits were then selected for validation by X-ray crystallography. Apo crystals of *M. abscessus* TrmD were soaked with each of the 53 fragments in independent experiments. The resulting crystal structure determinations allowed characterization of the binding modes of 27 fragments (Supplementary Figure S3).

All of the 27 fragments validated by X-ray crystallography were found to occupy the TrmD SAM site. These fragments can be clustered into three groups based on their binding mode at this site (Figure 2C and Supplementary Figure S3). *Cluster 1* consists of 12 fragments that bind exclusively to the sub-pocket that accommodates the adenine ring of SAM, engaging residues within the cover and bottom loops of the trefoil knot. These fragments recapitulated many of the hydrogen bonding and π-interactions of the SAM adenine moiety, as shown in the example (Figure 2C and Supplementary Figure S3). These interactions include hydrogen-bond contacts to the side chain of Ser132, which in turn adopts a dual conformation, and to the backbone amides of Ile133, Gly134, Tyr136 and Leu138.

The second cluster consists of 12 further fragments that occupy the entire adenosine region of the TrmD active site, thus extending from adenine towards the ribose-binding pocket of the active site. These fragments, in addition to retaining several adenine moiety contacts, also interact with the wall loop residues, forming hydrogen bonds to the backbone amides of Tyr111 and Gly109 and water-mediated hydrogen bonds as shown in the example (Figure 2C and Supplementary Figure S3).

*Cluster 3* consists of three fragments that extend beyond the TrmD adenosine site, thus reaching the methionine-binding region of the pocket. One of these fragments, Fragment 8 stretched further into the groove formed between the cover and wall loops of the trefoil knot, thus engaging additional hydrogen bonding contacts with the side chain of Thr84 and the backbone amide of Gly109 in this region (Figure 2C and Supplementary Figure S3).

### Fragment merging and hotspot mapping for chemical elaboration

**Figure 3.**
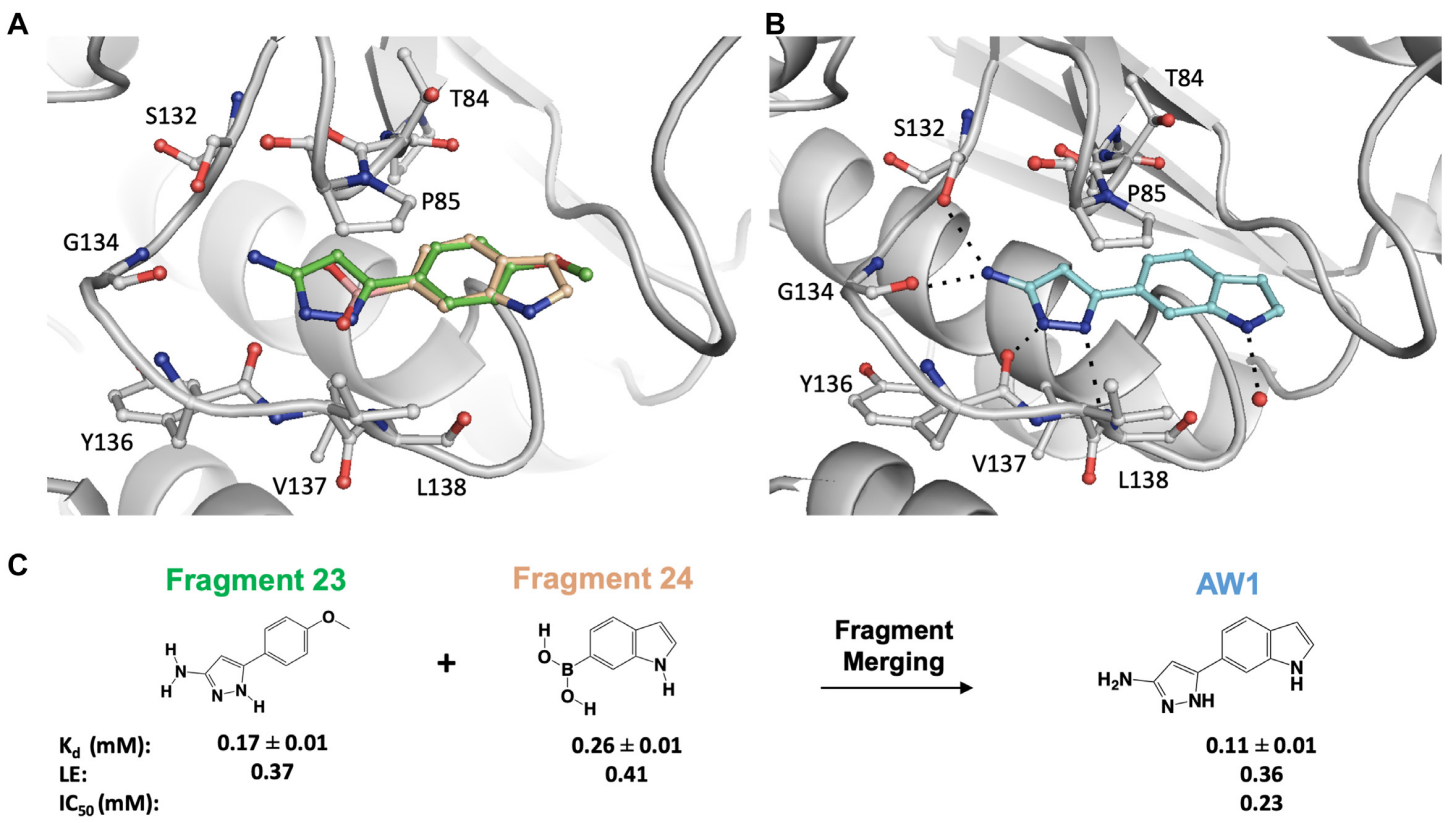
Fragment merging approach. (**A**) Structural superposition of TrmD (gray) in complex with fragments **23** (green) and **24** (beige) – PDB Codes 6QOT and 6QOU, showing binding mode and interactions at the SAM site (**B**) merged compound **AW1** (light blue), PDB Code 6QQS, showing binding mode at the SAM site. The corresponding amino acid interactions are illustrated in dotted lines and (**C**) the overall scheme of fragment merging.

Two of the above 27 fragment hits, fragments **23** (*K*_d_ 0.17 mM, LE 0.37) and **24** (*K*_d_ 0.26 mM, LE 0.41), were chosen for further chemical development by a fragment-merging strategy. The choice of fragments for subsequent chemical optimization was based on a number of criteria, including binding affinity, ligand efficiency, synthetic tractability and their ability to make key binding interactions at the TrmD SAM binding site. Fragment **23** occupies the adenosine binding region of the TrmD AdoMet site, with its pyrazole ring making hydrogen bond contacts to the backbone amides of Tyr136 and Leu138 and the amino group making further hydrogen bonds with the backbone carbonyl oxygen of Gly134 and the side chain of Ser132, respectively. The 4-methoxyphenyl ring of the fragment extends into the ribose binding site, engaging hydrophobic and π-interactions with the residues of the cover loop (Figure 3A, Supplementary Figures S3 and S4).

The indole ring of Fragment **24** also occupies the ribose pocket where it forms a water-mediated interaction with the backbone amide nitrogen of Leu138. The 4-methoxyphenyl and indole ring systems of Fragments **23** and **24** overlap perfectly, while the 6-boronic acid group of fragment **24** partially extends into the SAM adenine pocket and makes hydrogen bonds with the backbone amides of residues Tyr136 and Leu138 and further water-mediated hydrogen bond contacts to the backbone amides of Val131, Ile133, Gly134 and the side chain hydroxyl group of Ser132 (Figure 3A and Supplementary Figure S3). Compound **AW1** (*K*_d_ 0.11 mM, LE 0.36, IC_50_ 0.23 mM), formed by merging the two fragments, adopts a similar conformation to that of the original fragments in the TrmD SAM site, as shown in Figures 3B, C and Supplementary Figure S4, thereby providing a new chemical scaffold for further structure-guided development.

To aid the structure-guided lead discovery, we examined the ligand binding propensities of TrmD using hotspot mapping, a computational approach developed by Randoux, Blundell, and colleagues (51). Hotspots are areas within the protein that provide relatively large contributions to the overall binding affinity of ligands (52,53).

While the observed fragment hits and the corresponding merged compound **AW1** satisfy many of the predicted protein-hotspot interactions, the map also suggested further potential interactions that could stabilise elaborated fragments. As shown in Figure 2D, **AW1** occupies Hotspot 1 at the base of the TrmD active site, where it satisfies the hydrogen-bond donor requirements by interacting with the backbone amide oxygen atoms of Gly134 and Tyr136. The merged compound **AW1** also orients its pyrazole nitrogen atom in the acceptor map in this region where it forms a hydrogen bond with the backbone NH of Leu138 (Figures 2D and 3B). The compound could be elaborated further towards the methionine end of the active site and by further extension to the second hotspot region at the top of the active site. The second hotspot is characterized by a large hydrophobic patch surrounded by the acceptor region mediated by the backbone amide group and side chain of Glu180. A second approach to fragment elaboration would be by growing further upwards from the hydrophobic region of Hotspot 2 over to the donor region mainly mediated by the backbone oxygen atom and side chains of Glu112 as illustrated in Figures 2D and 3B.

### Structure-based lead optimization of merged compounds

**Figure 4.**
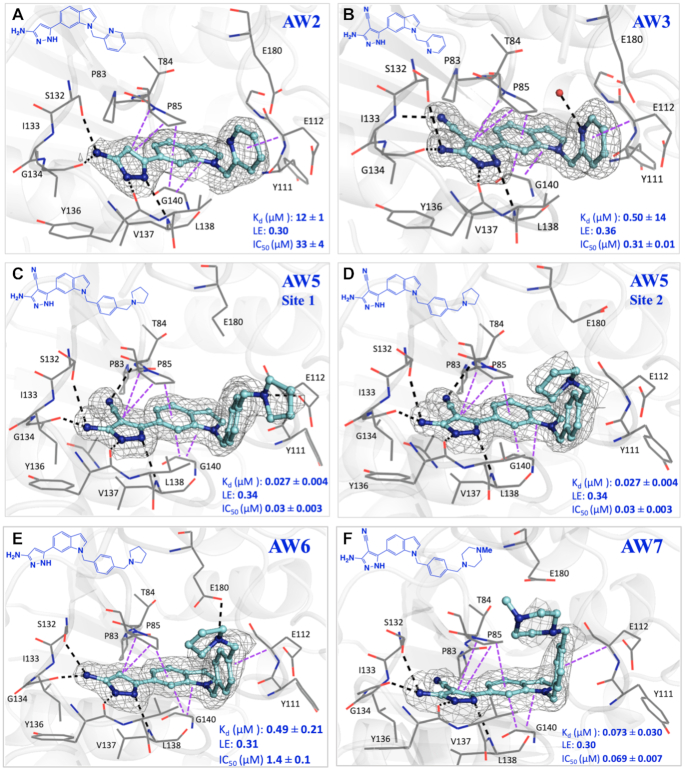
X-ray crystal structure of TrmD (grey) in complex with compounds (**A**) **AW2**, PDB Code 6QQX (**B**) **AW3**, PDB Code 6QQY (**C**) **AW5** at protomer 1, PDB Code 6QR6, (**D**) **AW5** at protomer 2, PDB Code 6QR6, (**E**) **AW6**, PDB Code 6QR5 and (**F**) **AW7**, PDB Code 6QR8, showing binding mode at the SAM site. The corresponding amino acid interactions are illustrated with polar hydrogen bond interactions and π-interactions in black and purple dotted lines respectively. The TrmD protein is shown as white cartoon model with the interacting amino acid residues in grey lines, the compounds at the active site are represented in light blue stick model with the corresponding Sigma A-weighted m*F*_o_– d*F*_c_ omit maps in grey mesh representation. The 2D chemical structures of each compound and the corresponding affinity (*K*_d_), ligand efficiency (LE) and inhibition data (IC_50_) are also shown in the inset in dark blue.

Structure-guided elaboration of the merged compound **AW1** (*K*_d_ 0.11 mM, LE 0.36, IC_50_ 0.23 mM) was performed, initially utilizing the indole nitrogen as a vector for growth. While the detailed medicinal chemistry strategy of all the compounds and intermediates are described separately (37) and in supporting information (Schemes 1–4 and Supplementary Table S3), in this section we illustrate the key features in the fragment hit to lead optimization of TrmD inhibitors. The addition of a 2-picolyl moiety successfully increased the affinity of **AW1** by an order of magnitude in compound **AW2** (*K*_d_ 12 μM, LE 0.30, IC_50_ 33 μM) (Table 1 and Supplementary Figures S5 and S6). The methylene linker attached to the indole nitrogen of **AW2** allowed the added pyridyl ring to occupy the region defined by Pro85, Glu112, Val137, Arg154 and Glu180 (Figure 4A).

**Table 1.** Summary of structure-guided optimization of merged compound AW1

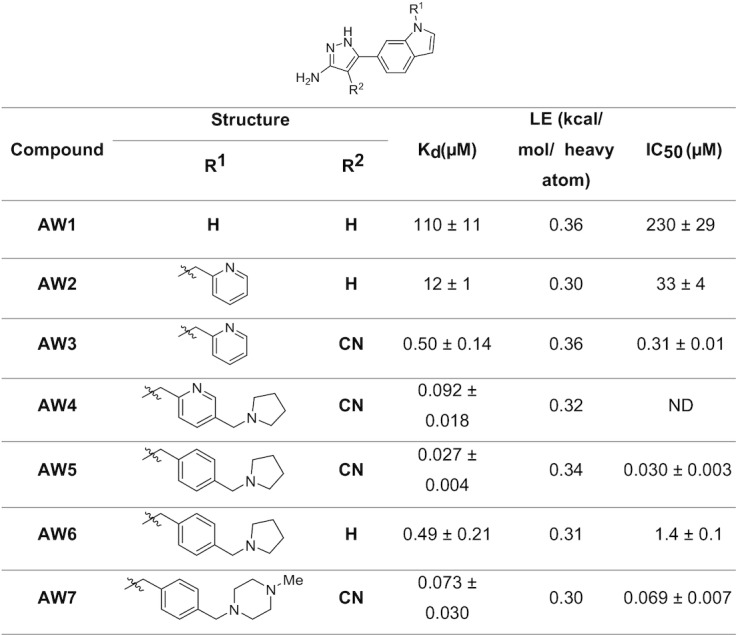

The affinity of **AW2** was further improved by the addition of a nitrile group on the 4-position of its pyrazole ring, extending into the narrow space between residues ^83^PTP^85^ of the cover loop and ^131^VSI^133^ of the bottom loop respectively (Figure 4A, B and Supplementary Figure S4). **AW3** (*K*_d_ 0.50 μM, LE 0.36, IC_50_ 0.31 μM) was a significant improvement on **AW2**, with the addition of two heavy atoms affording a 25-fold decrease in *K*_d_ (12–0.50 μM), increasing the ligand efficiency to the level of the original merged compound **AW1** (0.36), and a 100-fold decrease in IC_50_ (33 to 0.31 μM) (Table 1 and Supplementary Figures S5 and S6). The X-ray crystal structure of TrmD in complex with **AW3** shows that the original fragment contacts have been retained, with the **AW3** aminopyrazole ring orienting itself in a similar manner to **AW1** and retaining its hydrogen bonding contacts to the side chain of Ser132 and backbones of Gly134, Tyr136 and Leu138. In addition, the nitrile group of **AW3** seems to have strengthened the interactions at the active site region between residues ^83^PTP^85^ and ^131^VSI^133^ by engaging in an additional hydrogen bond contact with the backbone NH of Ile133 (Figure 4B).

Further elaboration was carried out from the 5-position of the pyridyl ring of **AW3** through the attachment of a pyrrolidinyl ring via another methylene linker, with **AW4** (*K*_d_ 92 nM, LE 0.34) affording an additional 5-fold improvement in affinity (Table 1 and Supplementary Figures S5 and S7). Modification of the scaffold of **AW4** by replacement of its pyridyl ring with a phenyl ring in **AW5** (*K*_d_ 27 nM, LE 0.34, IC_50_ 30 nM) was tolerated with a >3-fold improvement in affinity (92–27 nM) and an increase in ligand efficiency (0.32–0.34) (Table 1 and Supplementary Figure S5 and S7). The X-ray crystal structure of **AW5** shows the pyrrolidinyl ring occupying the binding site in two conformations, depending on the active site, thereby engaging either Glu112 or Glu180 in an electrostatic interaction (Figure 4C, D and Supplementary Figure S4). The removal of the nitrile group on the pyrazole ring of **AW5** in compound **AW6** (*K*_d_ 0.49 μM, LE 0.31, IC_50_ 1.4 μM) (Table 1 and Supplementary Figures S5 and S7), had a detrimental impact on both affinity and performance in the biochemical assay, demonstrating the importance of extension of this substituent into the cavity between residues ^83^PTP^85^ of the cover loop and ^131^VSI^133^ of the bottom loop (Figure 4E and Supplementary Figure S4). Exploration of the active site region bordered by the Ala176 to Glu180 loop through replacement of the pyrrolidinyl ring of **AW5** with an *N*-methyl piperazinyl motif in **AW7** (*K*_d_ 73 nM, LE 0.30, IC_50_ 69 nM) showed a slight worsening of both affinity and IC_50_ (Table 1 and Supplementary Figures S5 & S8), possibly due to the slight change (0.3 Å) in the position of the nitrile group in comparison to that of **AW5**, thereby diminishing the hydrogen bonding contact with the backbone amide of Thr84 (Figure 4F, Supplementary Figures S4 and S9).

### Anti-mycobacterial activity of TrmD lead compounds

The fragments and developed compounds were then examined for their ability to inhibit bacterial growth. While the initial fragment hits of TrmD and the early stage compounds elaborated from the fragments exhibited low levels of growth inhibition up to 250 μM (data not shown) compounds in later stages of development showed promising activity against *M. abscessus* and *M. tuberculosis* (Table 2). Most of the lead TrmD compounds exhibited much greater inhibition against *M. tuberculosis* than *M. abscessus* (Table 2). Surprisingly, **AW6**, in which the nitrile group of **AW5** was removed, despite being the lesser active lead compound in the *in vitro* TrmD assays_,_ showed similar MICs when compared to the other two compounds. Additionally, the replacement of the pyrrolidinyl ring of **AW5** with an *N*-methyl piperazinyl motif in **AW7** afforded a 2-fold improvement compared to **AW5** in the MIC against *M. tuberculosis*, although not against *M. abscessus* (Table 2). *M. tuberculosis* TrmD *in vitro* binding affinities subsequently determined for **AW6** (*K*_d_ 0.90 μM) and **AW7** (*K*_d_ 0.33 μM) (Supplementary Figure S8) were in keeping with the corresponding MIC values, thereby supporting the applicability of this lead series to TrmD orthologs from mycobacteria other than *M. abscessus*. We therefore tested **AW6** and **AW7** on a wider panel of NTMs including axenically-maintained *M. leprae* and the compounds show inhibitory activity against these bacteria (Table 2 and Supplementary Figure S10). Given the high percentage sequence identity of TrmD across various mycobacterial species (Supplementary Figure S11), the variation in MIC observed for our lead compounds is likely to reflect differential permeability, retention and metabolism of compounds (54,55).

**Table 2.** Minimum inhibitory concentration (MIC) values (μM) of TrmD lead compounds across various mycobacterial species and strains

Compound	*M. abscessus*	*M. tuberculosis*	*M. chelonae*	*M. fortuitum*	*M. gordonae*	*M. terrae*	*M. avium*
**AW5**	50	12.5	ND	ND	ND	ND	ND
**AW6**	50	6.3	100	100	100	100	200
**AW7**	50	6.3	100	100	100	25	100

### Assessment of cytotoxicity and on-target activity of TrmD inhibitors

We assessed the cytotoxicity effect of **AW6** and **AW7** using lactate dehydrogenase (LDH) release assay. It was observed that at or below a concentration of 150 μM, neither compound caused cellular toxicity on human macrophages (Supplementary Figure S12).

**Figure 5.**
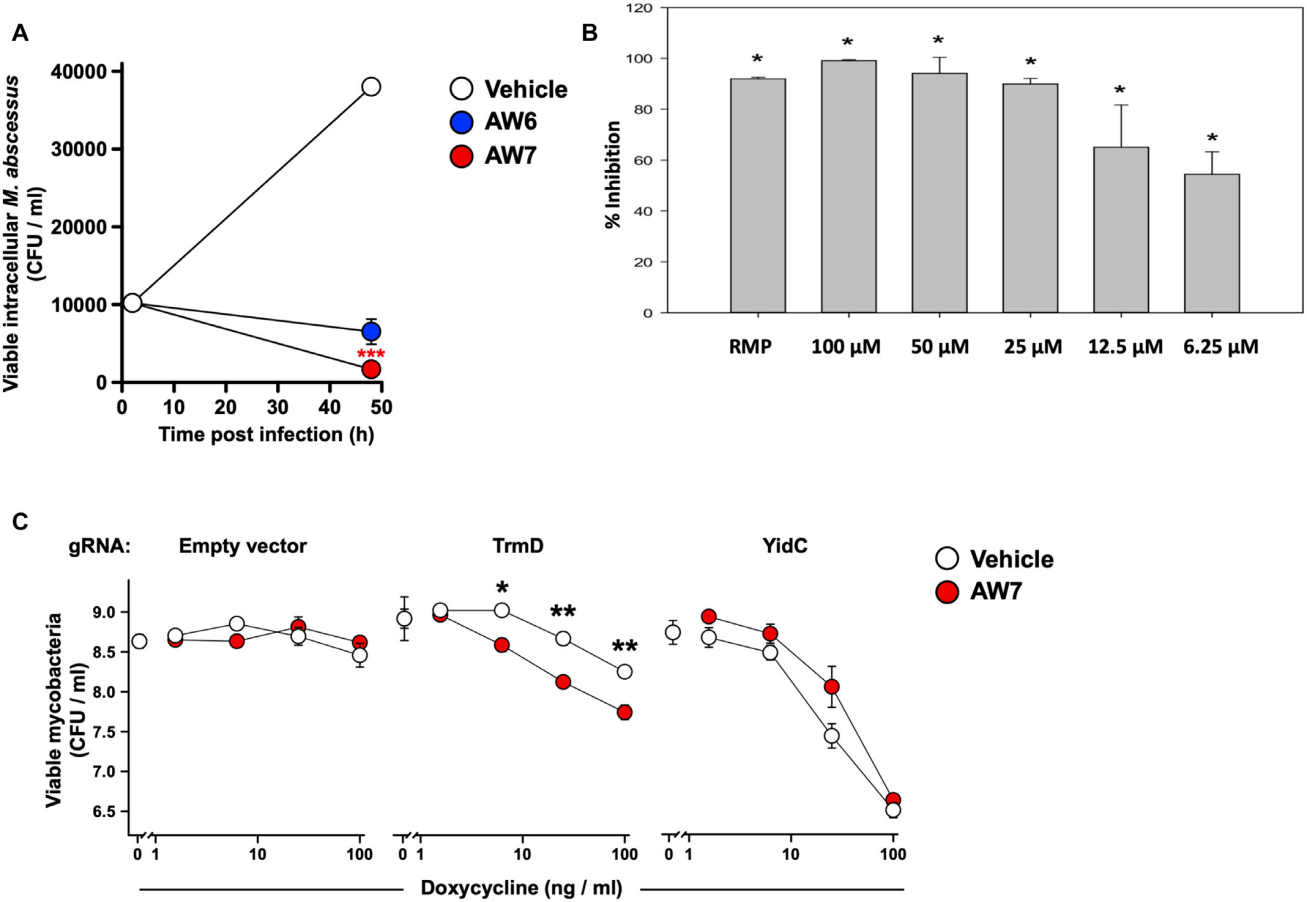
(**A**) Growth inhibition study of lead compounds **AW6** and **AW7** in *M. abscessus* infected human macrophages over a 48 h period. The decrease in CFU/ml following 48 h with 25μM **AW7** is statistically significant (*P* < 0.001) showing the cidality of **AW7** for intracellular *M. abscessus*. (**B**) Intracellular *M. leprae* palmitic acid oxidation rate (radiorespirometry) in the presence of different concentrations of **AW7** for 7 days. Seventh day cumulative counts per minute (CPM) were recorded and percentage inhibition of metabolism determined as compared to no drug control. **AW7** concentrations, in mM, are shown in parenthesis and rifampin (RMP) was used at 2.4 μM. (**C**) Target engagement of **AW7** with *M. abscessus* TrmD. Incremental silencing of *trmD* using a CRISPR dCas9 tunable system further sensitises *M. abscessus* to **AW7** while such effect is not observed for the empty vector or for another essential gene *yidC*.

Further, to determine whether the anti-mycobacterial activity of our compounds was directly related to TrmD inhibition and not caused by an off- target effect, we generated a Tet- inducible CRISPR-dCas9 system in *M. abscessus*, to tunably knockdown selected protein expression. Incremental silencing of TrmD, achieved through exposure to increasing concentrations of doxycycline, resulted in *M. abscessus* to become progressively more sensitive to **AW7**. Further, this effect was not seen in bacteria expressing gRNA targeting another essential gene (*yidC*) or an empty vector control (Figure 5C), thereby confirming target engagement

### TrmD inhibitors kill intracellular *M. abscessus* and *M. leprae*

The compounds were then evaluated in *M. abscessus*-infected human macrophages. Both compounds showed inhibitory activity in the macrophage infection model, with **AW7** performing better than **AW6** in the *in vitro* assays. At 25 μM **AW6** showed a ∼82% decrease in CFUs while **AW7** at the same concentration showed a 95% reduction in CFUs compared to the no drug control after 48h incubation (Figure 5A).

The best lead molecule (**AW7**) was further tested against *M. leprae* maintained intracellularly in murine bone marrow macrophage. As a proxy for mycobacterial viability, relative inhibition of β-oxidation rates were measured using a radiorespirometry assay to track the metabolism of ^14^C-palmitic acid to ^14^CO_2_ after 7 days of incubation (45). We found that **AW7** had considerable inhibitory effect against *M. leprae*, being able to inhibit β-oxidation by over 50% at a concentration as low as 6.2 μM (Figure 5B).

## DISCUSSION

Most current antibiotics targeting protein synthesis act either by interacting with ribosomal sub-units (for example aminoglycosides, tetracyclines, and macrolides) or *via* inhibiting mRNA synthesis (rifamycins) and elongation (actinomycin) (56), to which many clinically relevant bacteria have developed resistance. However, other unexplored mechanisms exist to disrupt protein synthesis, including by promoting frame-shift translational errors. We have focused on targeting TrmD (tRNA-(N(1)G37) methyltransferase), an essential tRNA modification enzyme in bacteria that methylates the guanosine base at position 37 of tRNAs containing G_36_G_37_ bases at the anti-codon region to prevent frame-shifting. Inhibition of TrmD will therefore promote defective protein synthesis leading to cell death. Until now, very few inhibitors have been developed against it and those exhibit generally low bactericidal activity. Our study provides proof of concept that a fragment-based approach can be successfully used to target mycobacterial tRNA modification, delivering a new class of antibiotics with potent bactericidal activities.

Fragments are powerful chemical tools that have huge capacity to explore the chemical space of proteins (57) with several drugs developed through the use of FBDD recently approved to treat cancer. Our work demonstrates the potential of this technique to create new antibiotics, with careful structure-guided optimisation of initial fragment hits rapidly producing potent inhibitors with anti-mycobacterial activity. Furthermore, our fragment screening revealed numerous other hits with diverse binding modes, offering multiple possibilities for future compound elaboration and optimization.

Mycobacteria are a notoriously difficult group of microorganisms to develop new drugs against, due to their impermeable cell wall, efflux pumps, target modification enzymes, and extensive capacity to metabolise compounds (58). TrmD is a highly conserved enzyme in mycobacteria (Supplementary Figure S11) and the *M. abscessus* TrmD compounds developed in this study, not only showed potent affinity toward *M. tuberculosis* TrmD but also were active against several other mycobacteria, including *M. leprae*. While we succeeded in developing potent TrmD inhibitors, the disconnect observed between compound affinity *in vitro* and bacterial MIC for some of these molecules, will require further iterative efforts to optimize permeability, retention and/or intra-bacterial metabolic stability of the compounds. It will be also interesting to probe the effect of the TrmD inhibitors developed in this study on membrane protein and efflux pump biosynthesis in mycobacteria, as previously observed in gram-negative bacteria (15). Such an effect, if also present in mycobacteria, could indicate an additional role of TrmD inhibitors by way of sensitizing mycobacteria towards antibiotics. Nevertheless, the results of this study, with macrophage infection models, the demonstrated on-target activity and the encouraging preliminary cytotoxicity data, shows the potential of these molecules to be further developed into novel mycobacterial drugs.

## DATA AVAILABILITY

Coordinates and structure factors related to this work been deposited in the PDB with accession numbers 6NVR, 6NW6, 6NW7, 6QO2, 6QO3,6QO4, 6QO6, 6QOA, 6QOC, 6QOD, 6QOE, 6QOF, 6QOG, 6QOH, 6QOI, 6QOJ, 6QOK, 6QOL, 6QOM, 6QON, 6QOO, 6QOP, 6QOQ, 6QOR, 6QOS, 6QOT, 6QOU, 6QOV, 6QOW, 6QOX, 6QQS, 6QQX, 6QQY, 6QR6, 6QR5 and 6QR8.

## Supplementary Material

gkaa539_Supplemental_File
