# Supplementary material for: Fragment-based discovery of a new class of inhibitors targeting mycobacterial tRNA modification
Source: Nucleic Acids Res. 2020 Jun 30;48(14):8099–112. doi: 10.1093/nar/gkaa539 (PMC7641325; doi:10.1093/nar/gkaa539)
Supplement: gkaa539_Supplemental_File [file gkaa539_supplemental_file.pdf]

Supplementary Data

Fragment-based discovery of a new class of inhibitors targeting mycobacterial tRNA modification

Sherine E. Thomas[1], Andrew J. Whitehouse[2], Karen Brown[3,4], Sophie Burbaud[3], Juan M. Belardinelli[5], Jasper Sangen[3], Ramanuj Lahiri[6], M. Daben J. Libardo[7], Pooja Gupta[1], Sony Malhotra[8], Helena I. M. Boshoff[7], Mary Jackson[5], Chris Abell [2], Anthony G. Coyne[2], Tom L. Blundell [*1], R. Andres Floto[*3,4], Vítor Mendes [*1]

[1] Department of Biochemistry, University of Cambridge, 80 Tennis Court Road, Cambridge, CB2 1GA, UK

[2] Department of Chemistry, University of Cambridge, Lensfield Road, Cambridge, CB2 1EW, UK.

[3] University of Cambridge Molecular Immunity Unit, MRC Laboratory of Molecular Biology, Francis Crick Avenue, Cambridge, CB2 0QH, UK.

[4] Cambridge Centre for Lung Infection, Royal Papworth Hospital, Cambridge, CB23 3RE, UK.

[5] Mycobacteria Research Laboratories, Department of Microbiology, Immunology and Pathology, Colorado State University, Fort Collins, Colorado, USA.

[6] National Hansen's Disease Program, Healthcare Systems Bureau, Health Resources and Services Administration, Department of Health and Human Services, Baton Rouge, Louisiana, USA.

[7] Tuberculosis Research Section, Laboratory of Clinical Immunology and Microbiology, National Institute of Allergy and Infectious Disease, National Institutes of Health, 9000 Rockville Pike, Bethesda, Maryland 20892, USA.

[8] Birkbeck College, University of London, Malet Street, WC1E7HX, UK

[*] To whom correspondence may be addressed.

Vitor Mendes: vgm23@cam.ac.uk

Andres Floto: arf27@cam.ac.uk

Tom Blundell: tom@cryst.bioc.cam.ac.uk

Expression and purification of *M. tuberculosis* TrmD

A colony of *E. coli* strain ANG3685 (XL1 Blue pET23b-His6-trmDTB) kindly provided by the research group of Professor Angelika Gründling at Imperial College London (1), was transferred to LB media (5 mL) with ampicillin (100 μg mL^{-1}) and incubated overnight (37 °C, 160 rpm). The resultant material was processed with a GeneJET™ Plasmid Miniprep Kit (Thermo Scientific™) to obtain plasmid (30 ng μL^{-1}, A_{260nm}/A_{280nm} 1.87), with identity confirmed by Sanger sequencing (DNA Sequencing Facility, Department of Biochemistry, University of Cambridge).

The isolated plasmid was used to transform *E. coli* strain BL21 (DE3), with a colony transferred to LB media (20 mL) with ampicillin (100 μg mL^{-1}) and incubated overnight (37 °C, 160 rpm). The starter culture was used to inoculate 2 flasks, each containing LB media (1 L) with ampicillin (100 μg mL^{-1}), with incubation (37 °C, 200 rpm) until an optical density (A_{600nm}) of 0.5 was reached. Protein expression was induced by the addition of isopropyl β-D-1-thiogalactopyranoside (0.5 mM), followed by overnight incubation (20 °C, 200 rpm). Cells were harvested by centrifugation (4 °C, 4000 g, 20 minutes), then frozen.

The cells were resuspended in 50 mL lysis buffer (50 mM HEPES pH 7.4, 1 M NaCl, 25 mM imidazole, 5 mM mercaptoethanol) with a tablet of cOmplete™ Protease Inhibitor Cocktail (Roche). The suspension was sonicated (10 minutes: 10 seconds on/ 20 seconds off), centrifuged (4 °C, 30000 g, 20 minutes) and filtered (0.45 μm). The resultant lysate was loaded onto a 7.5 mL nickel Sepharose™ fast flow column (GE Healthcare), pre-equilibrated with lysis buffer. The column was washed with 5 column volumes of buffer A (25 mM HEPES pH 7.5, 500 mM NaCl, 20 mM imidazole, 5 mM mercaptoethanol) and eluted with buffer B (25 mM HEPES pH 7.5, 500 mM NaCl, 500 mM imidazole, 5 mM mercaptoethanol) in 7 x 5 mL aliquots. Protein-containing aliquots, as determined by SDS-PAGE, were combined and concentrated (10 kDa cutoff) to a volume of 7 mL, then loaded onto a Superdex 75 Hiload™ 16/60 column (GE Healthcare) pre-equilibrated with filtration buffer (25 mM HEPES pH 7.5, 500 mM NaCl, 5 mM mercaptoethanol). Protein-containing fractions, as determined by SDS-PAGE, were combined and concentrated (10 kDa cutoff) to 14.4 mg mL^{-1} (5.0 mg L^{-1} yield), then flash-frozen in liquid nitrogen and stored at -80 °C. The identity of the protein was confirmed by LCMS analysis.

Allelic replacement studies

Table S1: Primer sequences used in the allelic replacement experiments on *M. abscessus* subsp. *massiliense* CIP108297

**Primer name**	**Sequence**
trmD Up Fw	5'-CCTGCATATGGCCGAGGGCACCTTGAAGGTC-3'
trmD Up Rv	5'-CCGGGAATTCGGGAAGATCGTGACGACGTCTAT-3'
trmD Dn Fw	5'-GGCCGAATTCTCGCGTCAGCGCACTGCTGAG-3'
trmD Dn Rv	5'-AAGTACTAGTAGGCTACCGTGACCGGAAC-3'
trmD KO Fw	5'-CGGTGCTGGCGCTGCTGAAGA-3'
trmD KO Rv	5'-GCACCTCAGCCGATCATTAGTTG-3'

Results

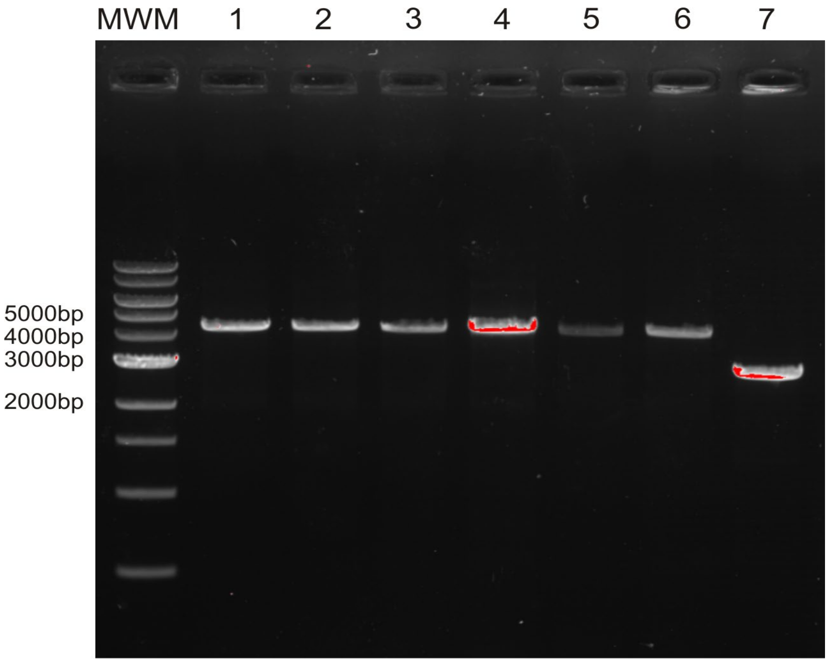

Figure S1: Allelic replacement at the *trmD* locus of *M. abscessus*. Candidate *trmD* mutants were analysed by PCR using a set of primers annealing outside the allelic exchange substrate. Lanes 1 to 6 show *M. abscessus subsp. massiliense* CIP108297 clones harboring the pMV306H::*trmD* plasmid whose endogenous chromosomal *trmD* locus was replaced by the Str cassette (expected size of the PCR fragment is 4,200 bp). Lane 7 shows the amplification product of the *trmD* locus in wild-type *M. abscessus subsp. massiliense* CIP108297 (expected size of the PCR fragment is 2,571 bp). MWM, molecular weight marker.

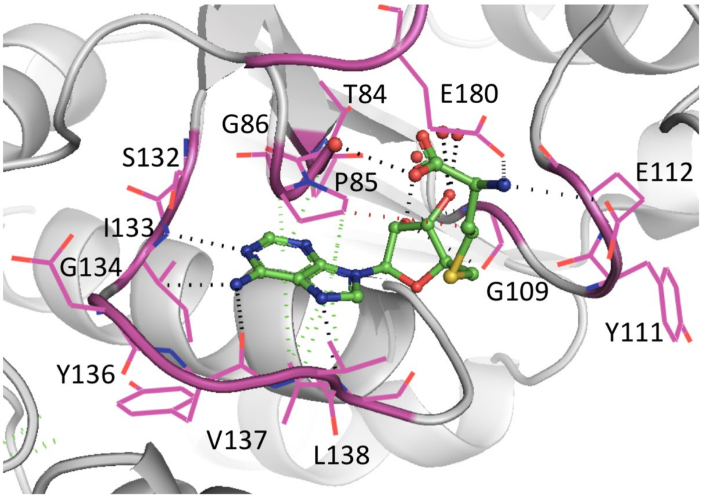

Figure S2: Interaction map of TrmD (pink) in complex S-adenosyl homocysteine (**SAH**), PDB code **6NW7**, shown as green stick representation. Hydrogen bonds, hydrophobic contacts, π-interactions are depicted in black, green and red dotted lines respectively

X-ray crystal structures and clustering for all fragment hits reported in this work

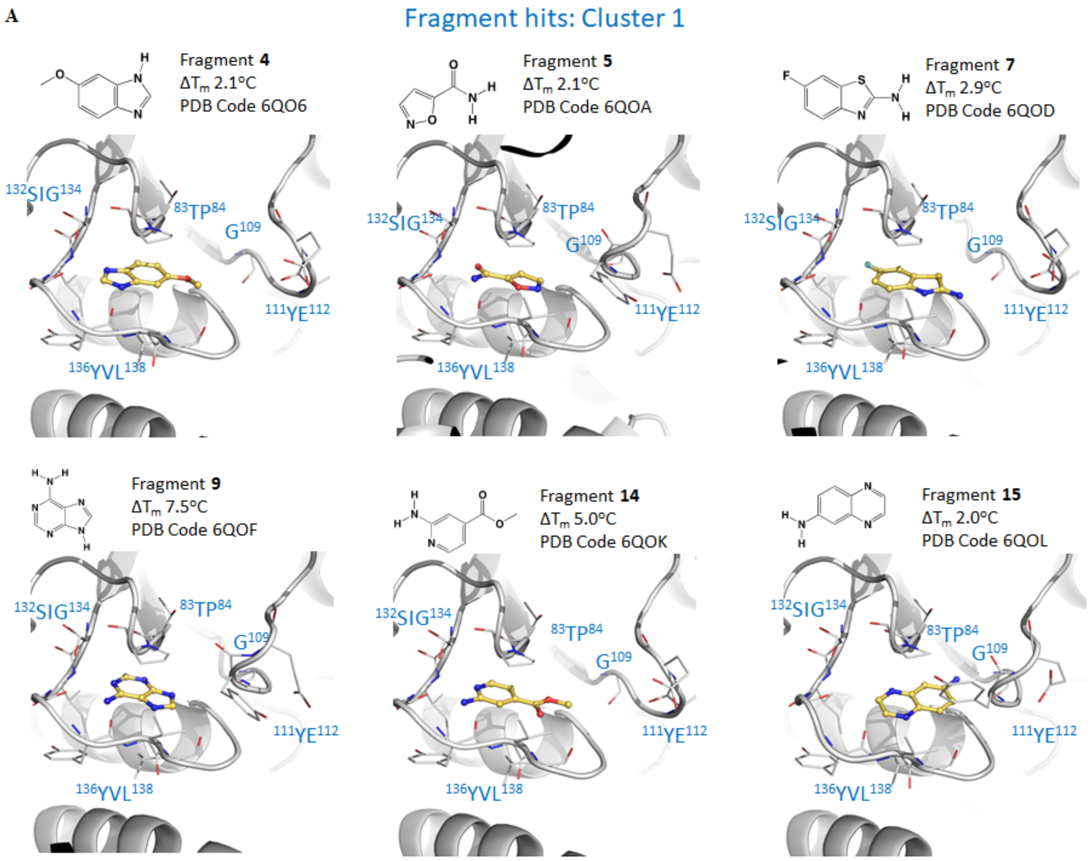

B

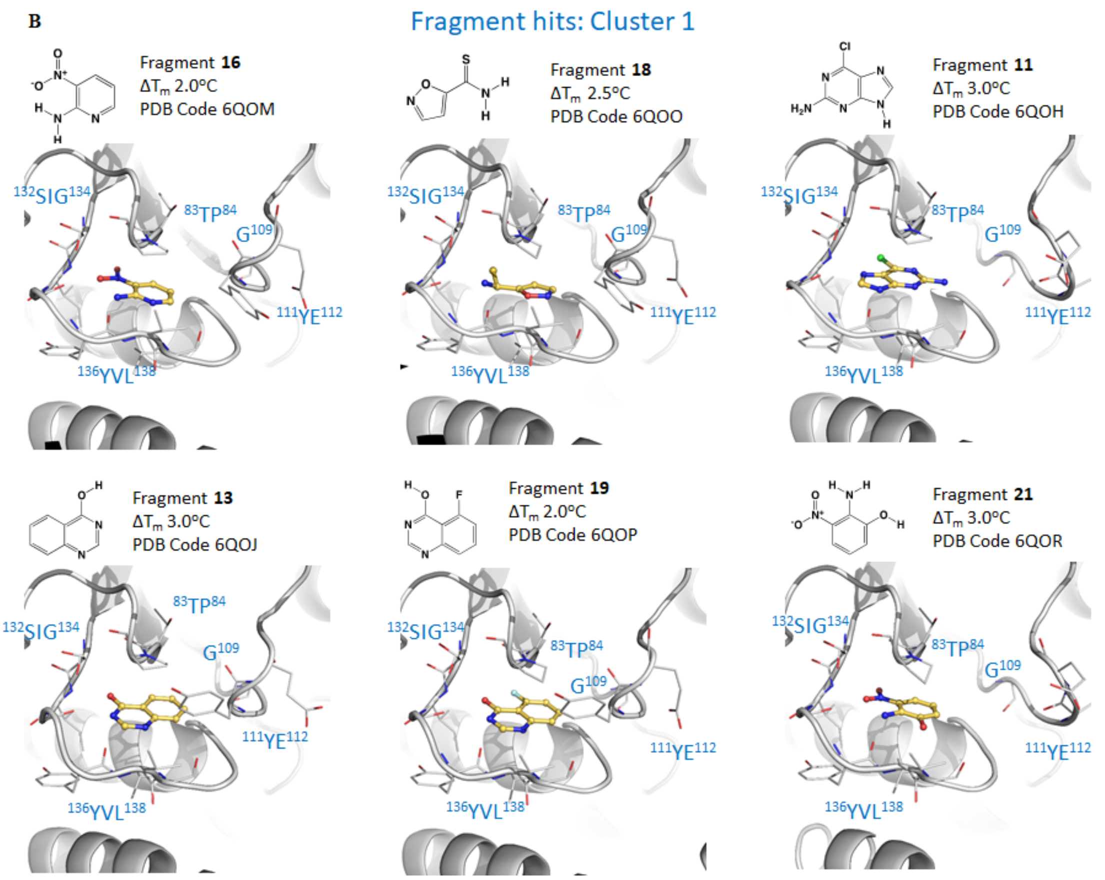

Fragment hits: Cluster 1
Fragment **16**
ΔT_m 2.0°C
PDB Code 6QOM
Fragment **18**
ΔT_m 2.5°C
PDB Code 6QOO
Fragment **11**
ΔT_m 3.0°C
PDB Code 6QOH
Fragment **13**
ΔT_m 3.0°C
PDB Code 6QOJ
Fragment **19**
ΔT_m 2.0°C
PDB Code 6QOP
Fragment **21**
ΔT_m 3.0°C
PDB Code 6QOR

C

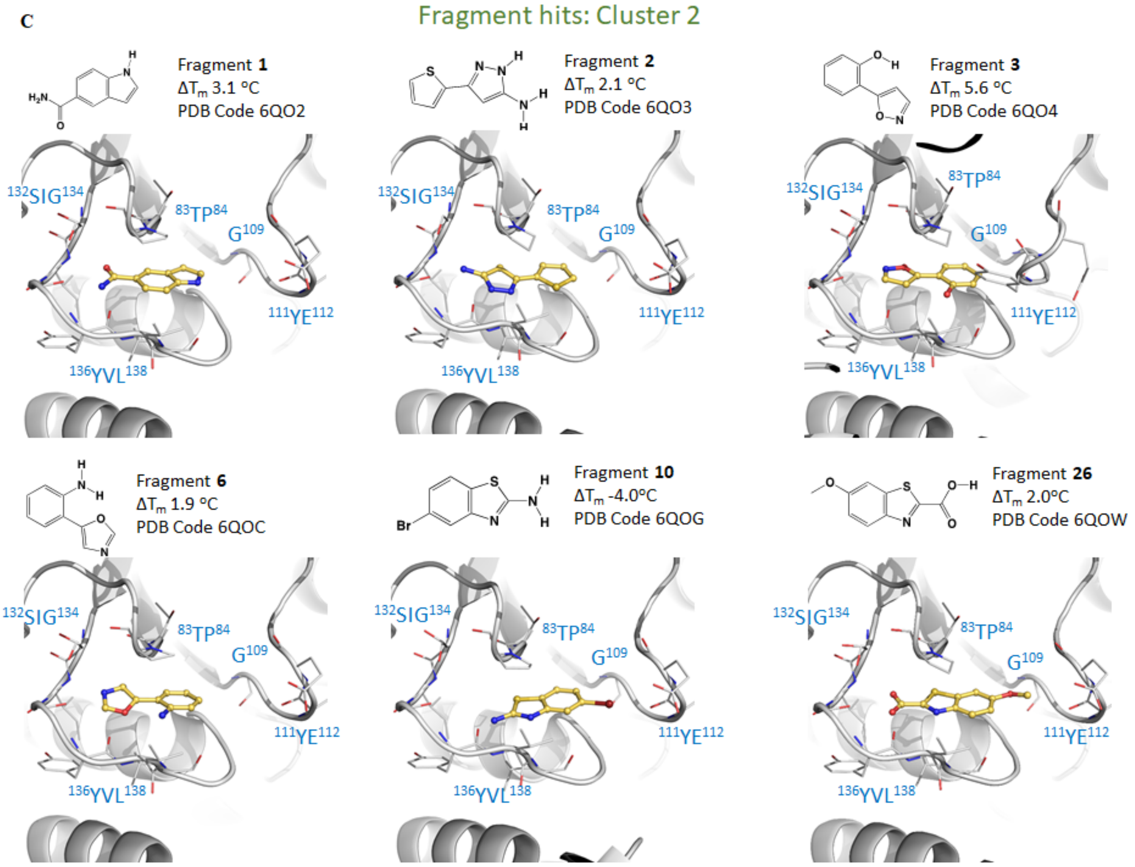

Fragment hits: Cluster 2
Fragment **1**
ΔT_m 3.1 °C
PDB Code 6QO2
Fragment **2**
ΔT_m 2.1 °C
PDB Code 6QO3
Fragment **3**
ΔT_m 5.6 °C
PDB Code 6QO4
Fragment **6**
ΔT_m 1.9 °C
PDB Code 6QOC
Fragment **10**
ΔT_m -4.0°C
PDB Code 6QOG
Fragment **26**
ΔT_m 2.0°C
PDB Code 6QOW

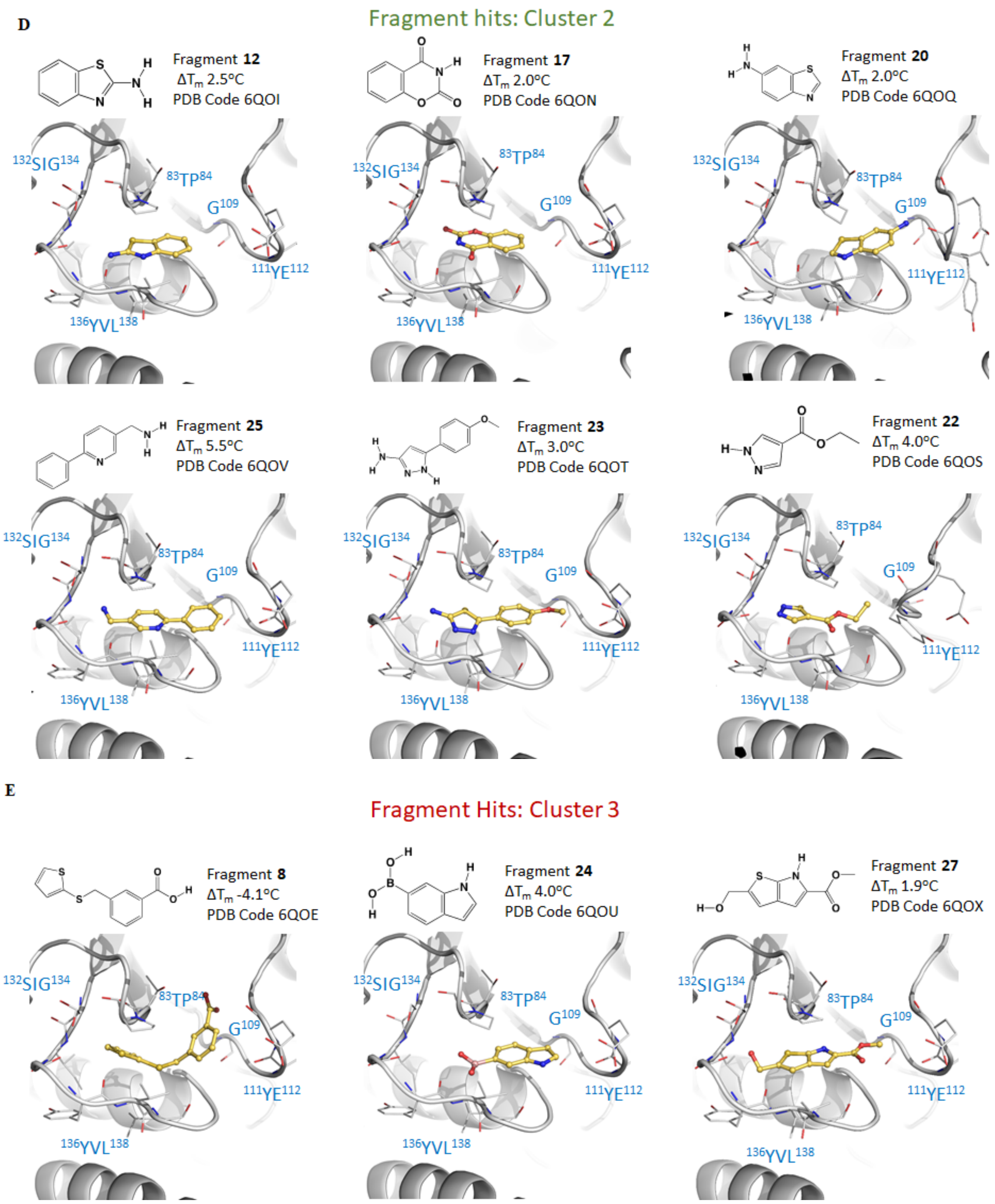

Figure S3 A-E: Hits obtained from fragment library screening of TrmD. Fragment hits identified from DSF and X-ray crystallography, clustered into three groups based on the mode of binding at the M. abscessus TrmD active site.

Difference electron density maps (omit maps) for ligands illustrated in this study

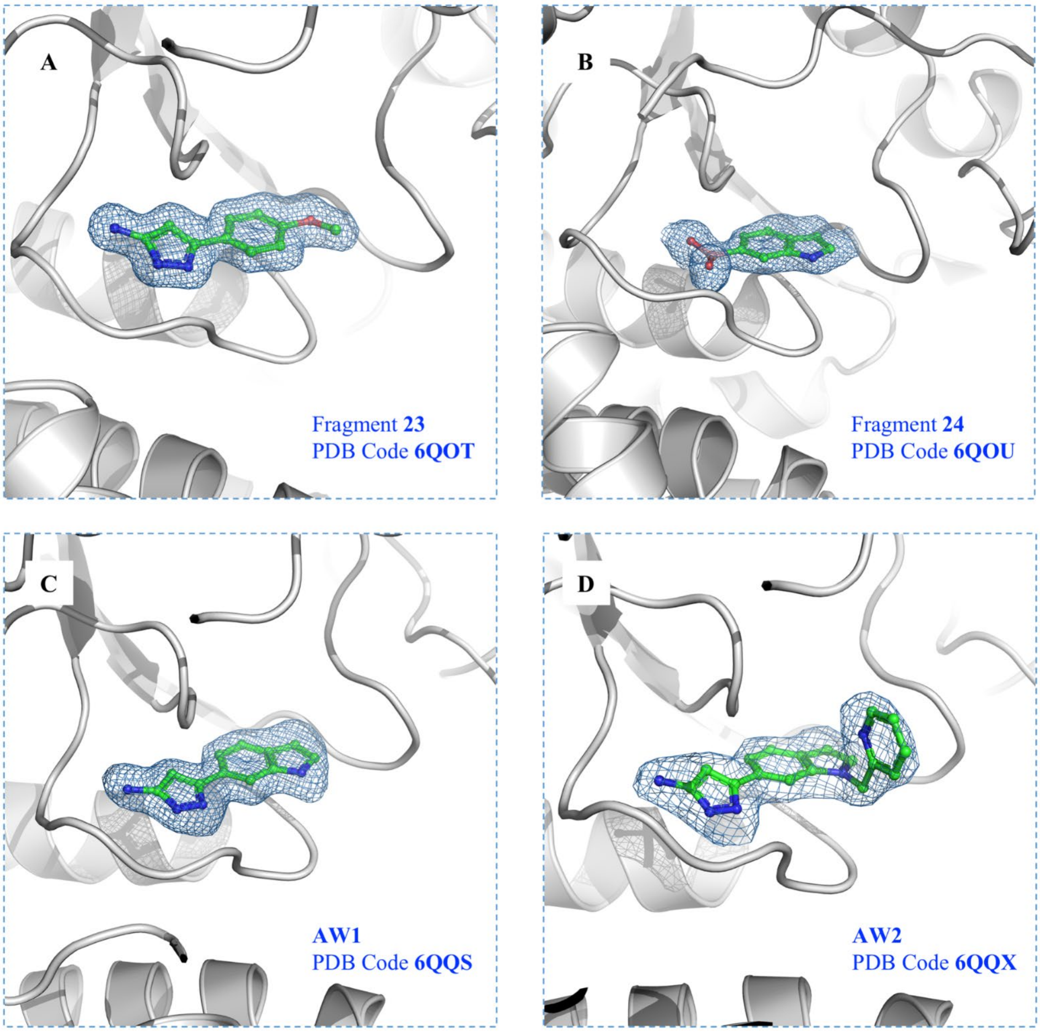

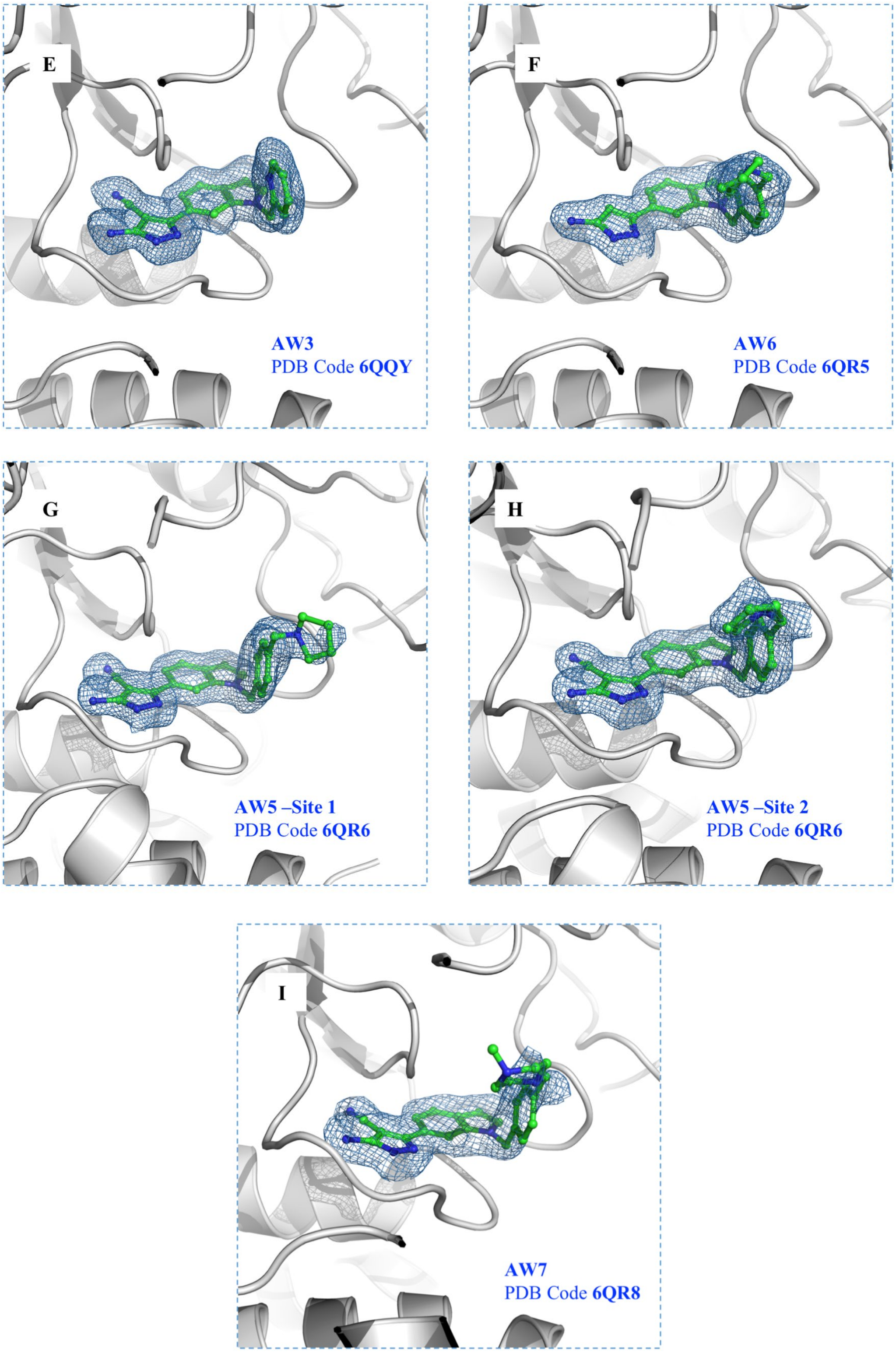

Figure S4: Fo-Fc Omit maps corresponding to fragments and compounds described in this study

Enzymatic assays

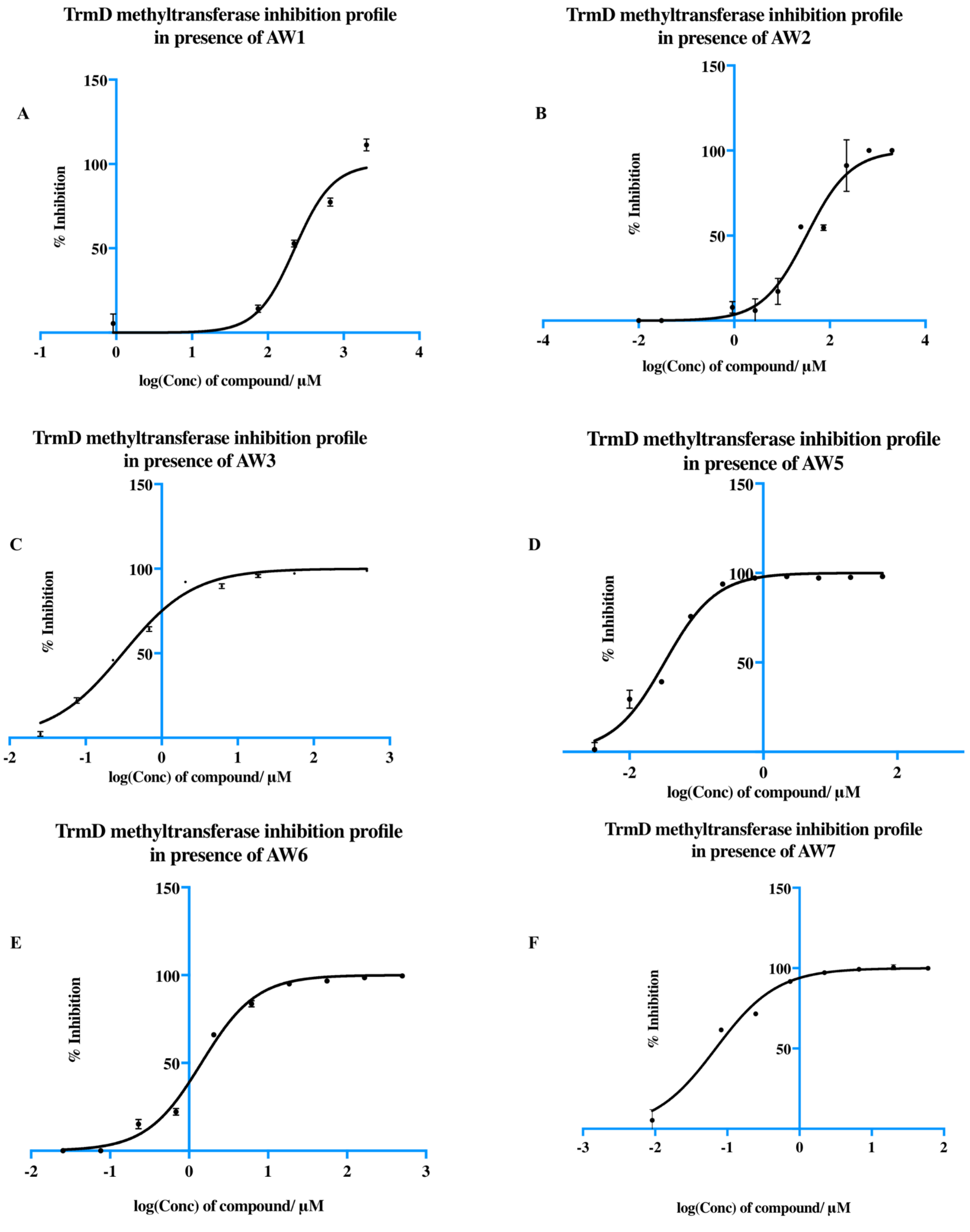

Figure S5A-F: *M. abscessus* TrmD Methyltransferase inhibition profiles in the presence of TrmD lead compounds AW1-7.

Isothermal Titration Calorimetry (ITC)

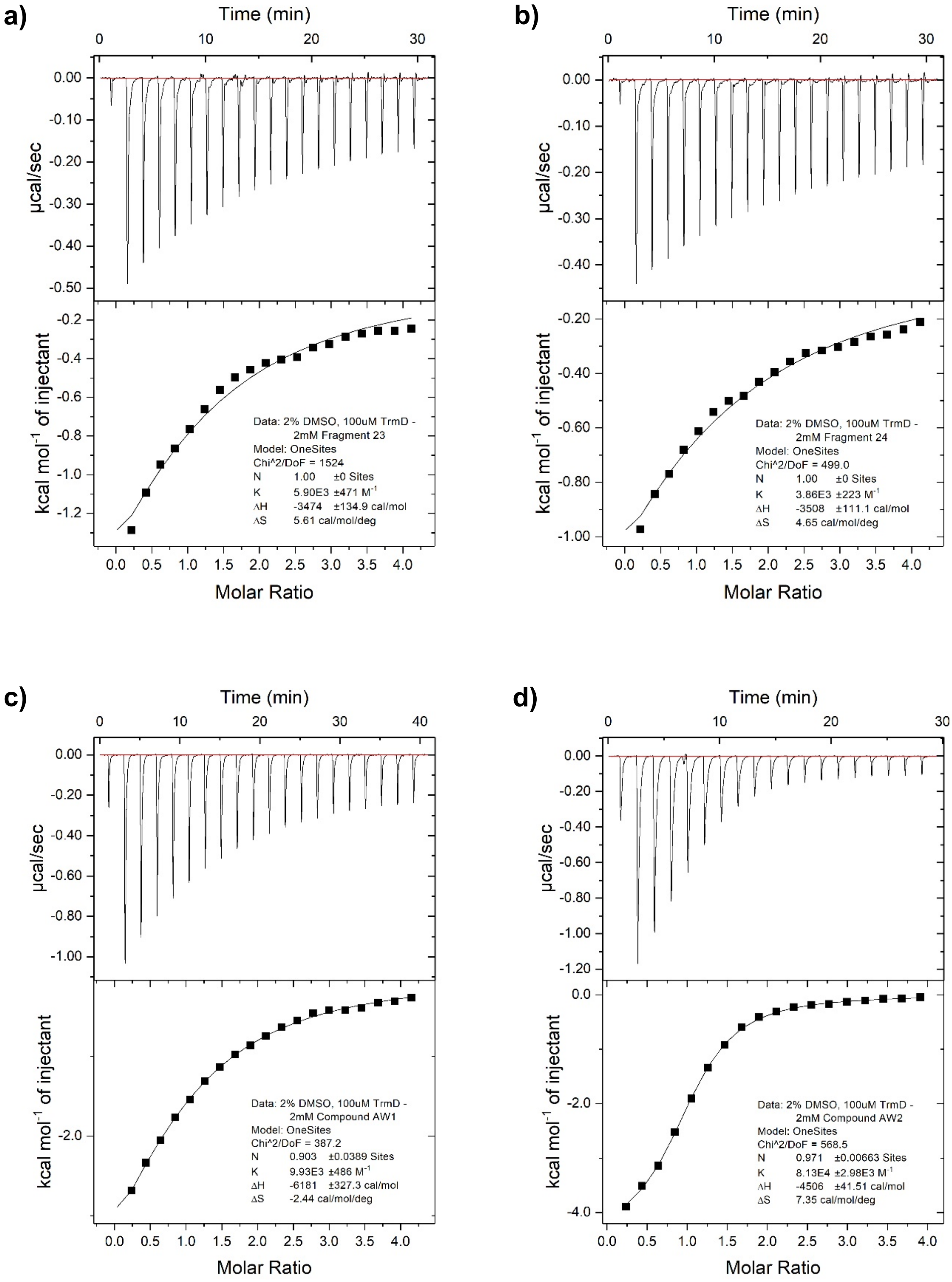

Figure S6: ITC traces with TrmD from Mycobacterium abscessus for a) fragment 23 (n = 1), b) fragment 24 (n = 1), c) AW1 (n = 2), and d) AW2 (n = 1).

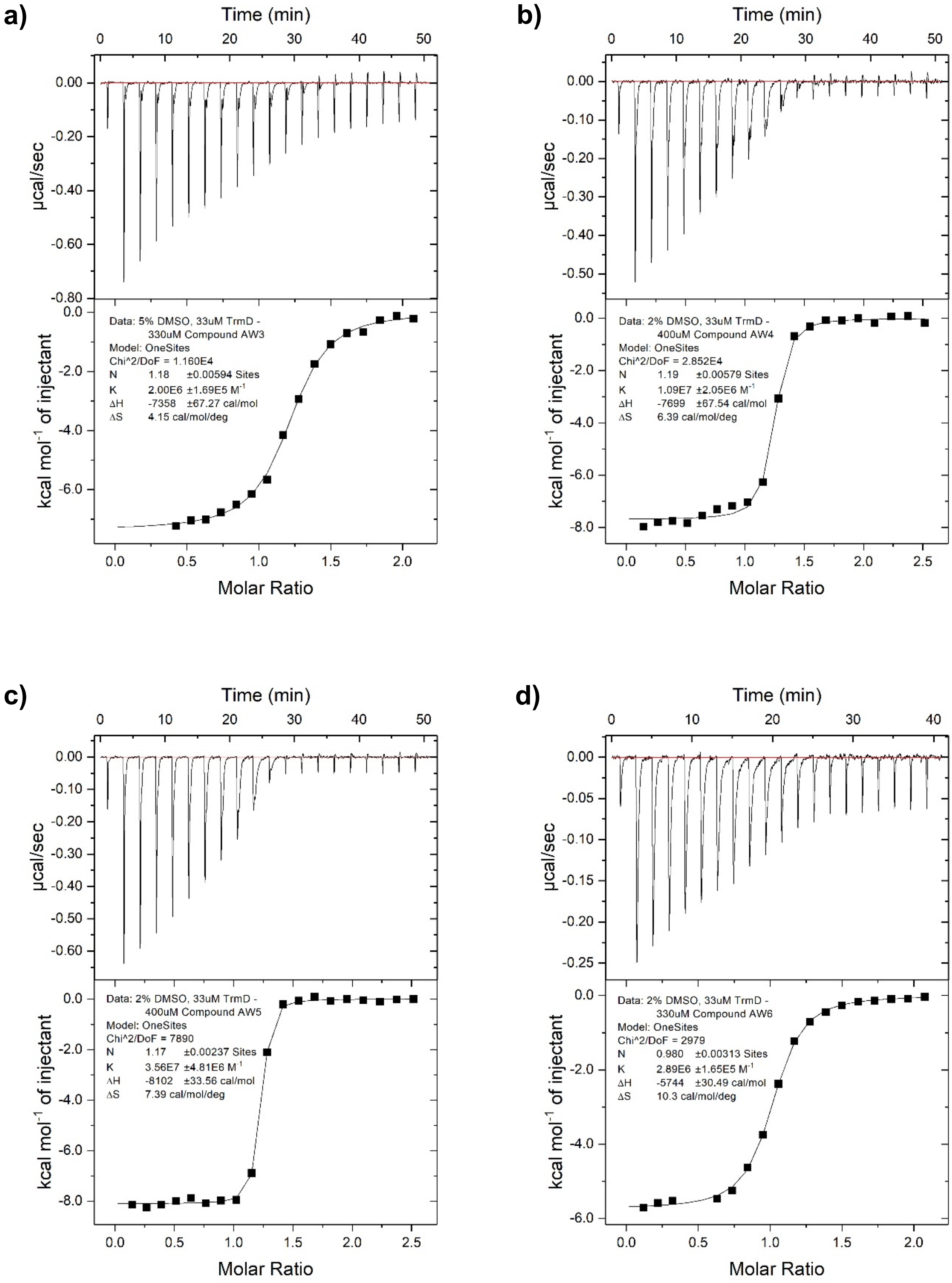

Figure S7: ITC traces with TrmD from Mycobacterium abscessus for a) AW3 (n = 3), b) AW4 (n = 1), c) AW5 (n = 3), and d) AW6 (n = 2).

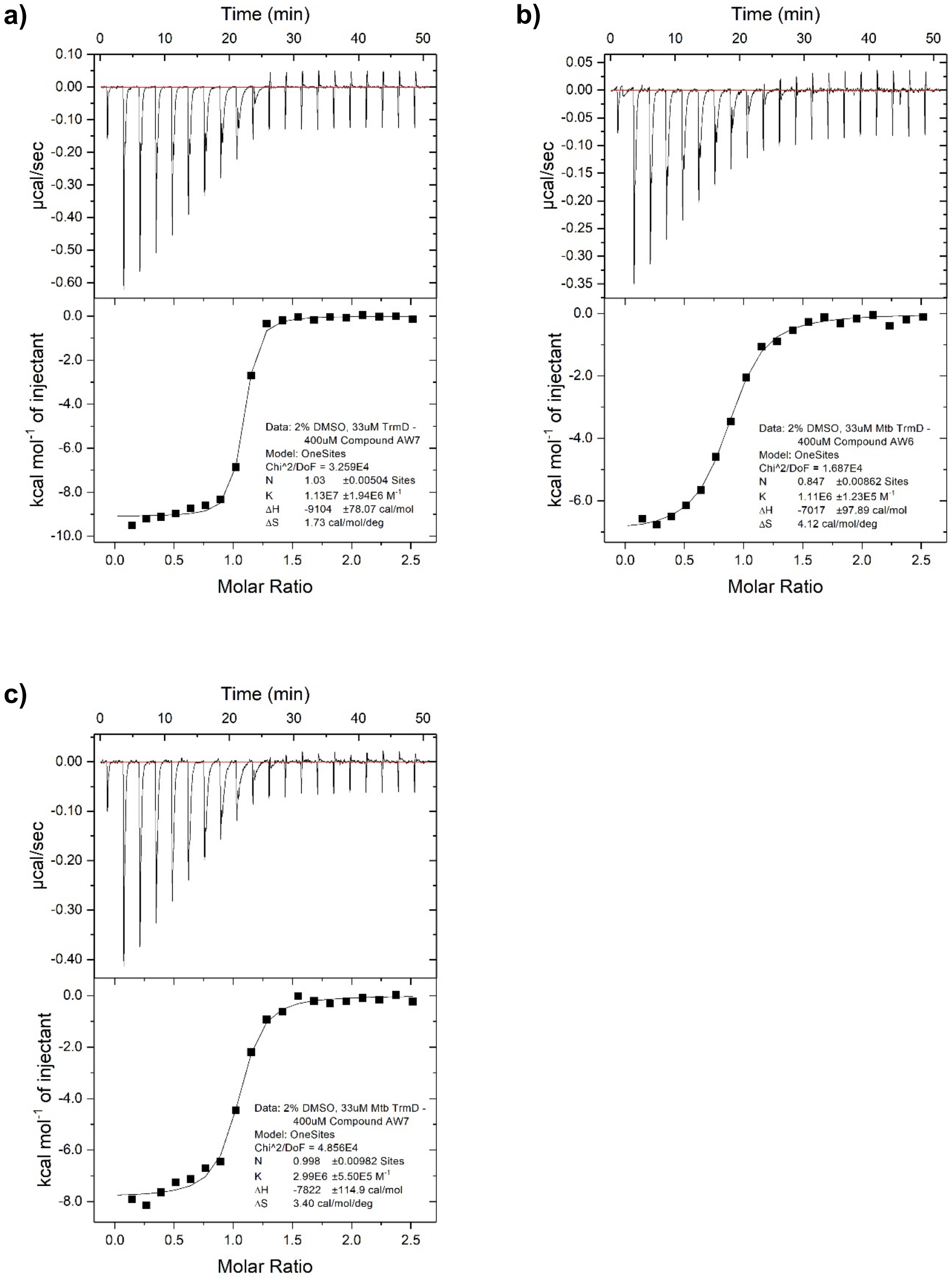

Figure S8: ITC trace with TrmD from Mycobacterium abscessus for a) AW7 (n = 3), and ITC traces with TrmD from Mycobacterium tuberculosis (n = 1) for b) AW6, and c) AW7.

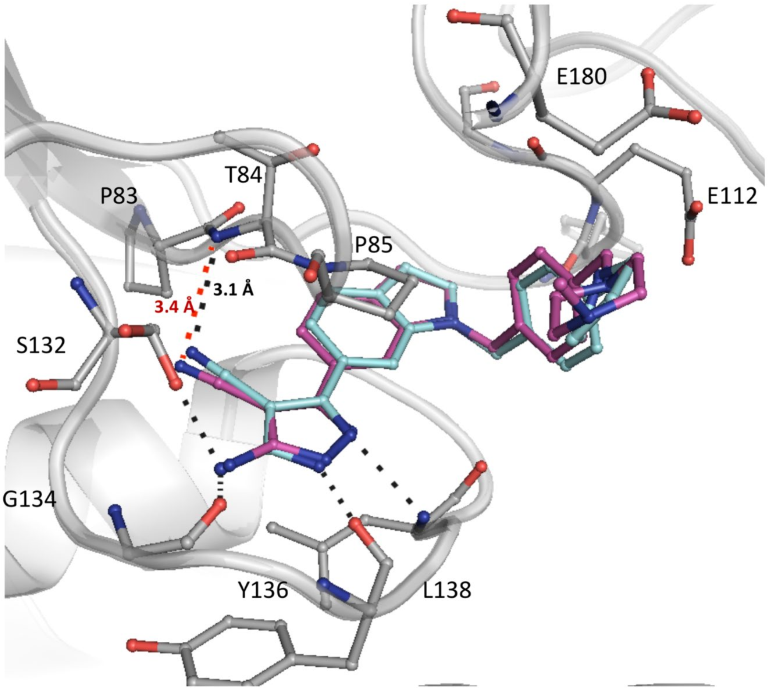

Figure S9: Superposition of the crystal structures of TrmD in complex with AW5 (blue stick) and AW7 (pink stick) showing changes in positon of the nitrile group leading to increased distance of 3.4 Å from Thr84 backbone amide there by diminishing H-bond interaction as compared to AW5 (3.1 Å) nitrile.

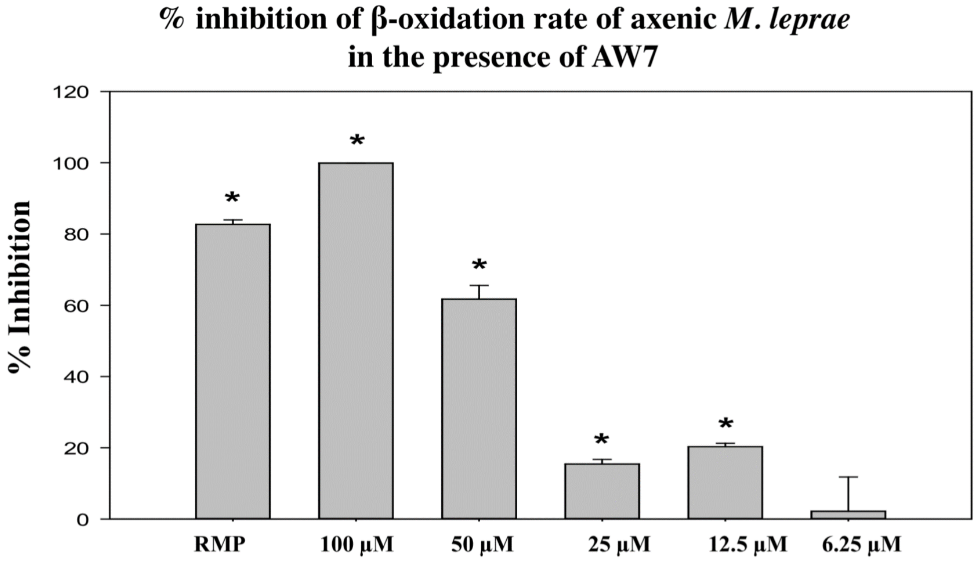

Figure S10: Percentage inhibition of M. leprae palmitic acid oxidation rate (radiorespirometry) in the presence of different concentrations of AW7 for 7 days. 7th day cumulative counts per minute (CPM) were recorded and percentage inhibition of metabolism determined as compared to no drug control. AW7 concentrations, in mM, are shown in parenthesis and rifampin (RMP) was used at 2mg/mL. * - Inhibition is statistically significant ($P<0.05$) compared to no drug control. Data representative of three separate experiments.

Conservation of catalytic residues in mycobacterial TrmD

A multiple sequence alignment of 5 mycobacterial TrmD enzyme sequences with 10 TrmD ortholog amino acid sequences (Figure S1) shows conservation of important catalytic residues and residues involved in tRNA recognition and methyl transfer reactions. The catalytic residues Asp169 and Arg154 are highly conserved across TrmD orthologs. Key residues such as Asp50, Gly59, His46 involved in tRNA G36 and G37 base recognition, interactions with bases at positions 38, 32 and anticodon branch of wild type tRNA respectively, are also broadly conserved.

In mycobacterial TrmD, a Histidine replaces Phe171 which participates in recognition by the AdoMet methionine moiety initiating the catalytic cascade leading to methyl transfer reaction (2). Further, the C-terminal motif S-G-H/D-H involved in tRNA minor groove recognition is highly conserved across TrmD orthologs. The amino-acid sequence of *M. abscessus* TrmD (MAB-3226c) was obtained from the National Centre for Biotechnology Information (NCBI) database. Orthologous proteins from *M.abscessus ; M. chelonae; M. fortuitumM.terrae, M.leprae; M.gordonae; M.tuberculosis; M.avium; Pseudomonas aeruginosa; Haemophilus influenzae; Escherichia coli* were identified by performing a protein-BLAST (3,4) search with *M. abscessus* protein sequence. The sequences were aligned using Clustal Omega (5) and analysed using Jalview (6).

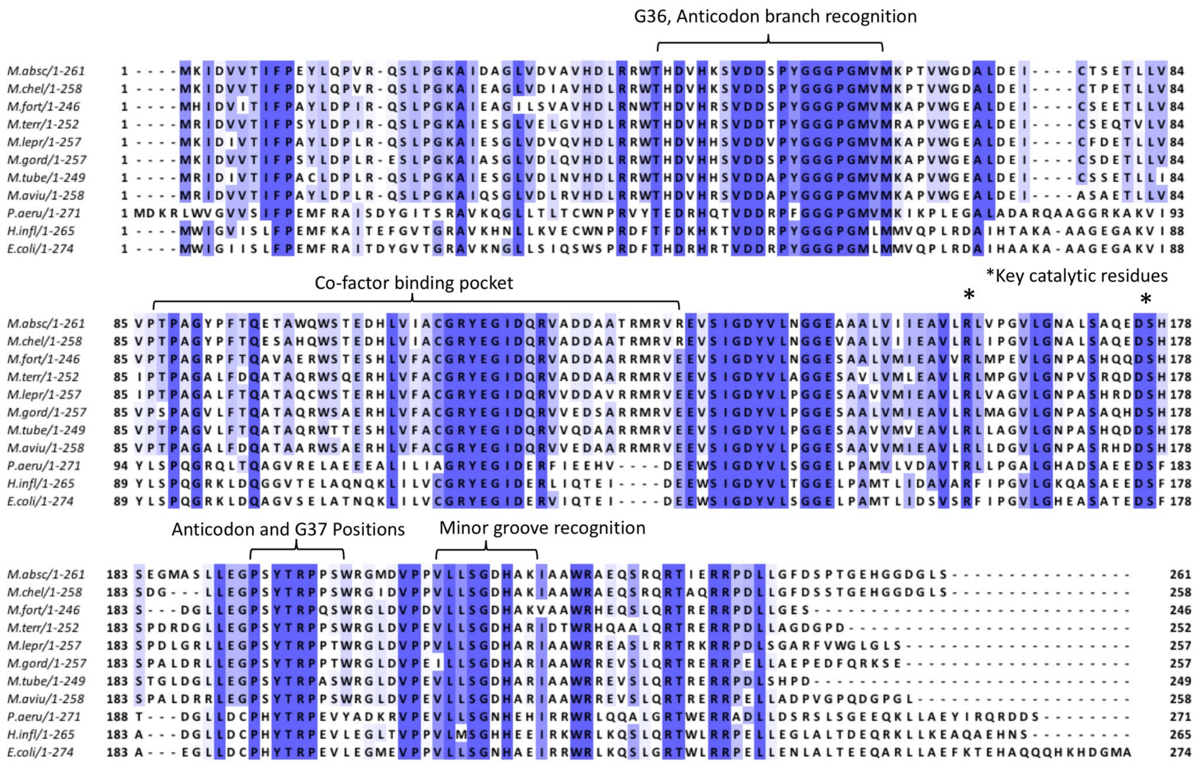

Figure S11: Multiple sequence alignment of 8 Mycobacterial TrmDs (M.absc - *M. abscessus*; M.chel - *M. chelonae*; M.fort - *M. fortuitum*; M. terr - *M. terrae*; M.lepr - *M. leprae*; M. gord - *M. gordonae*; M.tube - *M. tuberculosis*; M.aviu - *M. avium*) with 3 other bacterial TrmD orthologs (P.aeru - *Pseudomonas aeruginosa*; H.influ - *Haemophilus influenzae*; E.coli - *Escherichia coli*) coloured from white to dark purple indicating increasing percentage conservation of amino acid residues. The important catalytic residues and their corresponding functions are also illustrated in the alignment.

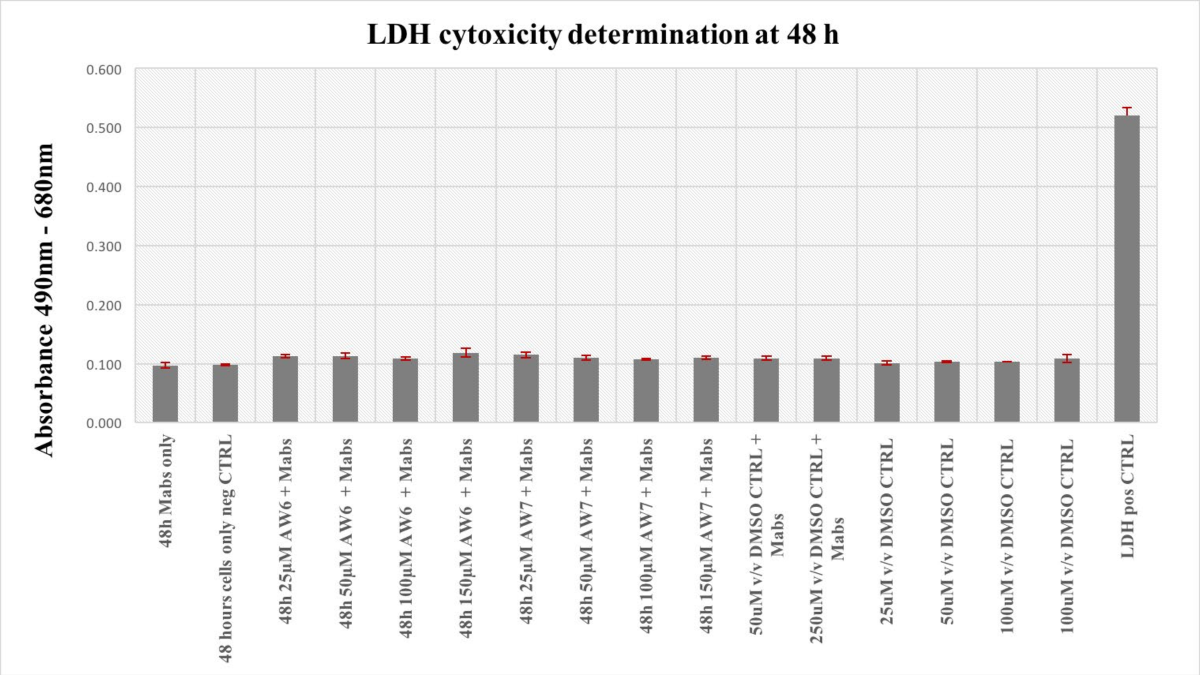

Figure S12: Lactate dehydrogenase (LDH) based cytotoxicity profiles of TrmD lead compounds **AW6** & **AW7** in M. abscessus infected primary human macrophages.

Table S2: X-ray Crystallographic Data Collection & Refinement Statistics for crystal structures described in this study

Ligand	Apo	S-adenosyl methionine (SAM)	S-adenosyl homocysteine (SAH)
PDB Codes	6NVR	6NW6	6NW7
Resolution range (Å)	58.3 - 1.562 (1.618 - 1.562)	56.91 - 1.667 (1.727 - 1.667)	58.48 - 1.481 (1.534 - 1.481)
Space group	P 21 21 21	P 21 21 21	P 21 21 21
Unit cell	73.724 79.128 86.235 90 90 90	75.034 79.24 87.313 90 90 90	75.334 78.254 88.005 90 90 90
Total reflections	555563 (51454)	476241 (48544)	672385 (65190)
Unique reflections	72163 (7142)	61337 (6066)	87015 (8620)
Multiplicity	7.7 (7.2)	7.8 (8.0)	7.7 (7.6)
Completeness (%)	99.98 (99.97)	99.92 (99.95)	99.91 (99.92)
Mean I/sigma(I)	20.51 (2.30)	16.52 (2.23)	28.57 (2.33)
Wilson B-factor	27.07	26.21	22.69
R-merge	0.04883 (0.7835)	0.06756 (0.984)	0.03225 (0.783)
R-meas	0.05237	0.07266	0.03457
CC1/2	0.999 (0.801)	0.998 (0.711)	1 (0.782)
CC*	1 (0.943)	0.999 (0.911)	1 (0.937)
R-work	0.1927 (0.2480)	0.1794 (0.2486)	0.1876 (0.2505)
R-free	0.2056 (0.2892)	0.2033 (0.2799)	0.2088 (0.2662)
Number of non-hydrogen atoms	3692	3677	3675
macromolecules	3337	3371	3321
ligands		5	5
water	355	301	349
Protein residues	432	427	422
RMS(bonds)	0.006	0.012	0.008
RMS(angles)	1.04	1.22	1.11
Ramachandran favored (%)	99	98	99
Ramachandran outliers (%)	0.23	0.23	0
Clashscore	2.86	2.99	1.52
Average B-factor	33.7	34.3	29.7
macromolecules	32.9	33.3	28.8
ligands		57	32.2
solvent	41.3	44.6	38.4

Ligand	Fragment 1	Fragment 2	Fragment 3
**PDB Codes**	**6QO2**	**6QO3**	**6QO4**
**Resolution range (Å)**	56.58 - 1.676 (1.736 - 1.676)	56.27 - 1.645 (1.704 - 1.645)	58.22 - 1.778 (1.841 - 1.778)
**Space group**	P 21 21 21	P 21 21 21	P 21 21 21
**Unit cell**	74.416 77.893 87.113 90 90 90	74.304 79.397 86.148 90 90 90	74.487 78.902 86.272 90 90 90
**Total reflections**	116261 (11471)	124732 (12311)	99055 (9736)
**Unique reflections**	58320 (5759)	62492 (6159)	49601 (4879)
**Multiplicity**	2.0 (2.0)	2.0 (2.0)	2.0 (2.0)
**Completeness (%)**	99.20 (98.51)	99.96 (99.98)	99.99 (99.98)
**Mean I/sigma(I)**	20.53 (2.19)	14.66 (2.23)	18.19 (2.19)
**Wilson B-factor**	27.4	26.22	31.86
**R-merge**	0.01642 (0.3223)	0.02281 (0.2933)	0.01874 (0.353)
**R-meas**	0.02322	0.03226	0.0265
**CC1/2**	1 (0.738)	0.999 (0.795)	0.999 (0.716)
**CC***	1 (0.922)	1 (0.941)	1 (0.913)
**R-work**	0.1711 (0.2335)	0.1745 (0.2533)	0.1800 (0.2729)
**R-free**	0.1981 (0.2688)	0.2072 (0.3030)	0.2134 (0.3163)
**Number of non-hydrogen atoms**	3563	3614	3498
**macromolecules**	3264	3278	3248
**ligands**	24	22	24
**water**	275	314	226
**Protein residues**	427	429	424
**RMS(bonds)**	0.007	0.006	0.007
**RMS(angles)**	1	1.03	1.07
**Ramachandran favored (%)**	99	99	98
**Ramachandran outliers (%)**	0	0.24	0.24
**Clashscore**	1.24	0.77	3.57
**Average B-factor**	34.9	31.6	39.7
**macromolecules**	34.2	30.6	39.4
**ligands**	29.2	23.3	31.7
**solvent**	44.4	43	45.4

Ligand	Fragment 4	Fragment 5	Fragment 6
**PDB Codes**	**6QO6**	**6QOA**	**6QOC**
**Resolution range (Å)**	56.22 - 1.745 (1.807 - 1.745)	58.21 - 1.927 (1.996 - 1.927)	45.82 - 1.838 (1.904 - 1.838)
**Space group**	P 21 21 21	P 21 21 21	P 21 21 21
**Unit cell**	74.077 78.772 86.324 90 90 90	73.763 80.272 84.542 90 90 90	74.205 78.712 86.601 90 90 90
**Total reflections**	103964 (10265)	76955 (7619)	89476 (8818)
**Unique reflections**	52072 (5136)	38594 (3817)	44802 (4414)
**Multiplicity**	2.0 (2.0)	2.0 (2.0)	2.0 (2.0)
**Completeness (%)**	99.97 (99.96)	99.93 (99.95)	99.97 (100.00)
**Mean I/sigma(I)**	19.41 (2.32)	18.01 (2.42)	20.68 (2.41)
**Wilson B-factor**	31.55	36.97	35.4
**R-merge**	0.01638 (0.3367)	0.01996 (0.3006)	0.01579 (0.3125)
**R-meas**	0.02316	0.02822	0.02233
**CC1/2**	0.999 (0.731)	0.999 (0.827)	0.999 (0.772)
**CC***	1 (0.919)	1 (0.951)	1 (0.934)
**R-work**	0.1812 (0.2545)	0.1749 (0.2488)	0.1854 (0.2550)
**R-free**	0.2088 (0.2988)	0.2198 (0.3437)	0.2096 (0.3125)
**Number of non-hydrogen atoms**	3496	3477	3438
**macromolecules**	3223	3276	3229
**ligands**	22	16	24
**water**	251	185	185
**Protein residues**	422	426	420
**RMS(bonds)**	0.007	0.007	0.008
**RMS(angles)**	1.01	1.04	1.08
**Ramachandran favored (%)**	98	99	98
**Ramachandran outliers (%)**	0.72	0.23	1.2
**Clashscore**	2.19	2	2.66
**Average B-factor**	39.1	44.5	43
**macromolecules**	38.5	44.2	42.7
**ligands**	35.4	47.7	39.6
**solvent**	46.6	49	48.3

Ligand	Fragment 7	Fragment 8	Fragment 9
**PDB Codes**	**6QOD**	**6QOE**	**6QOF**
**Resolution range (Å)**	56.3 - 1.852 (1.918 - 1.852)	58.71 - 3.057 (3.166 - 3.057)	45.51 - 1.756 (1.819 - 1.756)
**Space group**	P 21 21 21	P 21 21 21	P 21 21 21
**Unit cell**	74.143 78.379 86.54 90 90 90	74.596 79.739 86.758 90 90 90	73.688 80.749 82.971 90 90 90
**Total reflections**	87002 (8551)	20438 (2020)	99719 (9802)
**Unique reflections**	43567 (4287)	10229 (1011)	50043 (4903)
**Multiplicity**	2.0 (2.0)	2.0 (2.0)	2.0 (2.0)
**Completeness (%)**	99.89 (99.81)	99.71 (99.90)	99.93 (100.00)
**Mean I/sigma(I)**	16.39 (2.24)	13.13 (2.60)	13.29 (2.26)
**Wilson B-factor**	29.65	57.07	28.61
**R-merge**	0.02446 (0.4081)	0.04665 (0.2577)	0.02784 (0.2886)
**R-meas**	0.03459	0.06597	0.03937
**CC1/2**	0.999 (0.642)	0.998 (0.909)	0.999 (0.835)
**CC***	1 (0.884)	0.999 (0.976)	1 (0.954)
**R-work**	0.1843 (0.2990)	0.1891 (0.2725)	0.1802 (0.2419)
**R-free**	0.2116 (0.3414)	0.2808 (0.4267)	0.2135 (0.3003)
**Number of non-hydrogen atoms**	3418	3249	3527
**macromolecules**	3211	3232	3292
**ligands**	22	16	28
**water**	185	1	207
**Protein residues**	422	424	429
**RMS(bonds)**	0.008	0.01	0.007
**RMS(angles)**	1.12	1.28	1.06
**Ramachandran favored (%)**	98	95	99
**Ramachandran outliers (%)**	0.24	0.24	0
**Clashscore**	2.05	5.79	1.52
**Average B-factor**	37.7	51.2	35.1
**macromolecules**	37.4	51.1	34.8
**ligands**	51.9	78.9	26.8
**solvent**	42.4	16.1	42.4

Ligand	Fragment 10	Fragment 11	Fragment 12
PDB Codes	6QOG	6QOH	6QOI
Resolution range (Å)	53.75 - 1.547 (1.603 - 1.547)	58.32 - 1.657 (1.717 - 1.657)	45.78 - 1.857 (1.923 - 1.857)
Space group	P 21 21 21	P 21 21 21	P 21 21 21
Unit cell	73.75 78.513 86.707 90 90 90	73.652 79.108 86.318 90 90 90	74.107 79.509 85.468 90 90 90
Total reflections	147636 (14608)	120782 (11932)	86344 (8474)
Unique reflections	73971 (7327)	60509 (5971)	43267 (4241)
Multiplicity	2.0 (2.0)	2.0 (2.0)	2.0 (2.0)
Completeness (%)	99.93 (99.92)	99.96 (99.97)	99.96 (100.00)
Mean I/sigma(I)	21.56 (2.35)	15.01 (2.24)	17.45 (2.31)
Wilson B-factor	24.52	26.86	36.31
R-merge	0.01437 (0.316)	0.02302 (0.333)	0.01907 (0.3199)
R-meas	0.02032	0.03255	0.02696
CC1/2	1 (0.783)	0.999 (0.775)	0.999 (0.777)
CC*	1 (0.937)	1 (0.934)	1 (0.935)
R-work	0.1786 (0.2394)	0.1889 (0.2515)	0.1947 (0.2644)
R-free	0.2010 (0.2474)	0.2246 (0.2902)	0.2251 (0.3098)
Number of non-hydrogen atoms	3608	3528	3317
macromolecules	3279	3224	3152
ligands	11	27	20
water	318	277	145
Protein residues	428	419	417
RMS(bonds)	0.006	0.006	0.007
RMS(angles)	1.04	1.04	1.07
Ramachandran favored (%)	100	98	98
Ramachandran outliers (%)	0	0.72	0.73
Clashscore	0.92	3.44	2.09
Average B-factor	32.4	34.6	44.5
macromolecules	31.3	33.9	44.4
ligands	36.4	38.1	50.5
solvent	43	41.6	47.7

Ligand	Fragment 13	Fragment 14	Fragment 15
PDB Codes	6QOJ	6QOK	6QOL
Resolution range (Å)	45.76 - 2.023 (2.095 - 2.023)	58.18 - 1.476 (1.528 - 1.476)	56.3 - 1.903 (1.97 - 1.903)
Space group	P 21 21 21	P 21 21 21	P 21 21 21
Unit cell	74.01 80.498 84.284 90 90 90	73.702 78.625 86.484 90 90 90	74.008 78.608 86.729 90 90 90
Total reflections	66991 (6581)	169607 (16630)	80642 (7953)
Unique reflections	33536 (3293)	85013 (8354)	40390 (3982)
Multiplicity	2.0 (2.0)	2.0 (2.0)	2.0 (2.0)
Completeness (%)	99.90 (99.88)	99.96 (99.89)	99.98 (100.00)
Mean I/sigma(I)	17.29 (2.17)	21.66 (2.27)	18.24 (2.38)
Wilson B-factor	46.57	21.06	36.27
R-merge	0.01689 (0.368)	0.01465 (0.3515)	0.01889 (0.3324)
R-meas	0.02389	0.02072	0.02671
CC1/2	1 (0.812)	1 (0.72)	1 (0.745)
CC*	1 (0.947)	1 (0.915)	1 (0.924)
R-work	0.1959 (0.3020)	0.1700 (0.2838)	0.1855 (0.2632)
R-free	0.2366 (0.3695)	0.1987 (0.3133)	0.2151 (0.3458)
Number of non-hydrogen atoms	3359	3689	3395
macromolecules	3270	3323	3236
ligands	22	32	22
water	67	334	137
Protein residues	429	431	422
RMS(bonds)	0.007	0.006	0.007
RMS(angles)	1.05	1.05	1.02
Ramachandran favored (%)	100	99	99
Ramachandran outliers (%)	0	0	0.24
Clashscore	2.32	1.21	2.66
Average B-factor	60.1	30.4	45
macromolecules	60.2	29.1	45
ligands	47.8	45.6	37.5
solvent	55.9	41.5	45.6

Ligand	Fragment 16	Fragment 17	Fragment 18
**PDB Codes**	**6QOM**	**6QON**	**6QOO**
**Resolution range (Å)**	54.18 - 1.897 (1.965 - 1.897)	56.33 - 1.81 (1.875 - 1.81)	58.19 - 1.939 (2.008 - 1.939)
**Space group**	P 21 21 21	P 21 21 21	P 21 21 21
**Unit cell**	73.955 79.592 85.853 90 90 90	74.02 78.919 86.836 90 90 90	73.574 80.037 84.737 90 90 90
**Total reflections**	81353 (7977)	93844 (9248)	75366 (7415)
**Unique reflections**	40737 (3993)	47009 (4630)	37761 (3709)
**Multiplicity**	2.0 (2.0)	2.0 (2.0)	2.0 (2.0)
**Completeness (%)**	99.90 (99.90)	99.93 (99.96)	99.95 (100.00)
**Mean I/sigma(I)**	17.55 (2.39)	17.12 (2.33)	15.39 (2.19)
**Wilson B-factor**	37.82	28.85	37.61
**R-merge**	0.01689 (0.34)	0.02202 (0.3552)	0.02256 (0.3031)
**R-meas**	0.02389	0.03114	0.03191
**CC1/2**	1 (0.748)	0.999 (0.708)	0.999 (0.834)
**CC***	1 (0.925)	1 (0.91)	1 (0.954)
**R-work**	0.1924 (0.2564)	0.1745 (0.2703)	0.1815 (0.2483)
**R-free**	0.2296 (0.2935)	0.2061 (0.3146)	0.2263 (0.3209)
**Number of non-hydrogen atoms**	3354	3587	3399
**macromolecules**	3212	3326	3245
**ligands**	10	29	21
**water**	132	232	133
**Protein residues**	419	431	424
**RMS(bonds)**	0.009	0.008	0.007
**RMS(angles)**	1.21	1.13	1.03
**Ramachandran favored (%)**	97	99	98
**Ramachandran outliers (%)**	1.2	0	0.24
**Clashscore**	2.37	2.41	1.86
**Average B-factor**	47	36	46.5
**macromolecules**	46.9	35.3	46.2
**ligands**	66.6	62.9	65.7
**solvent**	47.7	43.4	49.4

Ligand	Fragment 19	Fragment 20	Fragment 21
**PDB Codes**	**6QOP**	**6QOQ**	**6QOR**
**Resolution range (Å)**	58.21 - 1.912 (1.98 - 1.912)	56.24 - 1.666 (1.726 - 1.666)	45.75 - 1.671 (1.73 - 1.671)
**Space group**	P 21 21 21	P 21 21 21	P 21 21 21
**Unit cell**	74.133 78.896 86.245 90 90 90	74.048 78.489 86.472 90 90 90	74.915 77.088 87.278 90 90 90
**Total reflections**	79389 (7856)	118831 (11748)	118266 (11615)
**Unique reflections**	39796 (3935)	59496 (5884)	59237 (5823)
**Multiplicity**	2.0 (2.0)	2.0 (2.0)	2.0 (2.0)
**Completeness (%)**	99.94 (99.97)	99.97 (99.98)	99.97 (99.98)
**Mean I/sigma(I)**	16.88 (2.40)	17.71 (2.30)	16.47 (2.35)
**Wilson B-factor**	38.79	27.16	25.31
**R-merge**	0.02026 (0.3011)	0.01919 (0.3273)	0.01969 (0.293)
**R-meas**	0.02865	0.02714	0.02784
**CC1/2**	0.999 (0.759)	0.999 (0.722)	0.999 (0.769)
**CC***	1 (0.929)	1 (0.916)	1 (0.932)
**R-work**	0.1899 (0.2702)	0.1723 (0.2409)	0.1727 (0.2384)
**R-free**	0.2227 (0.3457)	0.1934 (0.2747)	0.2064 (0.2593)
**Number of non-hydrogen atoms**	3368	3573	3619
**macromolecules**	3217	3290	3263
**ligands**	29	20	27
**water**	122	263	329
**Protein residues**	419	426	421
**RMS(bonds)**	0.007	0.006	0.009
**RMS(angles)**	1.02	1.01	1.2
**Ramachandran favored (%)**	98	99	97
**Ramachandran outliers (%)**	0.72	0	0
**Clashscore**	1.72	1.99	2
**Average B-factor**	48.7	34.6	33.5
**macromolecules**	48.5	33.9	32.6
**ligands**	62.9	39.3	31.5
**solvent**	50.8	43.3	43

Ligand	Fragment 22	Fragment 23	Fragment 24
**PDB Codes**	**6QOS**	**6QOT**	**6QOU**
**Resolution range (Å)**	58.69 - 2.052 (2.126 - 2.052)	57.06 - 1.618 (1.676 - 1.618)	58.63 - 1.556 (1.611 - 1.556)
**Space group**	P 21 21 21	P 21 21 21	P 21 21 21
**Unit cell**	74.913 81.357 84.754 90 90 90	75.291 78.925 87.458 90 90 90	75.188 79.07 87.396 90 90 90
**Total reflections**	253108 (24437)	133947 (13225)	150513 (14876)
**Unique reflections**	33037 (3253)	67062 (6632)	75337 (7445)
**Multiplicity**	7.7 (7.5)	2.0 (2.0)	2.0 (2.0)
**Completeness (%)**	99.89 (99.94)	99.91 (99.83)	99.95 (99.95)
**Mean I/sigma(I)**	15.59 (2.41)	26.32 (2.33)	31.53 (2.13)
**Wilson B-factor**	39.66	26.25	25.28
**R-merge**	0.06667 (0.7334)	0.01205 (0.3141)	0.009729 (0.3395)
**R-meas**	0.07153	0.01704	0.01376
**CC1/2**	0.999 (0.857)	1 (0.784)	1 (0.753)
**CC***	1 (0.961)	1 (0.937)	1 (0.927)
**R-work**	0.1911 (0.2634)	0.1831 (0.2301)	0.1835 (0.2416)
**R-free**	0.2289 (0.2944)	0.2143 (0.2640)	0.2060 (0.2643)
**Number of non-hydrogen atoms**	3432	3538	3574
**macromolecules**	3267	3245	3240
**ligands**	25	32	24
**water**	140	261	310
**Protein residues**	427	424	422
**RMS(bonds)**	0.008	0.009	0.009
**RMS(angles)**	1.03	1.03	1.04
**Ramachandran favored (%)**	99	99	98
**Ramachandran outliers (%)**	0	0	0
**Clashscore**	1.85	0.62	1.4
**Average B-factor**	50	33.8	33.8
**macromolecules**	49.9	33.1	32.9
**ligands**	60.2	25.5	27.1
**solvent**	50.2	43.4	44.3

Ligand	Fragment 25	Fragment 26	Fragment 27
**PDB Codes**	**6QOV**	**6QOW**	**6QOX**
**Resolution range (Å)**	58.68 - 1.548 (1.603 - 1.548)	58.48 - 1.525 (1.58 - 1.525)	58.7 - 1.739 (1.801 - 1.739)
**Space group**	P 21 21 21	P 21 21 21	P 21 21 21
**Unit cell**	75.033 79.041 87.583 90 90 90	75.693 78.16 88.152 90 90 90	74.963 78.46 88.483 90 90 90
**Total reflections**	152376 (15078)	159811 (15805)	108501 (10712)
**Unique reflections**	76391 (7557)	80070 (7918)	54302 (5361)
**Multiplicity**	2.0 (2.0)	2.0 (2.0)	2.0 (2.0)
**Completeness (%)**	99.86 (99.88)	99.81 (99.60)	99.96 (100.00)
**Mean I/sigma(I)**	25.49 (2.23)	25.95 (2.36)	24.28 (2.26)
**Wilson B-factor**	22.28	22.17	29.04
**R-merge**	0.01377 (0.3227)	0.01348 (0.3093)	0.01492 (0.3145)
**R-meas**	0.01947	0.01906	0.02109
**CC1/2**	1 (0.778)	1 (0.782)	1 (0.778)
**CC***	1 (0.935)	1 (0.937)	1 (0.935)
**R-work**	0.1822 (0.2402)	0.1757 (0.2441)	0.1841 (0.2644)
**R-free**	0.2066 (0.2733)	0.1968 (0.2812)	0.2135 (0.3040)
**Number of non-hydrogen atoms**	3544	3685	3563
**macromolecules**	3230	3269	3262
**ligands**	28	33	14
**water**	286	383	287
**Protein residues**	419	422	425
**RMS(bonds)**	0.009	0.011	0.009
**RMS(angles)**	1.02	1.19	1.02
**Ramachandran favored (%)**	98	98	98
**Ramachandran outliers (%)**	0	0	0
**Clashscore**	1.25	2.61	0.93
**Average B-factor**	30.5	30.3	36.9
**macromolecules**	29.7	29.1	36.2
**ligands**	21	30.1	44
**solvent**	40.3	40.4	45.2

Ligand	AW1	AW2	AW3
**PDB Codes**	**6QQS**	**6QQX**	**6QQY**
**Resolution range (Å)**	54.15 - 1.758 (1.821 - 1.758)	56.49 - 2.693 (2.789 - 2.693)	54.03 - 1.49 (1.543 - 1.49)
**Space group**	P 21 21 21	P 21 21 21	P 21 21 21
**Unit cell**	74.357 79.028 85.537 90 90 90	74.614 78.22 86.471 90 90 90	74.51 78.45 86.04 90 90 90
**Total reflections**	386872 (38846)	125554 (13048)	165480 (16281)
**Unique reflections**	50846 (5047)	14535 (1440)	82869 (8156)
**Multiplicity**	7.6 (7.7)	8.6 (9.1)	2.0 (2.0)
**Completeness (%)**	99.98 (100.00)	99.92 (99.93)	99.95 (99.96)
**Mean I/sigma(I)**	18.44 (2.21)	8.33 (2.19)	22.56 (1.42)
**Wilson B-factor**	30.86	36.88	24.83
**R-merge**	0.05835 (1.035)	0.2334 (1.087)	0.01375 (0.5068)
**R-meas**	0.06277	0.2489	0.01945
**CC1/2**	0.999 (0.716)	0.984 (0.77)	1 (0.59)
**CC***	1 (0.914)	0.996 (0.933)	1 (0.861)
**R-work**	0.1829 (0.2675)	0.1778 (0.2386)	0.1853 (0.2846)
**R-free**	0.2081 (0.2996)	0.2698 (0.3798)	0.1997 (0.3104)
**Number of non-hydrogen atoms**	3534	3372	3701
**macromolecules**	3282	3263	3275
**ligands**	30	49	48
**water**	222	60	378
**Protein residues**	423	422	422
**RMS(bonds)**	0.007	0.01	0.008
**RMS(angles)**	1.13	1.16	1.11
**Ramachandran favored (%)**	99	97	99
**Ramachandran outliers (%)**	0.24	0.24	0
**Clashscore**	3.21	3.69	4.13
**Average B-factor**	39.9	34	31.4
**macromolecules**	39.5	34.1	30.4
**ligands**	27.2	25.7	24
**solvent**	47.1	32.8	41

**Ligand**	AW5	AW6	AW7
**PDB Codes**	**6QR6**	**6QR5**	**6QR8**
**Resolution range (Å)**	45.85 - 1.707 (1.768 - 1.707)	56.27 - 1.81 (1.875 - 1.81)	57.2 - 2.149 (2.225 - 2.149)
**Space group**	P 21 21 21	P 21 21 21	P 21 21 21
**Unit cell**	74.95 77.483 87.347 90 90 90	74.183 77.985 86.363 90 90 90	74.85 76.329 86.381 90 90 90
**Total reflections**	484104 (49526)	92506 (9074)	260137 (26060)
**Unique reflections**	55948 (5512)	46297 (4542)	27601 (2722)
**Multiplicity**	8.7 (9.0)	2.0 (2.0)	9.4 (9.6)
**Completeness (%)**	99.97 (100.00)	99.96 (100.00)	99.94 (100.00)
**Mean I/sigma(I)**	20.79 (2.35)	26.01 (2.49)	10.58 (2.37)
**Wilson B-factor**	31.78	35.93	40.03
**R-merge**	0.0484 (0.8462)	0.01154 (0.2436)	0.1318 (1.18)
**R-meas**	0.05156	0.01632	0.1395
**CC1/2**	0.999 (0.781)	1 (0.873)	0.998 (0.727)
**CC***	1 (0.936)	1 (0.966)	0.999 (0.918)
**R-work**	0.1807 (0.2398)	0.1922 (0.2614)	0.1848 (0.2478)
**R-free**	0.2014 (0.2654)	0.2123 (0.3004)	0.2262 (0.2750)
**Number of non-hydrogen atoms**	3559	3504	3408
**macromolecules**	3277	3248	3245
**ligands**	65	56	64
**water**	217	200	99
**Protein residues**	422	424	423
**RMS(bonds)**	0.008	0.007	0.009
**RMS(angles)**	1.08	1.23	1.13
**Ramachandran favored (%)**	99	99	99
**Ramachandran outliers (%)**	0	0	0
**Clashscore**	2.44	2.78	2
**Average B-factor**	37.6	41	46.7
**macromolecules**	37.2	40.7	46.6
**ligands**	35.2	39.1	45.7
**solvent**	44.2	45.4	48.4

Synthetic Chemistry

Table S3: Correlation table for compound identification numbers in this paper and in the corresponding publication (7), where the in-depth medicinal chemistry and synthesis strategy is reported.

**Compound ID**	**Whitehouse, A.J., *et al* (7) compound ID**	**PDB codes**
AW1	23	6QQS
AW2	24f	6QQX
AW3	26f	6QQY
AW4	28	Structure not determined
AW5	29a	6QR6
AW6	31a	6QR5
AW7	29d	6QR8

General Chemistry

All reactions were carried out in oven-dried glassware under a positive pressure of dry nitrogen atmosphere. Temperatures of 0 and -78 °C were obtained by submerging the reaction vessel in a bath containing either ice or a mixture of solid CO_2 pellets and acetone respectively. The solvents DCM, ethyl acetate, acetonitrile, methanol, petroleum ether and toluene were distilled over calcium hydride under a dry nitrogen atmosphere prior to use, with THF distilled over a mixture of lithium aluminium hydride, calcium hydride and triphenylphosphine. DMF was purchased as anhydrous from commercial suppliers, with ethanol and acetic acid obtained in the absolute and glacial forms respectively. All purchased chemicals were used as received. Solutions of Na_2CO_3, $NaHCO_3$, NaCl (brine) and NH_4Cl were aqueous and saturated.

Flash column chromatography was performed using automated Biotage® Isolera™ Spektra purification systems with appropriately sized Biotage® SNAP cartridges, containing either KP 50 μm silica in 'normal phase' purification or HP-sphere 25 μm C18 silica in 'reverse phase' purification. Microwave-heating was performed using a Biotage® Initiator+ system with sealed Biotage® microwave reaction vials. Analytical thin layer chromatography (TLC) was performed using Merck glass-backed silica plates, with visualization by 254 or 365 nm ultraviolet light.

Liquid chromatography mass spectrometry (LCMS) was carried out using a Waters® Acquity UPLC® H-Class system, with samples run on a solvent gradient from 0 to 95% acetonitrile in water (+ 0.1% formic acid) over 4 minutes. Peaks corresponding to desired product are described, including the retention time (rt) and % purity by integration. High resolution mass spectrometry (HRMS) was mainly performed using ThermoFinnigan Orbitrap Classic, Waters® LCT Premier™ or Waters® Vion™ IMS QTof systems. A Perkin-Elmer® Spectrum One FT-IR spectrometer fitted with a universal attenuated total reflectance accessory was used to record infrared spectra, with wavelengths of maximum absorbance (ν_{max}) quoted in wavenumbers

(cm^{-1}) for signals outside of the fingerprint region (br = broad). Only peaks corresponding to key functional groups were characterized. Nuclear magnetic resonance (NMR) spectra were recorded in the indicated deuterated solvents with Avance™ III HD (400 MHz), QNP Cryoprobe (400 MHz) or DCH Cryoprobe (500 MHz) Bruker spectrometers. ^{1}H NMR data are presented in the following order: chemical shift (in ppm on a δ scale relative to the residual solvent resonance peak), integration, multiplicity (s = singlet, d = doublet, t = triplet, q = quartet, m = multiplet) and coupling constant (J, in Hz). ^{13}C NMR spectra were proton-decoupled, with chemical shifts recorded and further description provided for certain peaks (br = broad). A combination of TLC and LCMS analysis was used to monitor reactions. All tested compounds possessed a purity of at least 95% as determined by LCMS analysis.

Synthesis of AW1

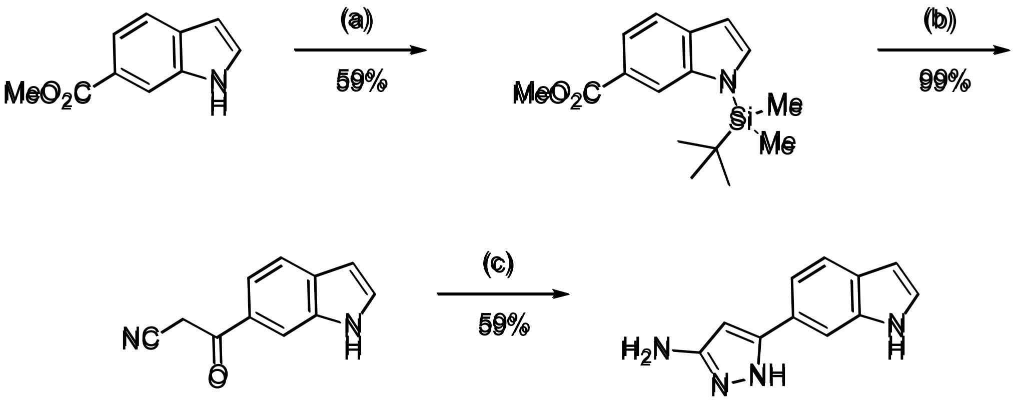

Scheme S1: Reagents and conditions: (a) tert-butyldimethylsilyl chloride (1 M in DCM), sodium hydride, THF, 0 °C to room temperature; (b) (i) acetonitrile, n-butyllithium (1.6 M in hexanes), toluene, -78 °C to room temperature (ii) tetra-n-butylammonium fluoride (1 M in THF), THF; (c) hydrazine monohydrate, ethanol, reflux.

methyl 1-(*tert*-butyldimethylsilyl)-1H-indole-6-carboxylate

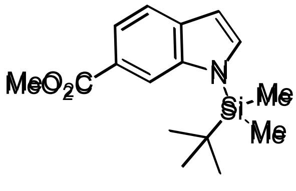

A solution of methyl 1H-indole-6-carboxylate (0.500 g, 2.71 mmol) in THF (10 mL) was added dropwise at 0 °C to a stirred suspension of sodium hydride (60% in mineral oil, 0.141 g, 3.52 mmol) in THF (10 mL). The reaction mixture was warmed to room temperature and stirred over 30 minutes. *tert*-Butyldimethylsilyl chloride (1 M in DCM, 4.1 mL, 4.1 mmol) was added dropwise at 0 °C to the reaction mixture. The reaction mixture was warmed to room temperature and stirred over 10 hours. NH_4Cl solution (20 mL) was added dropwise at 0 °C. The product was extracted into diethyl ether (2 x 50 mL). The combined organic extracts were

washed (brine), dried ($MgSO_4$) and concentrated *in vacuo*. Purification by flash column chromatography (0 – 20% ethyl acetate in petroleum ether) gave methyl 1-(*tert*-butyldimethylsilyl)-1H-indole-6-carboxylate (0.462 g, 59% yield).

LCMS (ESI+): m/z 290.2 $[M + H]^+$, rt 3.05 minutes, 100%; ^1H NMR (500 MHz, $CDCl_3$) 8.29-8.27 (1H, m), 7.80 (1H, dd, J = 8.3, 1.4 Hz), 7.63 (1H, d, J = 8.4 Hz), 7.34 (1H, d, J = 3.2 Hz), 6.65 (1H, dd, J = 3.1, 0.9 Hz), 3.93 (3H, s), 0.93 (9H, s), 0.65 (6H, s); ^{13}C NMR (125 MHz, $CDCl_3$) 168.5, 140.5, 135.3, 134.6, 123.2, 121.0, 120.2, 116.2, 105.2, 52.1, 26.4, 19.5, -3.8; spectroscopic data consistent with literature (8).

3-(1H-indol-6-yl)-3-oxopropanenitrile

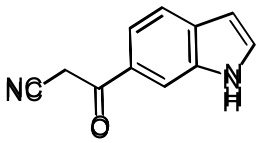

n-Butyllithium (1.6 M in hexanes, 3.0 mL, 4.8 mmol) was added dropwise at -78 °C to a mixture of acetonitrile (0.180 mL, 3.45 mmol) and toluene (3 mL). The reaction mixture was stirred at -78 °C over 30 minutes. A solution of methyl 1-(*tert*-butyldimethylsilyl)-1H-indole-6-carboxylate (0.200 g, 0.691 mmol) in toluene (2 mL) was added dropwise at -78 °C over 30 minutes. The reaction mixture was stirred at -78 °C over 1 hour, then warmed to room temperature over 1 hour. Aqueous HCl (3 M, 20 mL) was added dropwise at 0 °C. The intermediate was extracted into ethyl acetate (2 x 25 mL). The combined organic extracts were washed (brine), dried ($MgSO_4$) and concentrated *in vacuo*. The crude residue was dissolved in THF (2 mL) and tetra-*n*-butylammonium fluoride (1 M in THF, 0.760 mL, 0.760 mmol) was added dropwise. The reaction mixture was stirred over 20 minutes, then $NaHCO_3$ solution (30 mL) was added dropwise. The product was extracted into DCM (3 x 25 mL). The combined organic extracts were washed (brine), dried ($MgSO_4$) and concentrated *in vacuo*. Purification by flash column chromatography (0 – 20% methanol in DCM) gave 3-(1H-indol-6-yl)-3-oxopropanenitrile (0.131 g, 99% yield).

LCMS (ESI-): m/z 183.1 $[M - H]^-$, rt 1.91 minutes, 100%; ^1H NMR (500 MHz, CD_3CN) 9.74 (1H, br s), 8.07 (1H, s), 7.69 (1H, d, J = 8.4 Hz), 7.62 (1H, dd, J = 8.4, 1.5 Hz), 7.53 (1H, t, J = 2.8 Hz), 6.61-6.57 (1H, m), 4.36 (2H, s); ^{13}C NMR (125 MHz, CD_3CN) 189.5, 136.2, 133.6, 131.0, 129.3, 121.3, 120.1, 116.4, 113.9, 103.2, 30.6; v_{max}/cm^{-1} 3326 (N-H), 1675 (C=O), 1611, 1501; HRMS (ESI)+: m/z calculated for $[C_{11}H_8N_2O + H]^+$ = 185.0709, observed 185.0708.

5-(1H-indol-6-yl)-1H-pyrazol-3-amine (AW1)

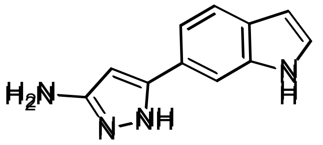

Hydrazine monohydrate (0.101 mL, 0.814 mmol) was added to a solution of 3-(1H-indol-6-yl)-3-oxopropanenitrile (50 mg, 0.27 mmol) in ethanol (5 mL). The reaction mixture was heated under reflux for 11 hours. Further hydrazine monohydrate (0.101 mL, 0.814 mmol) was added to the reaction mixture. The reaction mixture was heated under reflux for 1 hour, then concentrated *in vacuo*. Purification by flash column chromatography (0 – 10% methanol in DCM) gave 5-(1H-indol-6-yl)-1H-pyrazol-3-amine (32 mg, 59% yield).

LCMS (ESI+): m/z 199.1 $[M + H]^+$, (ESI-): m/z 197.0 $[M - H]^-$, rt 1.98 minutes, 100%; ^{1}H NMR (400 MHz, CD_3OD) 7.65 (1H, s), 7.56 (1H, d, J = 8.3 Hz), 7.30 (1H, dd, J = 8.3, 1.5 Hz), 7.26 (1H, d, J = 3.1 Hz), 6.44 (1H, dd, J = 3.1, 0.7 Hz), 5.93 (1H, s); ^{13}C NMR (125 MHz, CD_3CN) 155.2, 147.7, 137.1, 128.8, 126.9, 125.5, 121.4, 108.8, 102.5, 89.3 (1 peak missing); ν_{max}/cm^{-1} 3389 (N-H), 3250 (br, N-H), 1616, 1584, 1561, 1516; HRMS (ESI+): m/z calculated for $[C_{11}H_{10}N_4 + H]^+$ = 199.0978, observed 199.0973.

Synthesis of AW2/ AW3

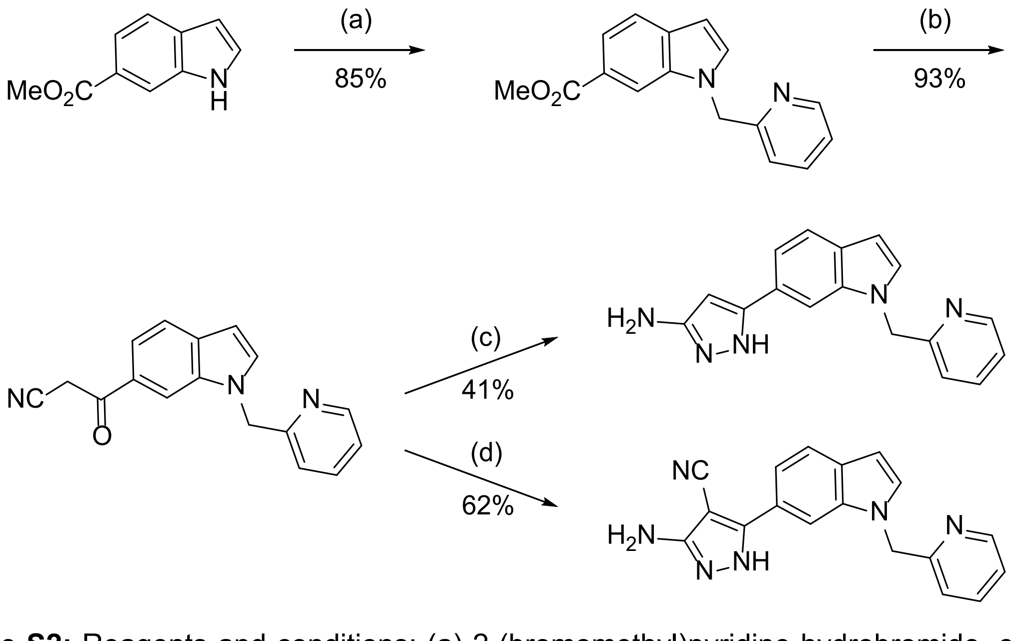

Scheme S2: Reagents and conditions: (a) 2-(bromomethyl)pyridine hydrobromide, caesium carbonate, acetonitrile, reflux; (b) acetonitrile, n-butyllithium (1.6 M in hexanes), THF, -78 °C; (c) hydrazine monohydrate, ethanol, reflux; (d) (i) trichloroacetonitrile, sodium acetate, ethanol (ii) hydrazine monohydrate, ethanol, reflux.

methyl 1-(pyridin-2-ylmethyl)-1H-indole-6-carboxylate

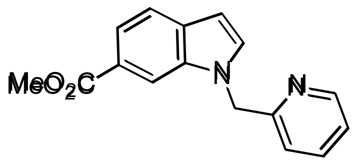

2-(Bromomethyl)pyridine hydrobromide (0.476 mg, 1.88 mmol) was added to a suspension of methyl 1H-indole-6-carboxylate (0.300 g, 1.71 mmol) and caesium carbonate (1.39 g, 4.28 mmol) in acetonitrile (15 mL). The reaction mixture was heated under reflux for 15 hours, then concentrated *in vacuo*. Water (20 mL) was added and the product extracted into DCM (3 x 25 mL). The combined organic extracts were washed (brine), dried ($MgSO_4$) and concentrated *in vacuo*. Purification by flash column chromatography (0 – 50% ethyl acetate in petroleum ether) gave methyl 1-(pyridin-2-ylmethyl)-1H-indole-6-carboxylate (0.386 g, 85% yield).

LCMS (ESI+): m/z 267.3 $[M + H]^+$, rt 1.82 minutes, 100%; ^1H NMR (400 MHz, $CDCl_3$) 8.60 (1H, dq, J = 4.9, 0.8 Hz), 8.09-8.05 (1H, m), 7.82 (1H, dd, J = 8.3, 1.4 Hz), 7.67 (1H, dd, J = 8.3, 0.5 Hz), 7.54 (1H, td, J = 7.7, 1.8 Hz), 7.37 (1H, d, J = 3.2 Hz), 7.21-7.14 (1H, m), 6.70 (1H, d, J = 7.8 Hz), 6.63 (1H, dd, J = 3.1, 0.8 Hz), 5.52 (2H, s), 3.90 (3H, s); ^{13}C NMR (100 MHz, $CDCl_3$) 168.2, 157.1, 149.7, 137.3, 135.8, 132.5, 131.8, 123.8, 122.8, 120.9, 120.8, 112.1, 102.7, 52.10, 52.08 (1 peak missing); v_{max}/cm^{-1} 1717 (C=O), 1593, 1505; HRMS (ESI)+: m/z calculated for $[C_{16}H_{14}N_2O_2 + H]^+$ = 267.1128, observed 267.1136.

3-oxo-3-(1-(pyridin-2-ylmethyl)-1H-indol-6-yl)propanenitrile

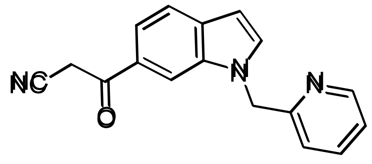

n-Butyllithium (1.6 M in hexanes, 2.6 mL, 4.2 mmol) was added dropwise at -78 °C to a mixture of acetonitrile (0.369 mL, 7.06 mmol) and THF (5 mL). The reaction mixture was stirred at -78 °C over 30 minutes. A solution of methyl 1-(pyridin-2-ylmethyl)-1H-indole-6-carboxylate (0.376 g, 1.41 mmol) in THF (5 mL) was added dropwise at -78 °C over 30 minutes. The reaction mixture was stirred at -78 °C over 30 minutes. Aqueous HCl (1 M, 15 mL) was added dropwise at 0 °C. The product was extracted into DCM (3 x 50 mL). The combined organic extracts were washed (brine), dried ($MgSO_4$) and concentrated *in vacuo* to afford 3-oxo-3-(1-(pyridin-2-ylmethyl)-1H-indol-6-yl)propanenitrile (0.363 g, 93% yield).

LCMS (ESI+): m/z 276.2 $[M + H]^+$, (ESI-): m/z 274.2 $[M - H]^-$, rt 1.65 minutes, 100%; ^1H NMR (400 MHz, $(CD_3)_2SO$) 8.55 (1H, d, J = 4.8 Hz), 8.19 (1H, s), 7.81 (1H, d, J = 3.1 Hz), 7.76 (1H, td, J = 7.6, 1.7 Hz), 7.70 (1H, d, J = 8.4 Hz), 7.61 (1H, dd, J = 8.4, 1.4 Hz), 7.31 (1H, dd, J = 7.5, 4.9 Hz), 7.06 (1H, d, J = 7.8 Hz), 6.64 (1H, d, J = 3.1 Hz), 5.66 (2H, s), 4.74 (2H, s); ^{13}C

NMR (100 MHz, $(CD_3)_2SO$) 189.2, 156.8, 149.1, 137.6, 135.3, 134.3, 132.7, 128.1, 122.9, 121.3, 120.5, 119.3, 116.3, 111.8, 102.0, 50.8, 29.9; v_{max}/cm^{-1} 1673 (C=O), 1606, 1590, 1502; HRMS (ESI)+: m/z calculated for $[C_{17}H_{13}N_3O + H]^+$ = 276.1131, observed 276.1126.

5-(1-(pyridin-2-ylmethyl)-1H-indol-6-yl)-1H-pyrazol-3-amine (AW2)

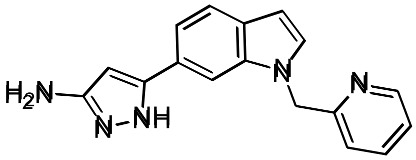

Hydrazine monohydrate (0.300 mL, 6.17 mmol) was added to a solution of 3-oxo-3-(1-(pyridin-2-ylmethyl)-1H-indol-6-yl)propanenitrile (0.170 g, 0.617 mmol) in ethanol (15 mL). The reaction mixture was heated under reflux for 13 hours, then concentrated *in vacuo*. Purification by flash column chromatography (0 – 20% methanol in DCM) gave 5-(1-(pyridin-2-ylmethyl)-1H-indol-6-yl)-1H-pyrazol-3-amine (73 mg, 41% yield).

LCMS (ESI+): m/z 290.3 $[M + H]^+$, rt 1.43 minutes, 100%; ^1H NMR (400 MHz, CD_3OD) 8.56-8.50 (1H, m), 7.68 (1H, td, J = 7.7, 1.8 Hz), 7.63-7.58 (2H, m), 7.38-7.33 (2H, m), 7.28 (1H, dd, J = 7.6, 5.0 Hz), 6.89 (1H, d, J = 7.9 Hz), 6.55 (1H, d, J = 3.1 Hz), 5.90 (1H, s), 5.52 (2H, s); ^{13}C NMR (125 MHz, CD_3OD) 158.8, 155.7 (br), 150.0, 148.4 (br), 139.2, 137.9, 130.9, 130.4, 125.8, 124.1, 122.7, 122.2, 118.7, 107.5, 103.1, 90.2, 52.2; v_{max}/cm^{-1} 3150 (br, N-H), 2916, 1597, 1585, 1504; HRMS (ESI+): m/z calculated for $[C_{17}H_{15}N_5 + H]^+$ = 290.1400, observed 290.1405.

3-amino-5-(1-(pyridin-2-ylmethyl)-1H-indol-6-yl)-1H-pyrazole-4-carbonitrile (AW3)

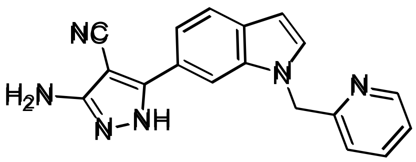

Trichloroacetonitrile (0.186 mL, 1.85 mmol) was added to a suspension of 3-oxo-3-(1-(pyridin-2-ylmethyl)-1H-indol-6-yl) propanenitrile (0.170 g, 0.617 mmol) and sodium acetate (0.253 g, 3.09 mmol) in ethanol (5 mL). The reaction mixture was stirred over 90 minutes. The reaction mixture was concentrated *in vacuo*, then water (20 mL) was added. The intermediate was extracted into DCM (3 x 25 mL). The combined organic extracts were washed (brine), dried ($MgSO_4$) and concentrated *in vacuo*. The crude residue was dissolved in ethanol (10 mL) and hydrazine monohydrate (0.300 mL, 6.17 mmol) was added. The reaction mixture was heated under reflux for 15 hours, then concentrated *in vacuo*. Purification by flash column chromatography (0 - 100% ethyl acetate in petroleum ether, 0 – 10% methanol in DCM) gave 3-amino-5-(1-(pyridin-2-ylmethyl)-1H-indol-6-yl)-1H-pyrazole-4-carbonitrile (0.121 g, 62% yield).

LCMS (ESI+): m/z 315.2 $[M + H]^+$, (ESI-): m/z 313.1 $[M - H]^-$, rt 1.53 minutes, 100%; ^{1}H NMR (400 MHz, $CDCl_3$) 8.46 (1H, d, J = 5.0 Hz), 7.70 (1H, s), 7.67 (1H, d, J = 8.3 Hz), 7.62-7.53 (2H, m), 7.31 (1H, d, J = 3.1 Hz), 7.14 (1H, dd, J = 7.6, 4.9 Hz), 6.91 (1H, d, J = 7.8 Hz), 6.58 (1H, d, J = 3.2 Hz), 5.38 (2H, s); ^{13}C NMR (125 MHz, $CDCl_3$) 157.8, 156.7, 149.2, 149.1, 137.8, 136.1, 130.9, 130.4, 123.3, 122.1, 121.8, 121.0, 118.1, 115.6, 107.6, 102.7, 76.2, 52.3; v_{max}/cm^{-1} 3200 (br, N-H), 2211 (C≡N), 1621, 1592, 1503; HRMS (ESI+): m/z calculated for $[C_{18}H_{14}N_6 + H]^+$ = 315.1353, observed 315.1338.

Synthesis of AW4

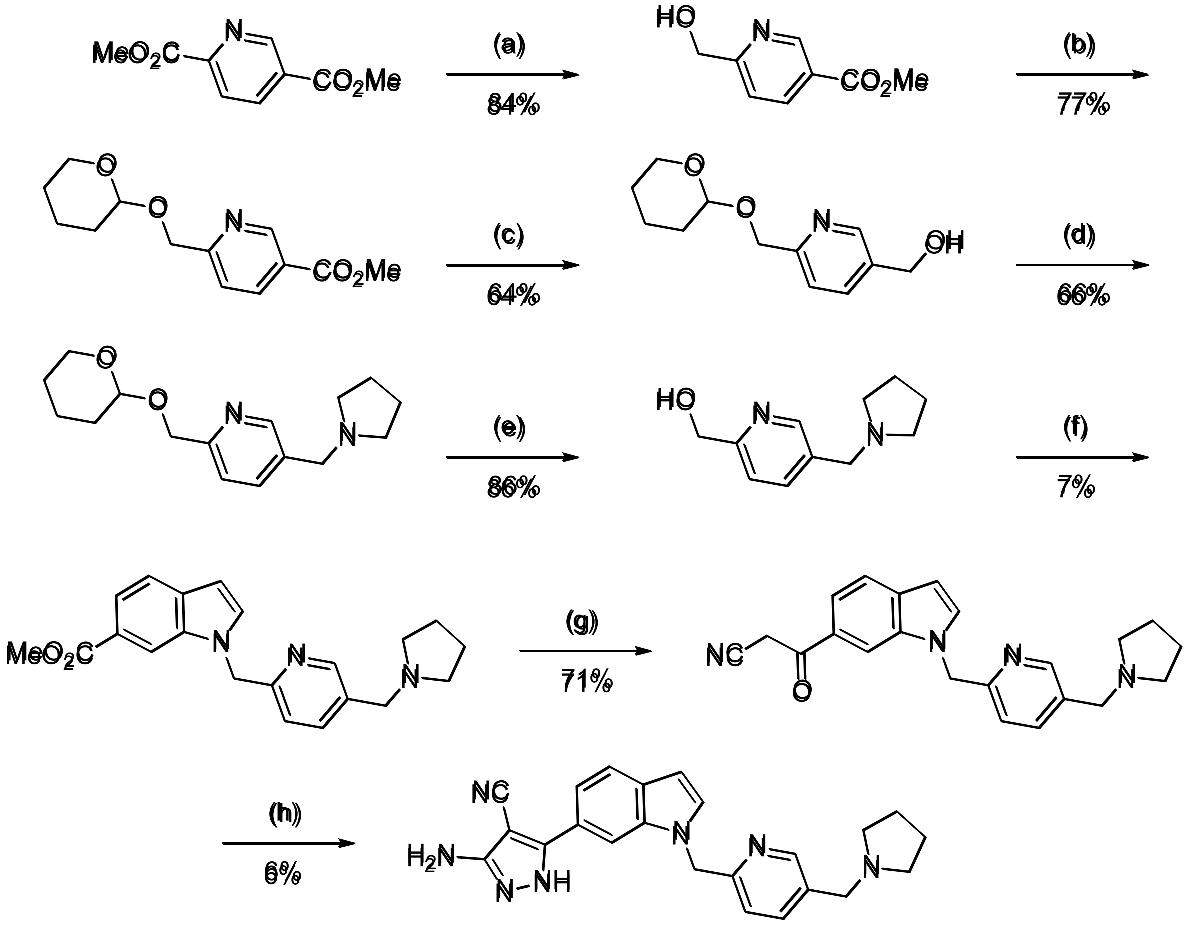

Scheme S3: Reagents and conditions: (a) sodium borohydride, $CaCl_2$, methanol, THF, 0 °C; (b) methanesulfonic acid, 3,4-dihydro-2H-pyran, DCM; (c) lithium aluminium hydride (2.4 M in THF), THF, 0 °C; (d) (i) methanesulfonyl chloride, triethylamine, DCM, 0 °C to room temperature (ii) pyrrolidine, caesium carbonate, DMF; (e) p-toluenesulfonic acid monohydrate, ethanol, 50 °C; (f) (i) methanesulfonyl chloride, triethylamine, DCM, 0 °C to room temperature (ii) methyl 1H-indole-6-carboxylate, sodium hydride, NaI, DMF, 0 to 60 °C (iii) methanol, sulfuric acid, reflux; (g) acetonitrile, n-butyllithium (1.6 M in hexanes), THF, -78 °C; (h) (i) trichloroacetonitrile, sodium acetate, ethanol (ii) hydrazine monohydrate, ethanol, reflux.

methyl 6-(hydroxymethyl)nicotinate (9)

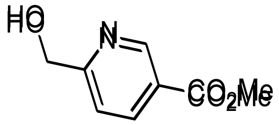

Sodium borohydride (0.727 g, 19.2 mmol) was added portionwise at 0 °C to a suspension of dimethyl pyridine-2,5-dicarboxylate (2.50 g, 12.8 mmol) and $CaCl_2$ (5.69 g, 51.2 mmol) in methanol/THF (2:1, 90 mL). The reaction mixture was stirred at 0 °C over 90 minutes, then diluted with water (50 mL) dropwise at 0 °C. The product was extracted into chloroform (3 x 50 mL). The combined organic extracts were washed (brine), dried ($MgSO_4$) and concentrated *in vacuo* to afford methyl 6-(hydroxymethyl)nicotinate (1.80 g, 84% yield).

^{1}H NMR (400 MHz, $CDCl_3$) 9.15 (1H, d, J = 1.4 Hz), 8.29 (1H, dd, J = 8.2, 2.1 Hz), 7.36 (1H, d, J = 8.2 Hz), 4.83 (2H, d, J = 3.6 Hz), 3.95 (3H, s), 3.75-3.65 (1H, m); ^{13}C NMR (100 MHz, $CDCl_3$) 165.8, 163.7, 150.1, 137.9, 125.1, 120.1, 64.4, 52.6; spectroscopic data consistent with literature (9).

methyl 6-(((tetrahydro-2H-pyran-2-yl)oxy)methyl)nicotinate

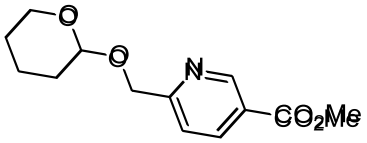

Methanesulfonic acid (0.763 mL, 11.8 mmol) was added to a solution of methyl 6-(hydroxymethyl)nicotinate (1.79 g, 10.7 mmol) and 3,4-dihydro-2H-pyran (1.95 mL, 21.4 mmol) in DCM (20 mL). The reaction mixture was stirred over 150 minutes, then washed with $NaHCO_3$ solution (2 x 15 mL) and brine (15 mL), dried ($MgSO_4$) and concentrated *in vacuo*. Purification by flash column chromatography (0 – 20% ethyl acetate in petroleum ether) gave methyl 6-(((tetrahydro-2H-pyran-2-yl)oxy)methyl)nicotinate (2.07 g, 77% yield).

^{1}H NMR (400 MHz, $CDCl_3$) 9.14 (1H, d, J = 2.1 Hz), 8.29 (1H, dd, J = 8.1, 2.2 Hz), 7.58 (1H, dd, J = 8.2, 0.7 Hz), 4.95 (1H, d, J = 14.6 Hz), 4.78 (1H, t, J = 3.5 Hz), 4.70 (1H, d, J = 14.5 Hz), 3.94 (3H, s), 3.93-3.84 (1H, m), 3.60-3.52 (1H, m), 1.97-1.47 (6H, m); ^{13}C NMR (100 MHz, $CDCl_3$) 166.0, 163.5, 150.5, 137.8, 124.7, 120.7, 98.8, 69.7, 62.5, 52.5, 30.6, 25.5, 19.5; ν_{max}/cm^{-1} 2946, 2926, 2876, 2848, 1714 (C=O), 1596; HRMS (ESI)+: m/z calculated for $[C_{13}H_{17}NO_4 + Na]^+$ = 274.1050, observed 274.1045.

(6-(((tetrahydro-2H-pyran-2-yl)oxy)methyl)pyridin-3-yl)methanol

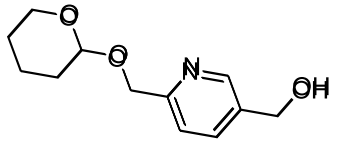

Lithium aluminium hydride (2.4 M in THF, 5.1 mL, 12 mmol) was added dropwise at 0 °C to a solution of methyl 6-(((tetrahydro-2H-pyran-2-yl)oxy)methyl)nicotinate (2.06 g, 8.20 mmol) in THF (20 mL). The reaction mixture was stirred at 0 °C for 45 minutes. Isopropanol (5 mL) was added dropwise at 0 °C to the reaction mixture, followed by water (50 mL). The product was extracted into DCM/methanol (9:1, 3 x 100 mL). The combined organic extracts were washed (brine), dried ($MgSO_4$) and concentrated *in vacuo*. Purification by flash column chromatography (60 – 90% ethyl acetate in petroleum ether) gave (6-(((tetrahydro-2H-pyran-2-yl)oxy)methyl)pyridin-3-yl)methanol (1.16 g, 64% yield).

^{1}H NMR (400 MHz, $CDCl_3$) 8.50 (1H, d, J = 1.7 Hz), 7.74 (1H, dd, J = 7.9, 2.2 Hz), 7.48 (1H, d, J = 8.1 Hz), 4.90 (1H, d, J = 13.3 Hz), 4.78 (1H, t, J = 3.6 Hz), 4.73 (2H, s), 4.65 (1H, d, J = 13.3 Hz), 3.97-3.88 (1H, m), 3.61-3.53 (1H, m), 2.60 (1H, br s), 1.91-1.49 (6H, m); ^{13}C NMR (100 MHz, $CDCl_3$) 158.0, 148.0, 135.7, 135.0, 121.5, 98.7, 69.8, 62.7, 62.4, 30.7, 25.5, 19.5; v_{max}/cm^{-1} 3300 (br, O-H), 2939, 2869, 1604, 1574; HRMS (ESI)+: m/z calculated for $[C_{12}H_{17}NO_3 + H]^+$ = 224.1281, observed 224.1284.

5-(pyrrolidin-1-ylmethyl)-2-(((tetrahydro-2H-pyran-2-yl)oxy)methyl)pyridine

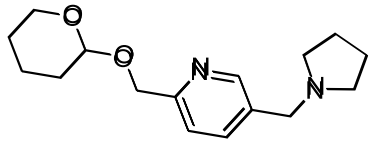

Methanesulfonyl chloride (0.478 mL, 6.18 mmol) was added dropwise at 0 °C to a solution of (6-(((tetrahydro-2H-pyran-2-yl)oxy)methyl)pyridin-3-yl)methanol (1.15 g, 5.15 mmol) and triethylamine (0.933 mL, 6.70 mmol) in DCM (10 mL). The reaction mixture was warmed to room temperature and stirred over 1 hour. The reaction was diluted with $NaHCO_3$ solution (25 mL) and extracted into DCM (3 x 50 mL). The combined organic extracts were washed (brine), dried ($MgSO_4$) and concentrated *in vacuo*. The crude residue was dissolved in DMF (10 mL), then pyrrolidine (0.516 mL, 6.18 mmol) and caesium carbonate (2.18 g, 6.70 mmol) were added. The reaction mixture was stirred over 14 hours, then diluted with ethyl acetate (100 mL), washed with Na_2CO_3 solution (2 x 100 mL) and brine (100 mL), dried ($MgSO_4$) and concentrated *in vacuo*. Purification by flash column chromatography (0 – 8% methanol in DCM) gave 5-(pyrrolidin-1-ylmethyl)-2-(((tetrahydro-2H-pyran-2-yl)oxy)methyl)pyridine (0.970 g, 66% yield).

LCMS (ESI+): m/z 277.3 [M + H]$^+$, rt 0.65 minutes, 97%; ^{1}H NMR (400 MHz, $CDCl_3$) 8.48 (1H, s), 7.68 (1H, dd, J = 7.9, 1.7 Hz), 7.42 (1H, d, J = 7.9 Hz), 4.88 (1H, d, J = 13.2 Hz), 4.77 (1H, t, J = 3.4 Hz), 4.62 (1H, d, J = 13.3 Hz), 3.96-3.85 (1H, m), 3.61 (2H, s), 3.58-3.51 (1H, m), 2.55-2.43 (4H, m), 1.95-1.48 (10H, m); ^{13}C NMR (100 MHz, $CDCl_3$) 157.3, 149.6, 137.3, 133.4, 121.3, 98.6, 69.9, 62.3, 57.8, 54.2, 30.7, 25.5, 23.5, 19.5; v_{max}/cm^{-1} 2939, 2783, 1602, 1572; HRMS (ESI)+: m/z calculated for $[C_{16}H_{24}N_2O_2 + H]^+$ = 277.1911, observed 277.1906.

(5-(pyrrolidin-1-ylmethyl)pyridin-2-yl)methanol

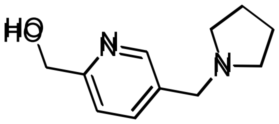

p-Toluenesulfonic acid monohydrate (1.28 g, 6.74 mmol) was added to a solution of 5-(pyrrolidin-1-ylmethyl)-2-(((tetrahydro-2H-pyran-2-yl)oxy)methyl)pyridine (0.960 g, 3.37 mmol) in ethanol (10 mL). The reaction mixture was heated to 50 °C over 30 minutes, then diluted with water (15 mL), adjusted to pH 14 using aqueous NaOH (10% w/v) and extracted into DCM (6 x 25 mL). The combined organic extracts were washed (brine), dried ($MgSO_4$) and concentrated *in vacuo*. Purification by flash column chromatography (0 – 15% methanol (+ 0.1% NH_3) in DCM) gave (5-(pyrrolidin-1-ylmethyl)pyridin-2-yl)methanol (0.560 g, 86% yield).

^{1}H NMR (400 MHz, $CDCl_3$) 8.48 (1H, d, J = 1.5 Hz), 7.69 (1H, dd, J = 7.9, 2.0 Hz), 7.21 (1H, d, J = 8.1 Hz), 4.74 (2H, s), 3.62 (2H, s), 2.57-2.44 (4H, m), 1.85-1.73 (4H, m); ^{13}C NMR (100 MHz, $CDCl_3$) 158.0, 148.9, 137.5, 133.6, 120.3, 64.2, 57.7, 54.2, 23.6; v_{max}/cm^{-1} 3200 (br, O-H), 2963, 2794, 1603, 1572; HRMS (ESI)+: m/z calculated for $[C_{11}H_{16}N_2O + H]^+$ = 193.1335, observed 193.1335.

methyl 1-((5-(pyrrolidin-1-ylmethyl)pyridin-2-yl)methyl)-1H-indole-6-carboxylate

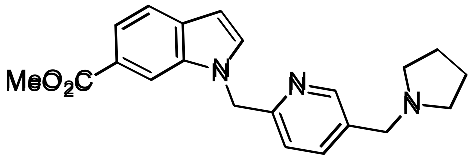

Methanesulfonyl chloride (0.242 mL, 3.12 mmol) was added dropwise at 0 °C to a solution of (5-(pyrrolidin-1-ylmethyl)pyridin-2-yl)methanol (0.500 g, 2.60 mmol) and triethylamine (0.471 mL, 3.38 mmol) in DCM (10 mL). The reaction mixture was warmed to room temperature and stirred over 150 minutes. Na_2CO_3 solution (15 mL) was added dropwise at 0 °C to the reaction mixture. The intermediate was extracted into DCM (4 x 25 mL). The combined organic extracts were washed (brine), dried ($MgSO_4$) and concentrated *in vacuo* to afford the intermediate as a crude residue. A solution of methyl 1H-indole-6-carboxylate (0.547 g, 3.12 mmol) in DMF

(3 mL) was added dropwise at 0 °C to a suspension of sodium hydride (60% in mineral oil, 0.520 g, 13.0 mmol) in DMF (2 mL). The reaction mixture was warmed to room temperature over 30 minutes. The crude residue was dissolved in DMF (5 mL) and added dropwise at 0 °C to the reaction mixture. The reaction mixture was warmed to room temperature and stirred over 45 minutes. Sodium iodide (39 mg, 0.26 mmol) was added to the reaction mixture, and it was heated to 60 °C over 30 minutes. Methanol (150 mL) was added dropwise at 0 °C to the reaction mixture followed by sulfuric acid (10 mL). The reaction mixture was heated under reflux for 14 hours, then concentrated *in vacuo*. Na_2CO_3 solution (200 mL) was added dropwise to the reaction mixture, followed by water (50 mL). The product was extracted into ethyl acetate (2 x 200 mL), followed by DCM/methanol (9:1, 3 x 200 mL). The combined organic extracts were washed (brine), dried ($MgSO_4$) and concentrated *in vacuo*. Purification by flash column chromatography (0 – 100% ethyl acetate in petroleum ether, 0 – 10% methanol in DCM) gave methyl 1-((5-(pyrrolidin-1-ylmethyl)pyridin-2-yl)methyl)-1H-indole-6-carboxylate (95 mg, 7% yield).

LCMS (ESI+): m/z 350.3 $[M + H]^+$, rt 1.39 minutes, 84%; ^1H NMR (400 MHz, $CDCl_3$) 8.46 (1H, d, J = 1.6 Hz), 8.07 (1H, s), 7.82 (1H, dd, J = 8.4, 1.4 Hz), 7.66 (1H, d, J = 8.4 Hz), 7.36 (1H, d, J = 7.9 Hz), 7.31 (1H, dd, J = 8.0, 2.2 Hz), 7.27-7.24 (1H, m), 6.61 (1H, dd, J = 3.1, 0.8 Hz), 5.39 (2H, s), 3.92 (3H, s), 3.79 (2H, s), 2.61 (4H, br s), 1.87-1.75 (4H, m); ^{13}C NMR (100 MHz, $CDCl_3$) 168.0, 158.4, 147.6, 135.5, 135.1, 132.4, 131.10, 131.08, 123.6, 123.4, 120.9, 120.7, 111.7, 102.7, 61.5, 54.1, 52.0, 47.4, 23.5; v_{max}/cm^{-1} 2951, 2799, 1705 (C=O), 1615, 1602, 1569, 1505; HRMS (ESI)+: m/z calculated for $[C_{21}H_{23}N_3O_2 + H]^+$ = 350.1863, observed 350.1863.

3-oxo-3-(1-((5-(pyrrolidin-1-ylmethyl)pyridin-2-yl)methyl)-1H-indol-6-yl)propanenitrile

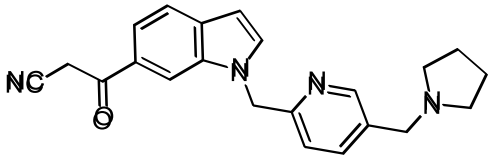

n-Butyllithium (1.6 M in hexanes, 0.42 mL, 0.67 mmol) was added dropwise at -78 °C to a mixture of acetonitrile (58 µL, 1.1 mmol) and THF (2 mL). The reaction mixture was stirred at -78 °C over 30 minutes. A solution of methyl 1-((5-(pyrrolidin-1-ylmethyl)pyridin-2-yl)methyl)-1H-indole-6-carboxylate (92 mg, 0.22 mmol) in THF (3 mL) was added dropwise at -78 °C over 30 minutes. The reaction mixture was stirred at -78 °C over 1 hour. Water (15 mL) was added dropwise at 0 °C, and the reaction mixture adjusted to pH 8. The product was extracted into DCM (3 x 25 mL), followed by DCM/methanol (9:1, 3 x 25 mL). The combined organic extracts were washed (brine), dried ($MgSO_4$) and concentrated *in vacuo*. Purification by flash column chromatography (0 – 20% methanol in DCM) gave 3-oxo-3-(1-((5-(pyrrolidin-1-ylmethyl)pyridin-2-yl)methyl)-1H-indol-6-yl)propanenitrile (69 mg, 71% yield).

LCMS (ESI+): m/z 359.2 $[M + H]^+$, (ESI-): m/z 357.1 $[M - H]^-$, rt 1.30 minutes, 81%; ^{1}H NMR (400 MHz, $CDCl_3$) 8.38 (1H, s), 7.96 (1H, s), 7.66 (1H, d, J = 8.4 Hz), 7.56 (1H, d, J = 8.3 Hz), 7.42-7.29 (3H, m), 6.60 (1H, d, J = 2.8 Hz), 5.37 (2H, s), 4.13 (2H, br s), 3.79 (2H, s), 2.70-2.55 (4H, m), 1.85-1.70 (4H, m); ^{13}C NMR (100 MHz, $CDCl_3$) 187.1, 158.3, 147.8, 135.7, 135.2, 133.7, 132.9, 130.8, 128.3, 123.5, 121.4, 119.8, 114.6, 110.9, 103.1, 61.4, 54.2, 47.7, 29.8, 23.5; v_{max}/cm^{-1} 2919, 2799, 2168 (C≡N), 1675 (C=O), 1605; HRMS (ESI)+: m/z calculated for $[C_{22}H_{22}N_4O + H]^+$ = 359.1866, observed 359.1866.

3-amino-5-(1-((5-(pyrrolidin-1-ylmethyl)pyridin-2-yl)methyl)-1H-indol-6-yl)-1H-pyrazole-4-carbonitrile (AW4)

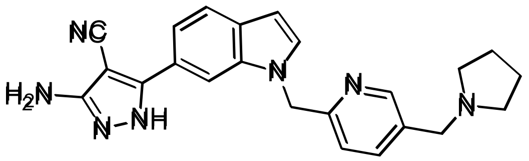

Trichloroacetonitrile (45 µL, 0.45 mmol) was added to a suspension of 3-oxo-3-(1-((5-(pyrrolidin-1-ylmethyl)pyridin-2-yl)methyl)-1H-indol-6-yl)propanenitrile (66 mg, 0.15 mmol) and sodium acetate (61 mg, 0.75 mmol) in ethanol (4 mL). The reaction mixture was stirred over 36 hours, then Na_2CO_3 solution (12.5 mL) and water (12.5 mL) were added. The intermediate was extracted into DCM (2 x 25 mL) and methanol/DCM (9:1, 4 x 25 mL). The combined organic extracts were washed (brine), dried ($MgSO_4$) and concentrated *in vacuo*. The crude residue was dissolved in ethanol (5 mL) and hydrazine monohydrate (73 µL, 1.5 mmol) was added. The reaction mixture was heated under reflux for 24 hours, then quenched with excess acetone at room temperature and concentrated *in vacuo*. Purification by flash column chromatography (0 – 20% methanol (+ 0.1% NH_3) in DCM) gave 3-amino-5-(1-((5-(pyrrolidin-1-ylmethyl)pyridin-2-yl)methyl)-1H-indol-6-yl)-1H-pyrazole-4-carbonitrile (3.6 mg, 6% yield).

LCMS (ESI+): m/z 398.3 $[M + H]^+$, (ESI-): m/z 396.2 $[M - H]^-$, rt 1.32 minutes, 100%; ^{1}H NMR (500 MHz, CD_3OD) 8.41 (1H, d, J = 1.9 Hz), 7.83 (1H, s), 7.66 (1H, d, J = 8.4 Hz), 7.64 (1H, dd, J = 8.1, 2.3 Hz), 7.51 (1H, d, J = 7.9 Hz), 7.47 (1H, d, J = 3.2 Hz), 7.42 (1H, d, J = 8.2 Hz), 6.58 (1H, d, J = 3.1 Hz), 5.49 (2H, s), 3.79 (2H, s), 2.67-2.59 (4H, m), 1.83-1.76 (4H, m); ^{13}C NMR (125 MHz, CD_3OD) 158.3, 148.5, 137.5, 137.2, 134.2, 131.5, 125.0, 122.4, 119.0, 117.0, 109.0, 103.2, 61.9, 55.1, 48.2, 24.2 (5 peaks missing); v_{max}/cm^{-1} 3150 (br, N-H), 2918, 2209 (C≡N), 1602, 1501; HRMS (ESI)+: m/z calculated for $[C_{23}H_{23}N_7 + H]^+$ = 398.2088, observed 398.2082.

Synthesis of AW5-AW7

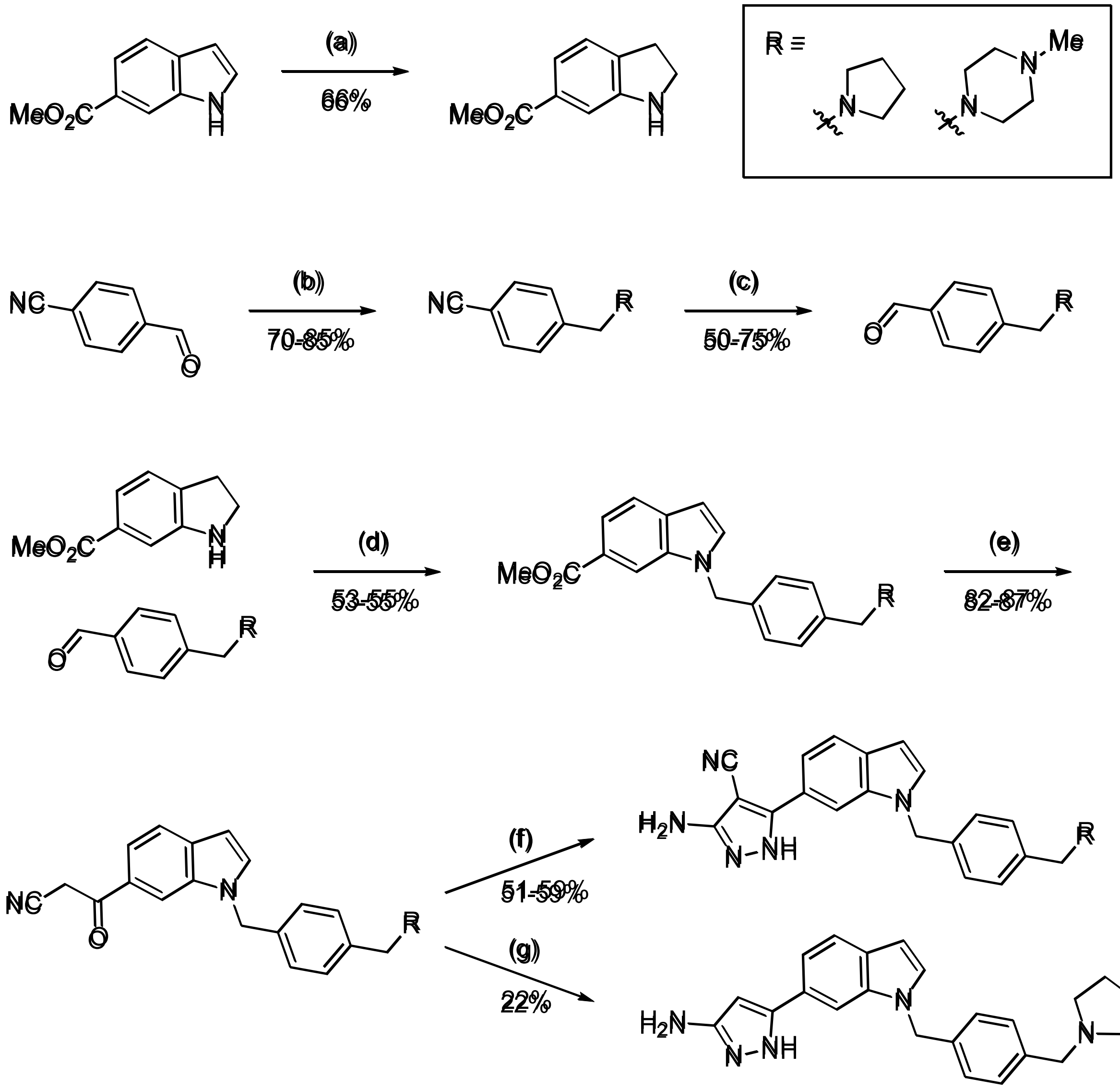

Scheme S4: *Reagents and conditions: (a) sodium cyanoborohydride, acetic acid, 0 °C to room temperature; (b) sodium triacetoxyborohydride, RH, acetic acid, DCM; (c) diisobutylaluminium hydride (1 M in THF), THF, 0 °C to room temperature; (d) benzoic acid, toluene, 200 °C µW; (e) acetonitrile, n-butyllithium (1.6 M in hexanes), THF, -78 °C; (f) (i) trichloroacetonitrile, sodium acetate, ethanol (ii) hydrazine monohydrate, ethanol, reflux; (g) hydrazine monohydrate, ethanol, reflux.*

methyl indoline-6-carboxylate (10)

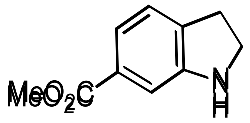

Sodium cyanoborohydride (1.61 g, 25.7 mmol) was added portionwise at 0 °C to a solution of methyl 1H-indole-6-carboxylate (1.50 g, 8.56 mmol) in acetic acid (15 mL) over 30 minutes. The reaction mixture was warmed to room temperature and stirred over 7 hours. The reaction was diluted with ethyl acetate (100 mL), washed with $NaHCO_3$ solution (3 x 100 mL) and brine (100 mL), dried ($MgSO_4$) and concentrated *in vacuo*. Purification by flash column chromatography (0 – 30% ethyl acetate in petroleum ether) gave methyl indoline-6-carboxylate (1.11 g, 66% yield).

LCMS (ESI+): m/z 178.2 $[M + H]^+$, rt 1.00 minutes, 90%; ^1H NMR (400 MHz, $CDCl_3$) 7.40 (1H, dd, J = 7.6, 1.4 Hz), 7.23 (1H, d, J = 1.4 Hz), 7.12 (1H, d, J = 7.6 Hz), 3.90 (1H, br s), 3.86 (3H, s), 3.57 (2H, t, J = 8.4 Hz), 3.04 (2H, t, J = 8.4 Hz); ^{13}C NMR (100 MHz, $CDCl_3$) 167.6, 151.9, 135.0, 129.4, 124.3, 120.7, 109.6, 52.0, 47.5, 29.9; ^1H NMR spectroscopic data consistent with literature (10).

4-(pyrrolidin-1-ylmethyl)benzonitrile

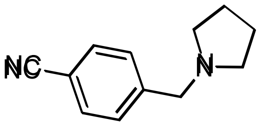

Sodium triacetoxyborohydride (1.29 g, 6.10 mmol) was added to a solution of 4-formylbenzonitrile (0.500 g, 3.81 mmol), pyrrolidine (0.318 mL, 3.81 mmol) and acetic acid (0.262 mL, 4.58 mmol) in DCM (10 mL). The reaction mixture was stirred over 15 hours. The reaction was diluted with water (25 mL), adjusted to pH 14 using aqueous NaOH (6 M) and extracted into DCM (3 x 25 mL). The combined organic extracts were dried ($MgSO_4$) and concentrated *in vacuo*. Purification by flash column chromatography (0 – 5% methanol in DCM) gave 4-(pyrrolidin-1-ylmethyl)benzonitrile (0.645 g, 85% yield).

^1H NMR (400 MHz, $CDCl_3$) 7.62-7.56 (2H, m), 7.47-7.41 (2H, m), 3.65 (2H, s), 2.55-2.44 (4H, m), 1.84-1.73 (4H, m); ^{13}C NMR (100 MHz, $CDCl_3$) 145.4, 132.2, 129.4, 119.2, 110.8, 60.3, 54.4, 23.6; spectroscopic data consistent with literature (11).

4-(pyrrolidin-1-ylmethyl)benzaldehyde

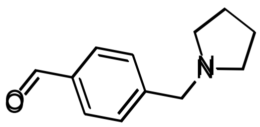

Diisobutylaluminium hydride (1 M in THF, 2.2 mL, 2.2 mmol) was added dropwise at 0 °C to a solution of 4-(pyrrolidin-1-ylmethyl)benzonitrile (0.408 g, 2.19 mmol) in THF (5 mL). The reaction mixture was warmed to room temperature and stirred over 45 minutes. Further diisobutylaluminium hydride (2.2 mL, 2.2 mmol) was added dropwise at 0 °C to the reaction mixture. The reaction mixture was warmed to room temperature and stirred over 15 minutes, then $NaHCO_3$ solution (50 mL) was added dropwise at 0 °C. The product was extracted into DCM/methanol (10:1, 3 x 50 mL). The combined organic extracts were washed (brine), dried ($MgSO_4$) and concentrated *in vacuo*. Purification by flash column chromatography (0 – 4% methanol (+ 0.1% NH_3) in DCM) gave 4-(pyrrolidin-1-ylmethyl)benzaldehyde (0.313 g, 75% yield).

^{1}H NMR (400 MHz, $CDCl_3$) 9.99 (1H, s), 7.83 (2H, d, J = 8.2 Hz), 7.50 (2H, d, J = 8.0 Hz), 3.68 (2H, s), 2.56-2.48 (4H, m), 1.84-1.75 (4H, m); ^{13}C NMR (100 MHz, $CDCl_3$) 192.2, 147.0, 135.5, 129.9, 129.4, 60.6, 54.4, 23.7; spectroscopic data consistent with literature (12).

methyl 1-(4-(pyrrolidin-1-ylmethyl)benzyl)-1H-indole-6-carboxylate

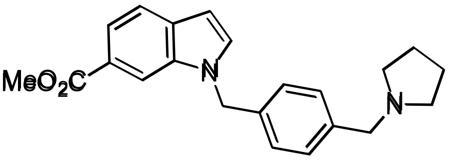

A solution of 4-(pyrrolidin-1-ylmethyl)benzaldehyde (0.302 g, 1.60 mmol) in toluene (3 mL) was added to a mixture of methyl indoline-6-carboxylate (0.377 g, 1.91 mmol) and benzoic acid (39 mg, 0.32 mmol). The reaction mixture was heated to 200 °C by microwave for 20 minutes. The reaction was diluted with ethyl acetate (50 mL), washed with $NaHCO_3$ solution (3 x 50 mL) and brine (50 mL), dried ($MgSO_4$) and concentrated *in vacuo*. Purification by flash column chromatography (40 – 80% ethyl acetate in petroleum ether) gave methyl 1-(4-(pyrrolidin-1-ylmethyl)benzyl)-1H-indole-6-carboxylate (0.297 g, 53% yield).

LCMS (ESI+): m/z 349.3 $[M + H]^+$, rt 1.51 minutes, 100%; ^{1}H NMR (400 MHz, $CDCl_3$) 8.11 (1H, s), 7.81 (1H, dd, J = 8.3, 1.3 Hz), 7.66 (1H, d, J = 8.4 Hz), 7.32-7.23 (3H, m), 7.07 (2H, d, J = 7.9 Hz), 6.59 (1H, dd, J = 3.1, 0.5 Hz), 5.37 (2H, s), 3.92 (3H, s), 3.58 (2H, s), 2.52-2.44 (4H, m), 1.80-1.73 (4H, m); ^{13}C NMR (100 MHz, $CDCl_3$) 168.3, 139.3, 135.9, 135.6, 132.4, 131.5, 129.5, 126.9, 123.5, 120.72, 120.65, 112.1, 102.2, 60.4, 54.3, 52.0, 50.0, 23.6; ν_{max}/cm^{-1} 2951, 2782, 1706 (C=O), 1615, 1505; HRMS (ESI)+: m/z calculated for $[C_{22}H_{24}N_2O_2 + H]^+$ = 349.1911, observed 349.1913.

3-oxo-3-(1-(4-(pyrrolidin-1-ylmethyl)benzyl)-1H-indol-6-yl)propanenitrile

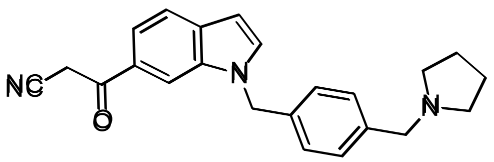

n-Butyllithium (1.6 M in hexanes, 2.7 mL, 4.3 mmol) was added dropwise at -78 °C to a mixture of acetonitrile (0.375 mL, 7.17 mmol) and THF (6 mL). The reaction mixture was stirred at -78 °C over 30 minutes. A solution of methyl 1-(4-(pyrrolidin-1-ylmethyl)benzyl)-1H-indole-6-carboxylate (0.500 g, 1.43 mmol) in THF (4 mL) was added dropwise at -78 °C over 30 minutes. The reaction mixture was stirred at -78 °C over 1 hour. Water (15 mL) was added dropwise at 0 °C, and the reaction mixture adjusted to pH 8. The product was extracted into DCM (3 x 25 mL). The combined organic extracts were washed (brine), dried ($MgSO_4$) and concentrated *in vacuo*. Purification by flash column chromatography (50 – 100% ethyl acetate in petroleum ether, 0 – 10% methanol in DCM) gave 3-oxo-3-(1-(4-(pyrrolidin-1-ylmethyl)benzyl)-1H-indol-6-yl)propanenitrile (0.448 g, 87% yield).
LCMS (ESI+): m/z 358.3 $[M + H]^+$, (ESI-): m/z 356.2 $[M - H]^-$, rt 1.38 minutes, 100%; ^{1}H NMR (400 MHz, $CDCl_3$) 7.98 (1H, s), 7.70 (1H, d, J = 8.3 Hz), 7.59 (1H, dd, J = 8.4, 1.3 Hz), 7.36 (1H, d, J = 3.1 Hz), 7.29 (2H, d, J = 8.1 Hz), 7.07 (2H, d, J = 7.9 Hz), 6.61 (1H, d, J = 3.1 Hz), 5.38 (2H, s), 4.09 (2H, br s), 3.59 (2H, s), 2.55-2.44 (4H, m), 1.83-1.71 (4H, m); ^{13}C NMR (100 MHz, $CDCl_3$) 187.0, 139.4, 136.0, 135.2, 133.9, 133.4, 129.7, 128.1, 127.0, 121.3, 119.7, 114.5, 111.3, 102.6, 60.3, 54.3, 50.3, 29.6, 23.5; v_{max}/cm^{-1} 2960, 2786, 2165 (C≡N), 1680 (C=O), 1607, 1503.

3-amino-5-(1-(4-(pyrrolidin-1-ylmethyl)benzyl)-1H-indol-6-yl)-1H-pyrazole-4-carbonitrile (AW5)

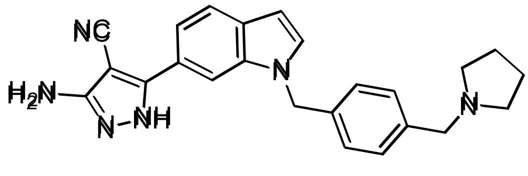

Trichloroacetonitrile (0.201 mL, 2.01 mmol) was added to a suspension of 3-oxo-3-(1-(4-(pyrrolidin-1-ylmethyl)benzyl)-1H-indol-6-yl)propanenitrile (0.239 g, 0.669 mmol) and sodium acetate (0.274 g, 3.34 mmol) in ethanol (5 mL). The reaction mixture was stirred over 4 hours, then $NaHCO_3$ solution (7.5 mL) and water (7.5 mL) were added. The intermediate was extracted into DCM (3 x 25 mL). The combined organic extracts were washed (brine), dried ($MgSO_4$) and concentrated *in vacuo*. The crude residue was dissolved in ethanol (4 mL) and hydrazine monohydrate (0.325 mL, 6.69 mmol) was added. The reaction mixture was heated under reflux for 18 hours, then quenched with excess acetone at room temperature and concentrated *in vacuo*. Purification by flash column chromatography (100% ethyl acetate, 0 –

7% methanol (+ 0.1% NH_3) in DCM) gave 3-amino-5-(1-(4-(pyrrolidin-1-ylmethyl)benzyl)-1H-indol-6-yl)-1H-pyrazole-4-carbonitrile (0.135 g, 51% yield).

LCMS (ESI+): m/z 397.3 $[M + H]^+$, (ESI-): m/z 395.3 $[M - H]^-$, rt 1.35 minutes, 100%; ^{1}H NMR (500 MHz, $CDCl_3$) 7.70 (1H, s), 7.62 (1H, d, J = 8.3 Hz), 7.42 (1H, dd, J = 8.2, 1.5 Hz), 7.21 (2H, d, J = 8.2 Hz), 7.17 (1H, d, J = 3.0 Hz), 7.04 (2H, d, J = 8.1 Hz), 6.53 (1H, dd, J = 3.1, 0.8 Hz), 5.18 (2H, s), 4.40 (2H, br s), 3.55 (2H, s), 2.55-2.42 (4H, m), 1.75-1.64 (4H, m); ^{13}C NMR (125 MHz, $CDCl_3$) 156.7, 152.0, 150.4, 138.5, 136.2, 136.0, 130.4, 130.1, 129.7, 127.2, 121.8, 117.9, 115.9, 108.0, 102.2, 75.4, 60.3, 54.2, 50.1, 23.4; v_{max}/cm^{-1} 3100 (br, N-H), 2928, 2799, 2208 (C≡N), 1620, 1589, 1501; HRMS (ESI+): m/z calculated for $[C_{24}H_{24}N_6 + H]^+$ = 397.2135, observed 397.2132.

5-(1-(4-(pyrrolidin-1-ylmethyl)benzyl)-1H-indol-6-yl)-1H-pyrazol-3-amine (AW6)

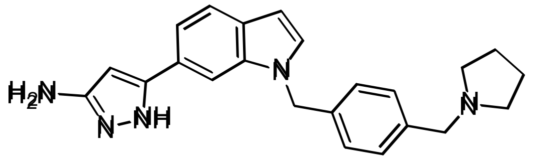

Hydrazine monohydrate (0.157 mL, 3.22 mmol) was added to a solution of 3-oxo-3-(1-(4-(pyrrolidin-1-ylmethyl)benzyl)-1H-indol-6-yl)propanenitrile (0.115 g, 0.322 mmol) in ethanol (15 mL). The reaction mixture was heated under reflux for 12 hours, then concentrated *in vacuo*. Purification by reverse phase column chromatography (0 – 100% acetonitrile in water, 0 – 65% methanol in acetonitrile) gave 5-(1-(4-(pyrrolidin-1-ylmethyl)benzyl)-1H-indol-6-yl)-1H-pyrazol-3-amine (27 mg, 22% yield).

LCMS (ESI+): m/z 372.5 $[M + H]^+$, rt 1.22 minutes, 96%; ^{1}H NMR (500 MHz, $CDCl_3$) 7.63 (1H, d, J = 8.2 Hz), 7.43 (1H, s), 7.30-7.25 (1H, m), 7.24 (2H, d, J = 8.2 Hz), 7.13 (1H, d, J = 3.1 Hz), 7.03 (2H, d, J = 8.1 Hz), 6.53 (1H, dd, J = 3.1, 0.5 Hz), 5.87 (1H, s), 5.26 (2H, s), 3.68 (2H, br s), 3.55 (2H, s), 2.53-2.38 (4H, m), 1.81-1.67 (4H, m); ^{13}C NMR (125 MHz, $CDCl_3$) 155.0 (br), 146.7 (br), 139.1, 136.6, 135.9, 129.6, 129.5, 129.0, 126.9, 124.0, 121.5, 117.7, 106.8, 102.0, 90.5, 60.4, 54.3, 50.0, 23.5; v_{max}/cm^{-1} 3150 (br, N-H), 2963, 2789, 1587, 1505; HRMS (ESI+): m/z calculated for $[C_{23}H_{25}N_5 + H]^+$ = 372.2183, observed 372.2173.

4-((4-methylpiperazin-1-yl)methyl)benzonitrile

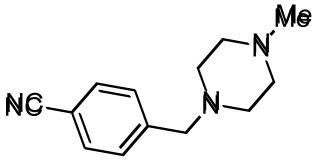

Sodium triacetoxyborohydride (2.59 g, 12.2 mmol) was added to a solution of 4-formylbenzonitrile (1.00 g, 7.63 mmol), 1-methylpiperazine (0.846 mL, 7.63 mmol) and acetic acid (0.524 mL, 9.15 mmol) in DCM (10 mL). The reaction mixture was stirred over 80 minutes. The reaction was diluted with water (10 mL), adjusted to pH 14 using aqueous NaOH (10% w/v) and extracted into DCM (3 x 25 mL). The combined organic extracts were dried ($MgSO_4$) and concentrated *in vacuo*. Purification by flash column chromatography (0 – 4% methanol in DCM) gave 4-((4-methylpiperazin-1-yl)methyl)benzonitrile (1.28 g, 70% yield).

^1H NMR (400 MHz, $CDCl_3$) 7.61-7.56 (2H, m), 7.44 (2H, d, J = 8.2 Hz), 3.53 (2H, s), 2.45 (8H, br s), 2.27 (3H, s); ^{13}C NMR (100 MHz, $CDCl_3$) 144.4, 132.2, 129.6, 119.1, 111.0, 62.5, 55.2, 53.3, 46.1; spectroscopic data consistent with literature (13).

4-((4-methylpiperazin-1-yl)methyl)benzaldehyde

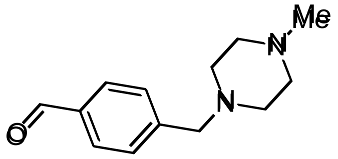

Diisobutylaluminium hydride (1 M in THF, 5.3 mL, 5.3 mmol) was added dropwise at 0 °C to a solution of 4-((4-methylpiperazin-1-yl)methyl)benzonitrile (1.26 g, 5.27 mmol) in THF (10 mL). The reaction mixture was warmed to room temperature and stirred over 30 minutes. Further diisobutylaluminium hydride (5.3 mL, 5.3 mmol) was added dropwise at 0 °C to the reaction mixture. The reaction mixture was warmed to room temperature and stirred over 30 minutes, then $NaHCO_3$ solution (50 mL) was added dropwise at 0 °C. The product was extracted into DCM/methanol (9:1, 3 x 50 mL). The combined organic extracts were washed (brine), dried ($MgSO_4$) and concentrated *in vacuo*. Purification by flash column chromatography (0 – 4% methanol (+ 0.1% NH_3) in DCM) gave 4-((4-methylpiperazin-1-yl)methyl)benzaldehyde (0.576 g, 50% yield).

^1H NMR (400 MHz, $CDCl_3$) 9.98 (1H, s), 7.85-7.79 (2H, m), 7.50 (2H, d, J = 8.0 Hz), 3.57 (2H, s), 2.47 (8H, br s), 2.28 (3H, s); ^{13}C NMR (100 MHz, $CDCl_3$) 192.1, 146.0, 135.6, 129.9, 129.6, 62.8, 55.2, 53.3, 46.2; spectroscopic data consistent with literature (14).

methyl 1-(4-((4-methylpiperazin-1-yl)methyl)benzyl)-1H-indole-6-carboxylate

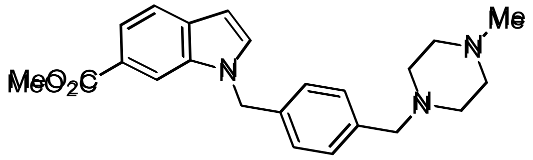

Toluene (3 mL) was added to a mixture of 4-((4-methylpiperazin-1-yl)methyl)benzaldehyde (0.300 g, 1.37 mmol), methyl indoline-6-carboxylate (0.314 g, 1.65 mmol) and benzoic acid (34 mg, 0.28 mmol). The reaction mixture was heated to 200 °C by microwave for 30 minutes. The reaction was diluted with ethyl acetate (25 mL), washed with $NaHCO_3$ solution (3 x 15 mL) and brine (15 mL), dried ($MgSO_4$) and concentrated *in vacuo*. Purification by flash column chromatography (0 – 10% methanol in DCM) gave methyl 1-(4-((4-methylpiperazin-1-yl)methyl)benzyl)-1H-indole-6-carboxylate (0.329 g, 55% yield).

LCMS (ESI+): m/z 378.3 $[M + H]^+$, rt 1.46 minutes, 100%; ^1H NMR (400 MHz, $CDCl_3$) 8.12 (1H, s), 7.83 (1H, dd, J = 8.4, 1.4 Hz), 7.68 (1H, d, J = 8.4 Hz), 7.30-7.24 (3H, m), 7.08 (2H, d, J = 8.2 Hz), 6.61 (1H, dd, J = 3.1, 0.7 Hz), 5.38 (2H, s), 3.93 (3H, s), 3.49 (2H, s), 2.49 (8H, br s), 2.31 (3H, s); ^{13}C NMR (100 MHz, $CDCl_3$) 168.1, 137.9, 135.8, 135.7, 132.3, 131.3, 129.6, 126.7, 123.4, 120.6, 120.5, 112.0, 102.1, 62.5, 55.0, 52.8, 51.9, 49.8, 45.8; v_{max}/cm^{-1} 2937, 2794, 1706 (C=O), 1614, 1504; HRMS (ESI)+: m/z calculated for $[C_{23}H_{27}N_3O_2 + H]^+$ = 378.2176, observed 378.2159.

3-(1-(4-((4-methylpiperazin-1-yl)methyl)benzyl)-1H-indol-6-yl)-3-oxopropanenitrile

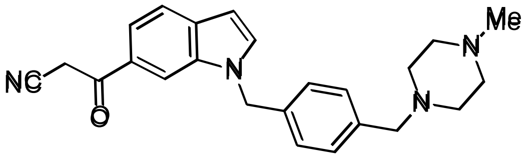

n-Butyllithium (1.6 M in hexanes, 1.4 mL, 2.2 mmol) was added dropwise at -78 °C to a mixture of acetonitrile (0.188 mL, 3.61 mmol) and THF (3 mL). The reaction mixture was stirred at -78 °C over 30 minutes. A solution of methyl 1-(4-((4-methylpiperazin-1-yl)methyl)benzyl)-1H-indole-6-carboxylate (0.313 g, 0.721 mmol) in THF (3 mL) was added dropwise at -78 °C over 30 minutes. The reaction mixture was stirred at -78 °C over 30 minutes. Water (20 mL) was added dropwise at 0 °C, and the reaction mixture adjusted to pH 8. The product was extracted into DCM (3 x 25 mL). The combined organic extracts were washed (brine), dried ($MgSO_4$) and concentrated *in vacuo*. Purification by flash column chromatography (50 – 100% ethyl acetate in petroleum ether, 0 – 10% methanol in DCM) gave 3-(1-(4-((4-methylpiperazin-1-yl)methyl)benzyl)-1H-indol-6-yl)-3-oxopropanenitrile (0.258 g, 82% yield).

LCMS (ESI+): m/z 387.2 $[M + H]^+$, rt 1.38 minutes, 89%; ^1H NMR (400 MHz, $CDCl_3$) 7.98 (1H, s), 7.69 (1H, d, J = 8.3 Hz), 7.58 (1H, dd, J = 8.4, 1.6 Hz), 7.36 (1H, d, J = 3.2 Hz), 7.26 (2H, d, J = 8.1 Hz), 7.06 (2H, d, J = 8.2 Hz), 6.61 (1H, dd, J = 3.1, 0.7 Hz), 5.37 (2H, s), 4.09 (2H, br s), 3.47 (2H, s), 2.44 (8H, br s), 2.27 (3H, s); ^{13}C NMR (100 MHz, $CDCl_3$) 187.0, 138.4,

136.0, 135.3, 133.8, 133.4, 129.9, 128.1, 126.9, 121.3, 119.7, 114.5, 111.3, 102.6, 62.6, 55.2, 53.1, 50.3, 46.1, 29.7; v_{max}/cm^{-1} 2934, 2790, 2165 (C≡N), 1680 (C=O), 1607, 1502; HRMS (ESI)-: m/z calculated for $[C_{24}H_{26}N_4O - H]^-$ = 385.2034, observed 385.2029.

3-amino-5-(1-(4-((4-methylpiperazin-1-yl)methyl)benzyl)-1H-indol-6-yl)-1H-pyrazole-4-carbonitrile (AW7)

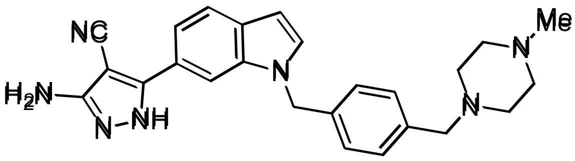

Trichloroacetonitrile (0.167 mL, 1.66 mmol) was added to a suspension of 3-(1-(4-((4-methylpiperazin-1-yl)methyl)benzyl)-1H-indol-6-yl)-3-oxopropanenitrile (0.241 g, 0.555 mmol) and sodium acetate (0.228 g, 2.77 mmol) in ethanol (5 mL). The reaction mixture was stirred over 17 hours, then $NaHCO_3$ solution (25 mL) was added. The intermediate was extracted into DCM (3 x 25 mL). The combined organic extracts were washed (brine), dried ($MgSO_4$) and concentrated *in vacuo*. The crude residue was dissolved in ethanol (10 mL) and hydrazine monohydrate (0.270 mL, 5.55 mmol) was added. The reaction mixture was heated under reflux for 24 hours, then concentrated *in vacuo*. Purification by reverse phase column chromatography (0 – 50% acetonitrile in water (+ 0.1% formic acid)) was attempted, with the resultant fractions combined, adjusted to pH 8, and extracted into DCM/methanol (9:1, 6 x 50 mL). The combined organic extracts were dried ($MgSO_4$) and concentrated *in vacuo*. Purification by flash column chromatography (0 – 20% methanol (+ 0.1% NH_3) in DCM) gave 3-amino-5-(1-(4-((4-methylpiperazin-1-yl)methyl)benzyl)-1H-indol-6-yl)-1H-pyrazole-4-carbonitrile (0.140 g, 59% yield).

LCMS (ESI+): m/z 426.4 $[M + H]^+$, (ESI-): m/z 424.3 $[M - H]^-$, rt 1.36 minutes, 100%; ^1H NMR (500 MHz, CD_3OD) 7.84-7.81 (1H, m), 7.64 (1H, d, J = 8.3 Hz), 7.49 (1H, dd, J = 8.2, 1.4 Hz), 7.39 (1H, d, J = 3.1 Hz), 7.27-7.21 (2H, m), 7.21-7.16 (2H, m), 6.53 (1H, dd, J = 3.1, 0.7 Hz), 5.37 (2H, s), 3.46 (2H, s), 2.44 (8H, br s), 2.24 (3H, s); ^{13}C NMR (125 MHz, CD_3OD) 158.0 (br), 152.5 (br), 138.3, 137.7, 137.4, 131.6, 131.4, 131.1, 128.2, 124.0 (br), 122.2, 118.7, 117.0, 109.1, 102.7, 74.0 (br), 63.3, 55.5, 53.3, 50.8, 45.8; v_{max}/cm^{-1} 3150 (br, N-H), 2919, 2808, 2207 (C≡N), 1612, 1581; HRMS (ESI+): m/z calculated for $[C_{25}H_{27}N_7 + H]^+$ = 426.2401, observed 426.2396.

References

1. Zhang, Y., Agrebi, R., Bellows, L.E., Collet, J.F., Kaever, V. and Grundling, A. (2017) Evolutionary Adaptation of the Essential tRNA Methyltransferase TrmD to the Signaling Molecule 3',5'-cAMP in Bacteria. *J Biol Chem*, **292**, 313-327.

2. Ito, T., Masuda, I., Yoshida, K., Goto-Ito, S., Sekine, S., Suh, S.W., Hou, Y.M. and Yokoyama, S. (2015) Structural basis for methyl-donor-dependent and sequence-specific binding to tRNA substrates by knotted methyltransferase TrmD. *Proc Natl Acad Sci U S A*, **112**, E4197-4205.

3. Altschul, S.F., Gish, W., Miller, W., Myers, E.W. and Lipman, D.J. (1990) Basic local alignment search tool. *J Mol Biol*, **215**, 403-410.

4. States, D.J. and Gish, W. (1994) Combined use of sequence similarity and codon bias for coding region identification. *J Comput Biol*, **1**, 39-50.

5. Sievers, F., Wilm, A., Dineen, D., Gibson, T.J., Karplus, K., Li, W., Lopez, R., McWilliam, H., Remmert, M., Soding, J. *et al.* (2011) Fast, scalable generation of high-quality protein multiple sequence alignments using Clustal Omega. *Molecular systems biology*, **7**, 539.

6. Waterhouse, A.M., Procter, J.B., Martin, D.M., Clamp, M. and Barton, G.J. (2009) Jalview Version 2--a multiple sequence alignment editor and analysis workbench. *Bioinformatics*, **25**, 1189-1191.

7. Whitehouse, A.J., Thomas, S.E., Brown, K.P., Fanourakis, A., Chan, D.S., Libardo, M.D.J., Mendes, V., Boshoff, H.I.M., Floto, R.A., Abell, C. *et al.* (2019) Development of Inhibitors against *Mycobacterium abscessus* tRNA (m(1)G37) Methyltransferase (TrmD) Using Fragment-Based Approaches. *J Med Chem*, **62**, 7210-7232.

8. Islam, S. and Larrosa, I. (2013) "On water", phosphine-free palladium-catalyzed room temperature C-H arylation of indoles. *Chemistry*, **19**, 15093-15096.

9. Soler, M., Figueras, E., Serrano-Plana, J., Gonzalez-Bartulos, M., Massaguer, A., Company, A., Martinez, M.A., Malina, J., Brabec, V., Feliu, L. *et al.* (2015) Design, Preparation, and Characterization of Zn and Cu Metallopeptides Based On Tetradentate Aminopyridine Ligands Showing Enhanced DNA Cleavage Activity. *Inorg Chem*, **54**, 10542-10558.

10. Doebelin, C., Patouret, R., Garcia-Ordonez, R.D., Chang, M.R., Dharmarajan, V., Kuruvilla, D.S., Novick, S.J., Lin, L., Cameron, M.D., Griffin, P.R. *et al.* (2016) N-Arylsulfonyl Indolines as Retinoic Acid Receptor-Related Orphan Receptor gamma (RORgamma) Agonists. *ChemMedChem*, **11**, 2607-2620.

11. Chusov, D. and List, B. (2014) Reductive amination without an external hydrogen Source. *Angew Chem Int Ed Engl*, **53**, 5199-5201.

12. Leicht, H., Huber, S., Gottker-Schnetmann, I. and Mecking, S. (2016) Allylboration as a versatile tool for the in situ post-polymerization functionalization of 1,4-cis-poly(butadiene). *Polymer Chemistry*, **7**, 7195-7198.

13. Reeves, J.T., Malapit, C.A., Buono, F.G., Sidhu, K.P., Marsini, M.A., Sader, C.A., Fandrick, K.R., Busacca, C.A. and Senanayake, C.H. (2015) Transnitrilation from Dimethylmalononitrile to Aryl Grignard and Lithium Reagents: A Practical Method for Aryl Nitrile Synthesis. *J Am Chem Soc*, **137**, 9481-9488.

14. Prati, F., De Simone, A., Armirotti, A., Summa, M., Pizzirani, D., Scarpelli, R., Bertozzi, S.M., Perez, D.I., Andrisano, V., Perez-Castillo, A. *et al.* (2015) 3,4-Dihydro-1,3,5-triazin-2(1H)-ones as the First Dual BACE-1/GSK-3beta Fragment Hits against Alzheimer's Disease. *ACS Chem Neurosci*, **6**, 1665-1682.
